# Ni-Catalyzed Electro-Reductive Cross-Electrophile Couplings of Alkyl Amine-Derived Radical Precursors with Aryl Iodides

**DOI:** 10.1021/acs.joc.3c00859

**Published:** 2023-05-23

**Authors:** Lars J. Wesenberg, Alessandra Sivo, Gianvito Vilé, Timothy Noël

**Affiliations:** † Van’t Hoff Institute for Molecular Sciences (HIMS), University of Amsterdam (UvA), Amsterdam 1098 XH, The Netherlands; ‡ Department of Chemistry, Materials, and Chemical Engineering “Giulio Natta”, Politecnico di Milano, IT-20133 Milano, Italy

## Abstract

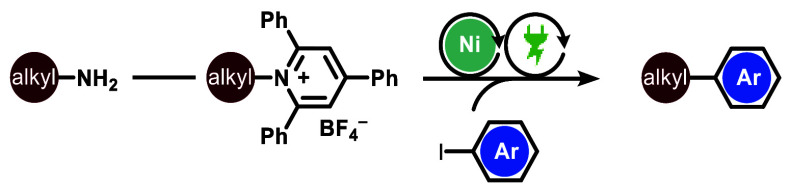

In recent years, the “Escape-from-Flatland” trend has prompted the synthetic community to develop a set of cross-coupling strategies to introduce sp^3^-carbon-based fragments in organic compounds. This study presents a novel nickel-catalyzed electrochemical methodology for reductive cross-electrophile coupling. The method enables C(sp^2^)–C(sp^3^) linkages using inexpensive amine-derived radical precursors and aryl iodides. The use of electrochemistry as a power source reduces waste and avoids chemical reductants, making this approach a more sustainable alternative to traditional cross-coupling methods.

## Introduction

Transition-metal-catalyzed cross-coupling reactions have had a major impact on the synthesis of modern pharmaceuticals, agrochemicals, and materials.^1^ The traditional cross-coupling reactions developed in the 1970s and 1980s, with Suzuki-Miyaura coupling being the leader of the pack, have focused on the forging of C(sp^2^)–C(sp^2^) linkages, resulting in a largely flat chemical space.^2−4^ In recent years, a strategy to increase the sp^3^-character of drug candidates has been pursued to increase the odds of finding new and more potent small-molecule-based treatments.^5^ This “Escape-from-Flatland” trend has prompted the synthetic community to develop a different set of cross-coupling strategies that can reliably introduce sp^3^-carbon-based fragments.^6−11^

**Figure 1:**
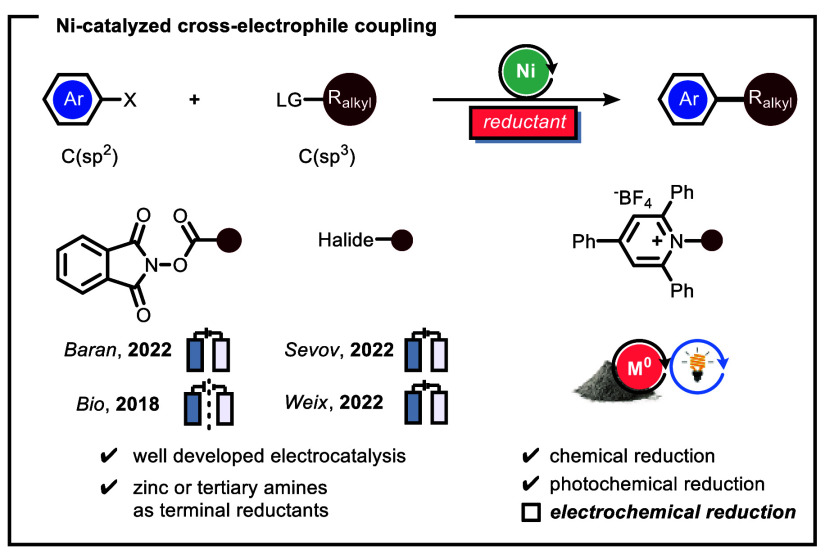
Different strategies for cross-electrophilic couplings.

Among the different strategies, cross-electrophile couplings are of particular interest due to the wide availability of bench-stable coupling partners (for example, organic halides, redox-active esters, and Katritzky salts) (Figure 1). Pioneering cross-electrophile couplings developed by groups such as Weix,^12−14^ Watson,^15−18^ Reismann,^19−21^ Gong,^22,23^ and many more^24−32^ rely on nickel-based complexes to enable the union between the two electrophiles. However, such transformations require the addition of stoichiometric amounts of terminal reductants, such as metals (Mn or Zn) or tetrakis(dimethylamino)ethylene (TDAE),^33−35^ to facilitate the overall reductive cross-coupling.

In recent years, diverse cooperative catalytic reaction strategies, which merge Ni-based cross-coupling with either photocatalysis^36−40^ or electrochemistry,^41−46^ have been developed to avoid using these stoichiometric amounts of reductants, thus increasing the sustainability of the reaction protocol.^47−49^ Electrochemical and photoelectrochemical efforts have been made to utilize specifically alkylpyridinium salts (Katritzky salts) as free radical precursors in combination with Michael acceptors and Minisci-type reactions.^50^ However, electrochemical nickel-catalyzed cross-electrophile coupling reactions exploiting these Katritzky salts have so far proven elusive.^51^ Herein, we describe our efforts to develop a Ni-based electrocatalytic cross-electrophile coupling protocol between aryl iodides and Katritzky salts. Katritzky salts are a class of bench-stable electrophiles readily prepared from the condensation reaction between alkyl amines and commercially available pyrylium salts.^52^ Hence, our method enables the formal union between two common building blocks, that is, aryl iodides and alkyl amines, which are both abundantly available in (proprietary) pharmaceutical and agrochemical compound libraries.^53,54^

## Results and Discussion

**Figure 2:**
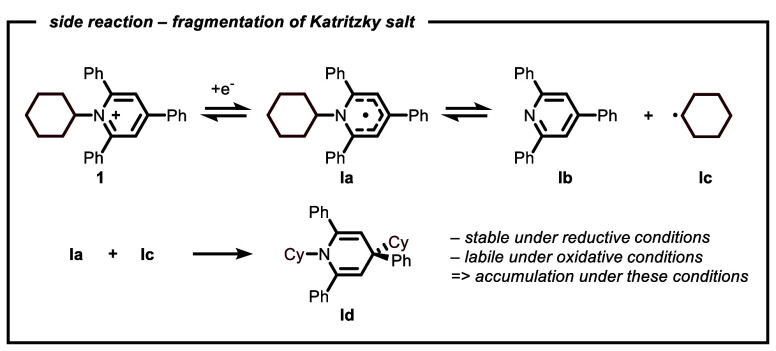
Formation of byproduct **Id** when **L3** was used as ligand.

Our experimental investigations commenced by coupling methyl 4-iodo benzoate **2** and a Katritzky salt derived from cyclohexyl amine **1**. Selected screening results are presented in Table 1, but a more detailed survey of the reaction conditions is reported in the Supporting Information (SI). For the electrochemical setup, we selected an undivided batch-cell arrangement with stainless steel as anode and nickel foam electrode (NFE) as cathode material. Using 20 mol % of NiBr_2_*x* glyme and 4,4′-dimethoxy-2,2′-bipyridine (**L1**) as ligand resulted in 76% yield of the targeted cross-coupled product when dimethylformamide (DMF) was used as a solvent and NEt_4_BF_4_ as a supporting electrolyte (Table 1, Entry 1). Under the reaction conditions outlined in Table 1, 4,4′-di*tert*-butyl-2,2′-bipyridine (**L2**) and 1,3-bis(2-pyridylimino)isoindoline (**L3**) demonstrated reduced effectiveness as ligands compared to **L1** (Table 1, Entries 2–3). Specifically, when **L2** was used, aryl–aryl dimerization became the primary source of byproduct formation, resulting in lower selectivity (Table 1, Entry 2). Alternatively, **L3** displayed lower reactivity than **L1**, but exhibited a higher selectivity toward aryl-alkyl coupling and complete suppression of homocoupling (Table 1, Entry 3). Increasing the amount of charge to 5.5 F (resulting in a reaction time of 29.5 h) showed a slight improvement in product formation, but with significantly lower productivity (Table 1, Entry 4). Additionally, a new side product (**Id**) was formed with a yield of 24% (based on the Katritzky salt), as shown in Figure 2. The formation of **Id** can be attributed to the reaction between a persistent radical (**Ia**) formed after single-electron reduction of the Katritzky salt, and a second alkyl radical (**Ic**), consuming in total two equivalents of the alkyl coupling partner. This observation suggests that the catalytic system with **L3** may not be adequate, as it appears to exhibit slow capture of the carbon-centered radical by the Ni-catalyst.^55,56^ Reducing the amount of Ni led to a less effective cross-coupling (Table 1, Entry 5), and in the complete absence of Ni, no product formation was observed (Table 1, Entry 6). Similarly, without the application of electricity, the reaction did not exhibit any reactivity (Table 1, Entry 7). We attempted to compare the practicality of using various terminal reductants, such as diisopropylethylamine (DIPEA) and triethylamine (TEA), in place of the sacrificial anode. However, the reaction did not proceed efficiently with the use of tertiary amines (Table 1, Entries 8–9).

**Table 1:**
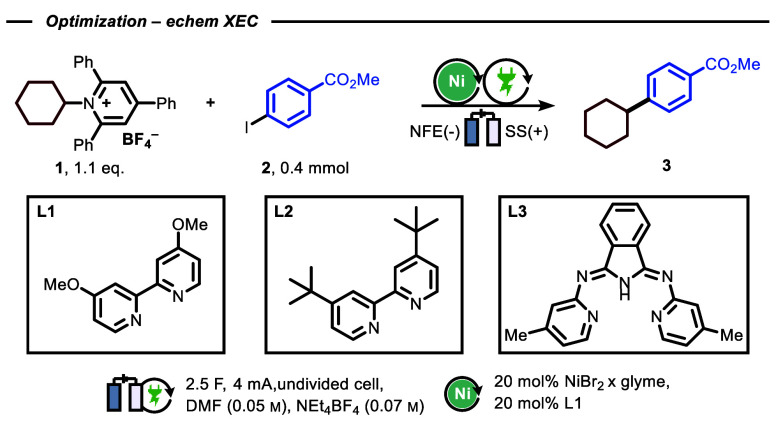
Optimization of Reaction Conditions for Electrochemical Electrophilic Cross-Coupling between Aryl Iodides and Katritzky Salts

entry	deviation from above	yield (%)^a^
1	none	76
2	L2 instead of L1	49
3	L3 instead of L1	43^b^
4	L3 instead of L1:5.5 F, 2 mA	80^c^
5	10 mol % Ni-cat.	57
6	0 mol % Ni-cat.	0
7	SS (+) w/o electricity	0
8	impervious graphite (+), DIPEA (4eq )	10^d^
9	impervious graphite (+), NEt_3_ (4eq )	4^d^

a Yield determined via calibrated Q-NMR with trichloroethylene (36 μL) as internal standard.

b Highly selective but lower reactivity.

c 24% side product **Id** formation (yield based on Katritzky salt).

d Higher cell potential was observed.

**Figure 3:**
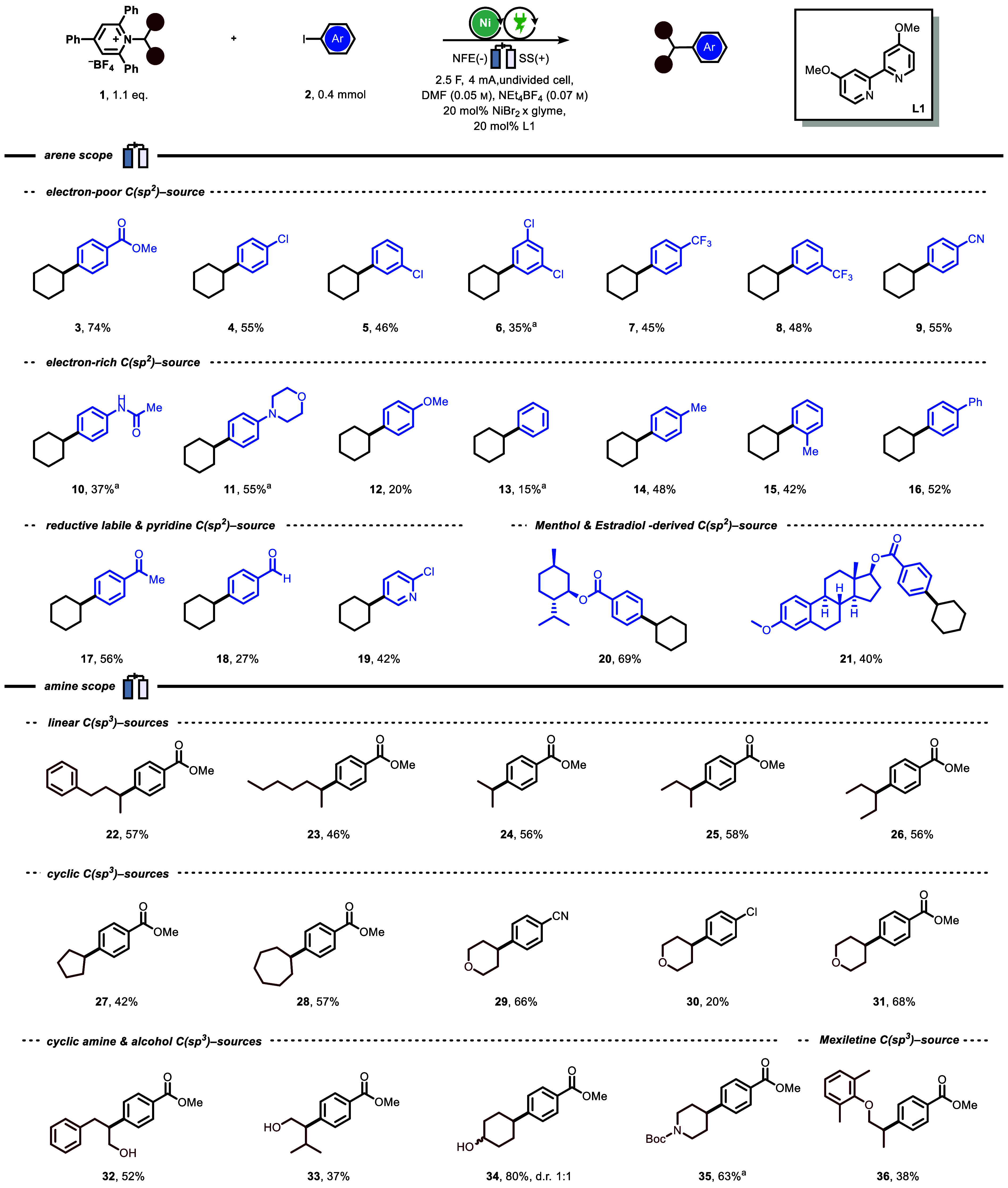
Scope of the nickel-catalyzed electrochemical reductive cross-electrophile coupling between Katritzky salts and aryl iodides. For further experimental details, see the SI. Yields refer to those obtained after isolation. ^a^Katritzky salt (2.0 equiv instead of 1.1 equiv), 20 mol % **L3** instead of **L1** (**L3** = 1,3-bis(2-pyridylimino)isoindoline = MeBPI), NEt_4_BF_4_ (0.025 m), *I* = 2 mA, A = 1 cm^2^, *J* = 2.0 mA/cm^2^, *Q* = 5.5 *F* = 212 C (0.4 mmol = Ar–I), *t* = 29.5 h.

Having established appropriate reaction conditions, we explored the scope of the nickel-catalyzed electrochemical reductive XEC of Katritzky salts and aryl iodides. Initially, we investigated a variety of aryl iodides using the cyclohexylamine-derived Katritzky salt as the benchmark coupling partner (Figure 3). Iodoarenes bearing electron-withdrawing substituents, such as chloro, trifluoromethyl, and cyano, as well as acetyl-protected aniline and morpholine-bearing arenes, were all efficiently coupled with cyclohexane, providing the desired products in synthetically valuable yields (**4**–**11**). Iodo-arenes featuring electron-neutral and -rich substituents, such as methoxy, methyl, and phenyl, also underwent cross-coupling, albeit with lower yields (**12**–**16**). Under these mild electrochemical conditions, iodoarenes carrying substituents that can be susceptible to reduction, including ketone and aldehyde groups, were also tolerated (**17**, **18**). Iodopyridines, exemplified by **19**, gave the desired product in 32% isolated yield. Furthermore, the cross-coupling of substrates derived from both menthol and estradiol with the cyclohexylamine-based Katritzky salt (**20**, **21**) was successfully achieved.

We then combined methyl 4-iodo benzoate with Katritzky salts derived from various secondary alkyl amines. We observed that the system displayed efficient performance, regardless of the length of the alkyl chain: 1-phenylbut-2-yl, 2-heptyl, isopropyl, *sec-*butyl, 3-pentyl amine derivatives all demonstrated similarly good reactivity (**22**–**26**). We were able to install cycloalkanes with ring sizes ranging from five to seven carbons at the para position of methyl benzoate (**27**, **28**). Likewise, we observed successful arylation of tetrahydropyran with various para-substituted arenes (**29**–**31**) using our electrochemical protocol. Notably, our protocol also demonstrated good tolerance toward nonprotected alcohols (**32**–**34**); specifically, we found that Katritzky salts derived from phenylalaninol, valinol, and 4-hydroxycyclohexylamine were all effective coupling partners. Excellent yield was obtained in the coupling between Boc-protected piperidine and iodo methyl benzoate (**35**). Furthermore, we demonstrated the synthetic value of our methodology by successfully coupling a medicinally relevant moiety, such as a mexiletine-derived Katritzky salt, under the electrochemical reaction conditions (**36**). To further investigate the applicability of our methodology, we attempted primary amine-derived Katritzky salts. Unfortunately, even after a thorough screening, including elevated reaction temperatures and modified alkylpyridinium salts, only traces of the desired product were observed (see SI, Table S2).

## Conclusions

In summary, our novel and versatile nickel-catalyzed electrochemical methodology offers a robust and efficient approach for forging C(sp^2^)–C(sp^3^) linkages via reductive cross-electrophile coupling. With the use of abundant and inexpensive amine-derived radical precursors and aryl iodides, this method offers modularity and allows for the synthesis of a diverse array of sp^3^-enriched cross-coupled products. Additionally, our methodology provides a sustainable alternative to traditional cross-coupling strategies, minimizing waste and reducing the need for chemical reductants. Overall, this nickel-catalyzed electrochemical methodology has the potential to significantly impact synthetic chemistry and contribute to the development of more sustainable and environmentally friendly synthesis methods.

## Data Availability

The data underlying this study are available in the published article and its Supporting Information.

## Supporting Information Available

Experimental procedures, characterization data, full detailed optimization, cyclic voltammetry data, and proposed mechanism (PDF)
FAIR data, including the primary NMR FID files, for compounds **3**–**36** (ZIP)
