# Supplementary material for: Ni-Catalyzed Electro-Reductive Cross-Electrophile Couplings of Alkyl Amine-Derived Radical Precursors with Aryl Iodides
Source: J Org Chem. 2023 May 23;89(22):16121–5. doi: 10.1021/acs.joc.3c00859 (PMC11574849; doi:10.1021/acs.joc.3c00859)

Supporting Information

Ni-catalyzed electro-reductive cross-electrophile couplings of alkyl amine-derived radical precursors with aryl iodides

Lars J. Wesenberg[a], Alessandra Sivo[b], Gianvito Vile[b], Timothy Noël[a]*

[a] *Van't Hoff Institute for Molecular Sciences (HIMS), University of Amsterdam (UvA), Amsterdam Science Park 904, 1098 XH, The Netherlands,*

[b] *Department of Chemistry, Materials, and Chemical Engineering "Giulio Natta", Politecnico di Milano, IT-20133 Milano, Italy*

** Email: t.noel@uva.nl*

Table of Contents

General Information 3

1. Experimental Procedures 4

   1.1 Preparation of pyridinium salts 4

   1.2 Coupling reaction 4

Upscaling of electrochemical reaction 25

2. References 26

3. NMR Spectra 25

General Information

All reagents and solvents were used as received without further purification, unless stated otherwise. Reagents and solvents were bought from Sigma Aldrich, TCI, Fluorochem, Fisher Scientific and, if applicable, kept under argon or nitrogen atmosphere. Technical solvents were bought from VWR International and Biosolve, and were used as received. All 2,4,6-triphenylpyridinium salts were prepared according to reported procedures.[1,2] 3-methoxy-17-estradiol-4-iodobenzoate[3] and menthol-4-iodobenzoate[4] were obtained using the corresponding reported literature procedure. The nickel catalyst was prepared according to a published procedure.[5] For reactions that required heating an oil bath was used. Disposable syringes were purchased from Laboratory Glass Specialist. Product isolation was performed manually, using silica (60, F254, Merck™), preparative TLC (silica gel GF, UV254, Uniplate™), and reversed-phase silica with automated system (C18, 12 gr. flash cartridges, spherical C18, 20-45 um with Biotage™ using acetonitrile/water mixtures as eluent systems with 0.1% formic acid). TLC analysis was performed using Silica on aluminum foils TLC plates (F254, Supelco Sigma-Aldrich™) with visualization under ultraviolet light (254 nm and 365 nm) or appropriate TLC staining (cerium molybdate or potassium permanganate). ^{1}H (300 MHz), ^{13}C (75 MHz) and ^{19}F (282 MHz) spectra were recorded at ambient temperature using Bruker AV 300-I. All ^{1}H NMR and ^{13}C NMR spectra are reported in parts per million (ppm) downfield relative to $CDCl_3$ (7.26 ppm and 77.16 ppm respectively). NMR multiplicities of signals are reported by the following abbreviations: s (singlet), d (doublet), t (triplet), br t (broad triplet), q (quartet), h (sextet), hept (septet), m (multiplet), br m (broad multiplet), dd (doublet of doublets), tt (triplet of triplets), td (triplet of doublets), qd (quartet of doublets), dtd (doublet of triplet of doublets). Coupling constants (J) are reported in hertz (Hz). High resolution mass spectra (HRMS) were obtained using an AccuTOF LC, JMS-T100LP Mass spectrometer (JEOL, Japan) or on an AccuTOF GC v 4g, JMS-T100GCV Mass spectrometer (JEOL, Japan). Cyclic voltammograms were recorded on a PGSTAT302N potentiostat (Autolab) with a working electrode (WE) = glassy carbon (MetrOhm, diameter 3 mm), reference electrode (RE) = Ag/AgCl (eDAQ, ET069) and counter electrode (CE) = platinum wire in 0.1 M tetraethylammonium tetrafluoroborate (TEATFB) in MeCN. The solvents prior to use were freeze-pump-thawed with argon as inert gas to suppress overlap of the oxygen reduction peak in cyclic voltammograms. The cyclic voltammogram experiment vessel was self-made from the glass blower and was operated under inert conditions. Prior to use the glass cell was put at 160 °C overnight in an oven. Constant current electrolysis for optimization and scope was carried out using multichannel power supply HMP4040 (Rohde & Schwarz, Munich, Germany) as power source. The electrochemical undivided cell was designed by our lab and the workshop (see picture below). The electrodes such as nickel-foam-electrode (NFE), stainless steel, and zinc are commercially available from IKA (IKA-Werke GmbH & Co. KG, Staufen, Germany). FAIR Data is available as Supporting Information for Publication and includes the primary NMR FID files for compounds [3-36]

1. Experimental Procedures

1.1 Preparation of pyridinium salts

General Procedure A1 - Preparation of pyridinium salts from amines

Following the literature procedure,[1] the amine (6 mmol, 1.2 equiv.) was added dropwise to a solution of 2,4,6-triphenylpyrylium tetrafluoroborate (1.98 g, 5.0 mmol, 1.0 equiv.) in EtOH (5 mL). The reaction mixture was refluxed at 90 °C for 5 h, then cooled to room temperature. The resulting precipitate, obtained after dilution with cold Et_2O (15 mL), was filtered, washed with cold Et_2O, and dried overnight under vacuum. If the precipitation failed, the reaction mixture was concentrated under vacuum and purified by column chromatography, using a mixture of acetone/CH_2Cl_2 as eluent.

General Procedure A2 - Preparation of pyridinium salts from ammonium salts (hydrochloride salt)

Following the literature procedure, Et_3N (2.0 equiv) was added to a suspension of ammonium salt (5 mmol, 1 equiv), 2,4,6-triphenylpyrylium tetrafluoroborate (1.98 g, 5.0 mmol, 1.0 equiv) and powdered activated 4Å molecular sieves (~500 mg/mmol) in CH_2Cl_2 (10 mL). The reaction mixture was stirred for 30 min at room temperature. Then, acetic acid (2.0 equiv) was added and the mixture was stirred overnight at room temperature. At the end of the reaction the suspension was filtered through a short pad of Celite and the resulting filtrate was washed with aq. HCl (1.0 M, 2 x 30 mL), aq. $NaHCO_3$ (sat. 2 x 30 mL), and brine (2 x 30 mL). The organic layer was dried over Na_2SO_4, and concentrated under reduced pressure. The resulting precipitate, obtained after dilution with cold Et_2O (15 mL), was filtered, washed with cold Et_2O, and dried overnight under vacuum. If the precipitation failed, the reaction mixture was concentrated under vacuum and purified by column chromatography, using a mixture of acetone/CH_2Cl_2 as eluent.

1.2 Coupling reaction

General Procedure B

A nitrogen-filled 10 mL batch-type undivided electrochemical cell was charged with aryliodide (0.40 mmol, 1 equiv, 0.05 M), Katritzky salt (0.44 mmol, 1.1 eq.), $NiBr_2$ x dme (25 mg, 0.08 mmol, 0.2 equiv), L1 (17 mg, 0.08 mmol, 0.2 equiv), NEt_4BF_4 (122 mg, 0.56 mmol, 0.07 M), DMF (8 mL), and a magnetic stirring bar. A nickel foam cathode and a sacrificial stainless-steel anode were inserted into the sealed test tube. Applying a constant current ($I = 4$ mA, $A = 2$ cm^2, $j = 0.5$ mA/cm^2, $Q = 2.5$ F = 96.5 C), the reaction mixture was stirred at room temperature and electrolyzed for 6.7 h (corresponds to 2.5 F). The crude mixture obtained was diluted with water (1 x 40 mL) and extracted with ethyl acetate (3 x 30 mL). The organic layers were combined and washed with brine (2 x 50 mL). The organic layer was dried over Na_2SO_4, and concentrated under reduced pressure. Column chromatography on silica gel afforded the corresponding products in the stated yield.

Cleaning Procedure for the Electrodes

After rinsing the nickel-foam and stainless steel electrodes with water, acetonitrile and acetone, all electrodes were sonicated in vessel/vial for 10 min containing acetonitrile. Afterwards the stainless steel was removed from the sonicator and thoroughly polished on each sides using a sandpaper explicitly made for metal-based materials (grain size: 240-400, also compare below used stainless steel electrodes vs fresh polished). Due to this procedure the stainless steel electrodes could be used for more than 15 reactions, before the corrosion of the sacrificial electrode made the electrochemical reductive cross-coupling unreliable. The nickel-foam electrodes were afterwards submerged into a vessel/vial containing 2 M HCl with some sacrificial aluminum foil, preventing the nickel electrode to degrade significantly and then sonicated for 10 min. Next, the diluted HCl and the aluminum foil were removed and the NFE was rinsed thoroughly with water and again submerged in a vessel/vial containing 2 M NaOH and sonicated for 10 min. This time without aluminum foil due to the unwanted formation of aluminum tetrahydroxide which could precipitate as white solid on the nickel NFE and influence the reactivity of the cathodic reaction. Afterwards the NFE was rinsed with water, acetonitrile, and acetone. Due to this procedure the NFE could be used many times without significant decrease in yield. After about 20 reactions the NFE electrode became a bit colored

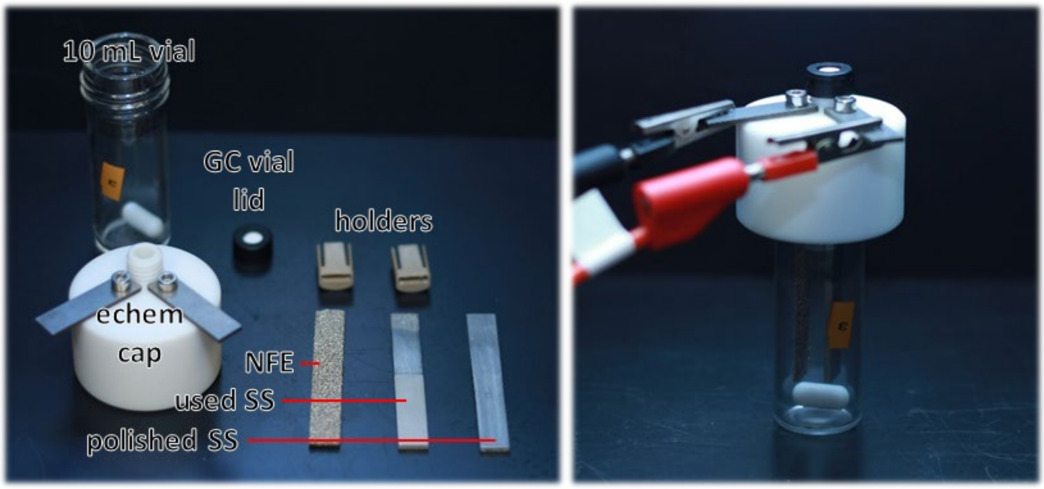

Figure S1: electrochemical batch set-up in 10 mL vial with NFE and stainless steel electrodes (SS).

Optimization for the electrochemical reductive coupling with Katritzky salts

Table S1: Full optimization table of the electrochemical XEC

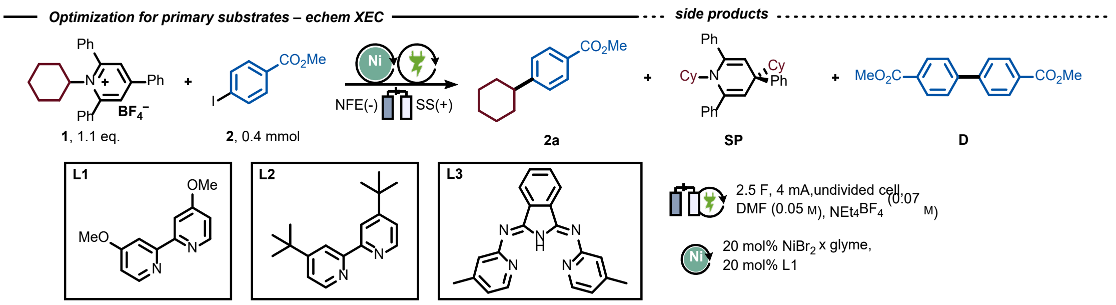

#	Deviation from above's conditions	Yield (%)[a] 2a	Yield (%)[a] RSM	Yield (%)[a*] Side Product	Yield (%)[a] Dimer
1	none	76	0	14	10
2	aryl bromide, 0.5 eq. NaI w/o TEABF4	23[b]	23	30	14
3	Zn (+) w/o electricity	41	23	30	02
4	SS (+) w/o electricity	00	100	00	00
5	L2 instead of L1	49	<5	07	20
6	L3 instead of L1	43	34	23	00
7	L3 instead of L1: 5.5 F, 2 mA	80	<3	24	02
8	impervious graphite (+), DIPEA (4 eq.)	10[c]	80	12	01
9	impervious graphite (+), NEt3 (4 eq.)	04[c]	83	10	00
10	DMA instead of DMF	30	33	05	30
11	MeCN instead of DMF	31[d]	15	15	17
12	DMSO instead of DMF	05	28	34	09
13	NMP instead of DMF	00[e]	93	<3	00
14	0 mol% Ni-cat.	00	83	40	00
15	10 mol% Ni-cat.	57	11	15	11
16	1.5 eq. of Katritzky salt	71	09	08	8
17	1.0 eq. Katritzky salt; 1.5 eq. Ar–I	50	26	04	28
18	2 mA instead of 4 mA	71	00	03	12

a: Yield determined via Q-NMR with trichloroethylene (36µL) as standard; a*: Yield determined via Q-NMR with trichloroethylene as standard with respect to the Katritzky salt; b: no significant voltage increase observed; c: high cell potential detected; d: insoluble issues; e: no corrosion of SS and no reactivity of KS observed

Preliminary results were obtained containing aryl iodide **2**, Katritzky salt **1**, tetrabutylammonium tetrafluoroborate (TEABF4), NiBr2 x glyme as precatalyst, 4,4'-di-tert.-butyl-2,2'-bipyridine (**L2**) as ligand, and DMF as solvent. For the electrochemical setup, we have chosen an undivided batch-cell arrangement with zinc as sacrificial anode and nickel foam electrode (NFE) as cathode material – commonly used in electro-reductive procedures. Representative results are shown in Table 1. The use of aryl bromides with sodium iodide as additive did not operate well. So the use of aryl iodides could not be avoided (Table S1, entry 2).

The use of zinc as anode material revealed no need for electricity (Table S1, entry 3). By comparing the standard reduction potentials of the model substrate $E°$ (**1**/**1**$^{\bullet-}$) = -0.54 V *vs.* SHE[1,6] and zinc $E°$ (Zn^{2+}/Zn^0) = -0.76 V *vs.* SHE[7] it can be quickly calculated that the reaction can occur spontaneously: $E_{cell} = E_{cathode} - E_{anode}$ = -0.54 V + 0.76 V = +0.22 V. Alternatively, changing the anode material to a different metal such as stainless steel, may be beneficial due to abundancy of iron in the earth crust[7] and a suitable standard reduction potential: $E°$ (Fe^{2+}/Fe^0) = -0.44 V vs. SHE $E_{cell} = E_{cathode} - E_{anode}$ = -0.54 V + 0.44 V = -0.10 V. The overall cell potential shifts to negative values. Hence, an electrochemical approach becomes feasible. With stainless steel as electrode material and running the reaction without electricity revealed no conversion of starting material (Table S1, entry 4).

The bidentate ligand **L2** is commonly used in nickel catalysis. Nevertheless, yet much lower selectivity was obtained with dimerization in 18% yield and the desired product **2a** in moderate yield of 49% respectively (Table S1, entry 5). Next, **L3** was subjected as ligand in our system without the usage of a shuttle like Sevov et al. recently published.[8] Notably, lower reactivity was observed but higher selectivity towards aryl-alkyl coupling. Homo coupling could be suppressed entirely. Hence applying higher amount of charge (Table S1, entry 7, 5.5 F = 29.5 h reaction time) could improve the product formation slightly up to 80% but with much lower productivity. Furthermore, the formation of a new side product **Id** was observed substantially with 24% yield.

We continued our investigation by examining the counter reaction. It has already been proven above that the control of the theoretical cell potential is pivotal in this endeavor. We compared the practicability of various terminal reductants such as diisopropylethylamine (DIPEA), and triethylamine (TEA) with sacrificial anodes such as stainless steel. Unfortunately, the reaction did not operate properly by the use of tertiary amines (Table S1, entry 8, entry 9). Comparing the generic peak cathodic potentials of tertiary amines approximately $E^{pc} \approx 0.9$ V with the standard reduction potential $E°$ (Fe^{2+}/Fe^{0}) = -0.44 V it is not surprising that the observed overall cell potential during the reaction was substantially higher (E_{cell} (amines) ≈ 1.7 V *vs.* E_{cell} (SS) ≈ 0.95 V). Thus, undesired side reaction may occur and the decrease in yield can be rationalized. Then, we examined the solvent system (Table S1, entries 10-14). In literature it has been thoroughly studied that nickel-catalyzed XEC reactions need polar solvents such as DMF, DMA, NMP, or rarely used DMSO[9,10] and acetonitrile.[11] Neither DMSO nor MeCN could improve the outcome of the reaction towards the cross-coupling product due to either very high cell potential (E_{cell} = 2.2 V with DMSO) or solubility issues (with acetonitrile). Surprisingly, using NMP as solvent the reactivity dropped dramatically. No corrosion of the stainless steel was observed, and thus no reduction of Katritzky salt **1** was detected. Unusually, applying DMA resulted in aryl-aryl coupling with 33% as major and aryl-alkyl coupling **3** with 30% as minor product. Hence, DMF was chosen for the optimal solvent system.

Next, using a lower catalyst loading of only 10 mol% (Table S1,entry 15) resulted also in lower yield of 57%. A control experiment where no catalyst was used revealed no reactivity, which was expected (Table S1, entry 14). Furthermore, applying no current with the optimized conditions in hand also did not proceed in product formation or any conversion of the starting materials.

In the end, stoichiometry experiments and the current density was investigated, but no improvement was observed with inversed stoichiometry or lower current density (Table S1, entries 16-18).

Unsuccessful Katritzky salts

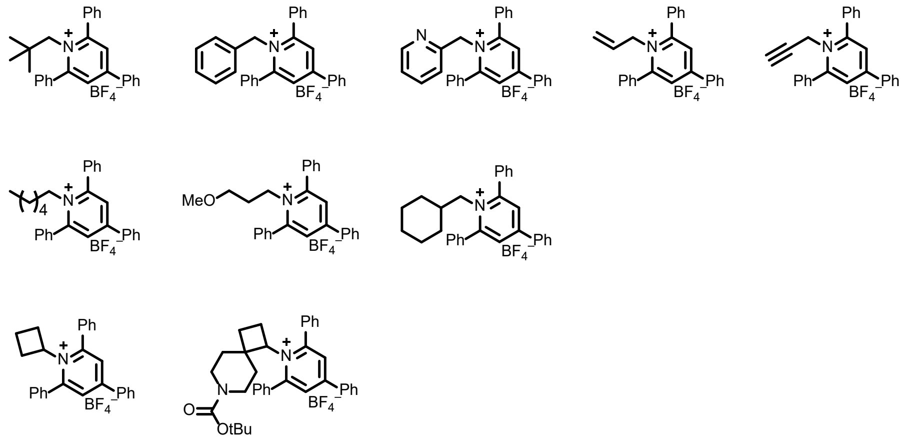

Optimizing for primary Katritzky-salts

Table S2: Screening of various conditions for the activation of primary amine-derived Katritzky salts.

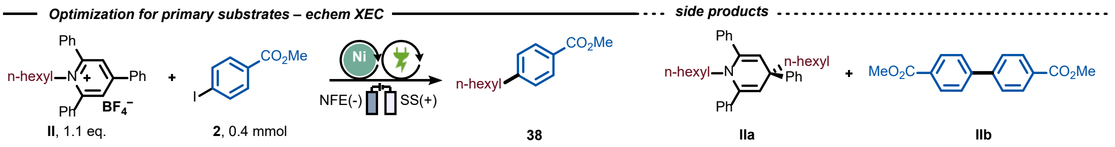

#	Deviation from optimized conditions	Yield (SM) / (%)[a]	Yield (38) / (%)[a]	Yield (IIa) / (%)[a]	Yield (IIb) / (%)[a]
1	50 °C, 4 mA	<5	3	14	39
2	25 °C, 6 mA	<5	1	09	49
3	50 °C, 6 mA	<5	4	22	47
4	25 °C, 8 mA	<5	0	00	46
5	50 °C, 8 mA	<5	3	20	50
6	50 °C, 6 mA	<5	4	18	44
7	50 °C, 6 mA	<5	4	08	30
8	50 °C, 6 mA	07	7	00	09
9	50 °C, 12 mA	<5	3	18	43
10	50 °C, 12 mA	<5	5	06	27
11[b]	25 °C, 4 mA, Katritzky-salt **37**	09	0	00	45

a: Q-NMR with internal standard 1,1,2-trichloroethylene; b: no reactivity at all, KS was still intact and didn't show reduction & cleavage

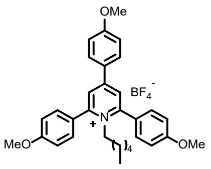

37

Cyclic Voltammetry

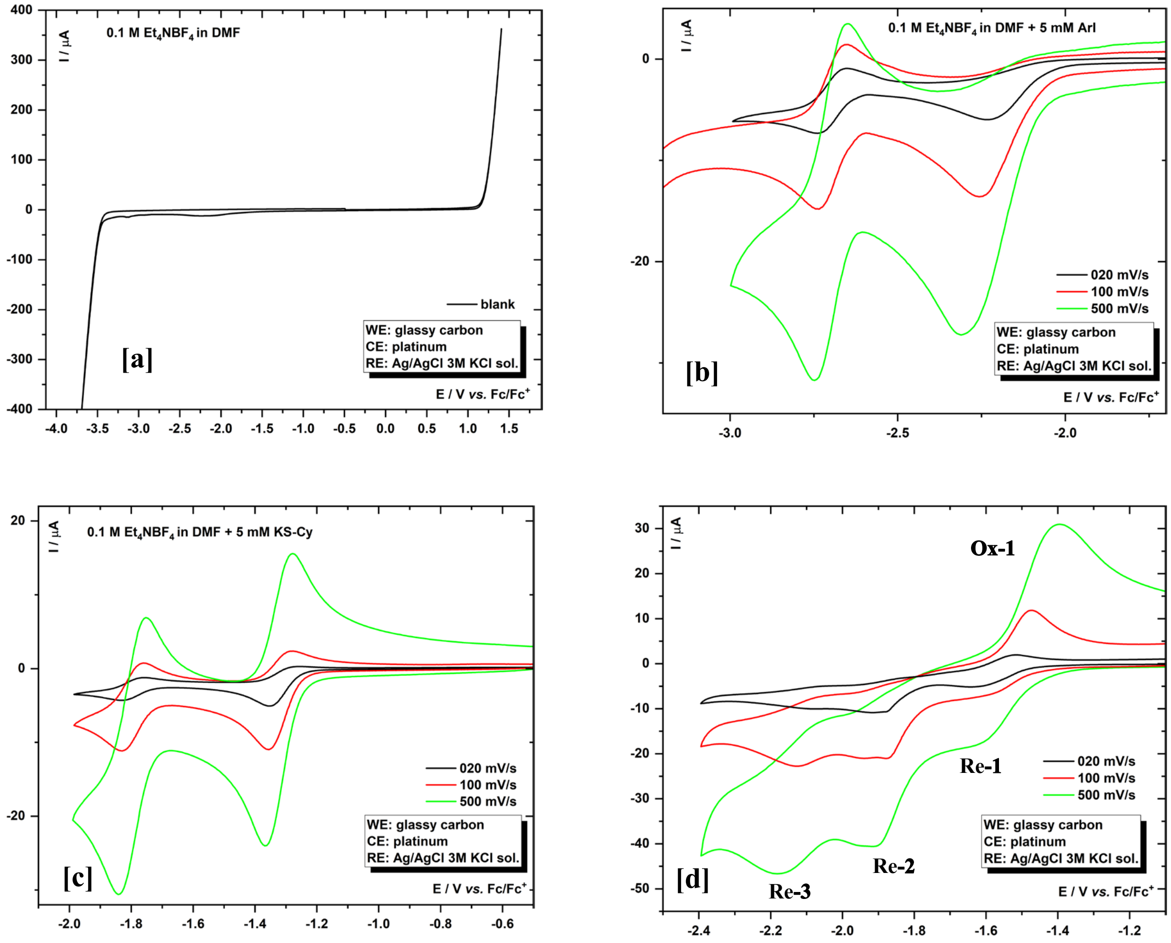

Figure S2: *[a] blank cyclic voltammetry with only supporting electrolyte tetraethyl ammonium tetrafluoroborate (Et_4NBF_4) in DMF (0.1 M), scan rate = 100 mV/s. [b] 5 mM Ar–I in 0.1 M Et_4NBF_4/DMF, scan rates from 20 mV/s (black), 100 mV/s (red) to 500 mV/s (green). [c] 5 mM Katritzky salt with Cyclohexyl substituent in 0.1 M Et_4NBF_4/DMF, scan rates from 20 mV/s (black), 100 mV/s (red) to 500 mV/s (green). [d] 5 mM Ni-cat in 0.1 M Et_4NBF_4/DMF, scan rates from 20 mV/s (black), 100 mV/s (red) to 500 mV/s (green).* Working electrode (WE): glassy carbon, counter electrode (CE) : platinum wire, reference electrode (RE): Ag/AgCl 3 M KCl solution. Working temperature: 25 °C; scan starting point: 0.0 V, direction of scan: towards negative potentials.

We have carried out CV experiments to gain mainly information about the role of the Ni-catalyst. We were curious if the catalyst can function (1) as reducing agent for the Katritzky salt and (2) which species Ni(I) or Ni(0) is responsible for the oxidative addition of the aryl iodide. If an increase in cathodic current (I_{pc}) is observed after titration of a potential reactant to the nickel catalyst, this is referred to as a catalytic current. This means that the reduced nickel catalyst [Ni(I) or Ni(0)] is reacting with your titrated reagent in the CV vessel.

Some remarks: Since the cyclic voltammograms were recorded with glassy carbon as the working electrode (which is most commonly used in CV experiments) instead of a nickel surface (which was the actual electrode used in the scope), we cannot exclude the possibility of a thermodynamically favored interaction between homogeneous Ni-complex and heterogenous Ni-electrode. This interaction may lead to less negative potentials of the Ni-cat reduction. The titration was attempted firstly with the Katritzky salt **1** in combination with the nickel-catalyst due to overlapping signals it was not further pursued. The CV experiment can be seen as in idealistic system which does not represent reality. In a typical set-up compensation for ohmic drop is desired by using excessive amounts of supporting electrolyte which

means 1:20 ratios at least between analyte and supporting electrolyte. In the electrochemical XEC in the batch cell the ratio was nearly 1:1. All this assumptions and remarks can have major impacts on the reactivity and the reduction of nickel catalysts and reactants and have to take into account.

[Fig. S2b] On the top right you can see two distinguishable reduction waves for the test substrate methyl 4-iodobenzoate **2**, where the first wave is irreversible and the second is fairly reversible with a peak seperation of 100 mV. First reduction peak occurs at $E^{I}_{p/2}$(**2**/**2**$^{•-}$) = –2.14 V *vs.* Fc/Fc$^+$ and second at $E^{II}_{1/2}$(**2**/**2**$^{•-}$) = –2.78 V *vs.* Fc/Fc$^+$. Most likely the aryl iodide is being reduced to the radical anion and followed by an mesolytical cleavage of the carbon–halogen bond. The corresponding intermediate can then easily undergo another reduction. Since there is no labile leaving group attached it is quasi-reversible under the applied conditions.

[Fig. S2c] Next, based on the results shown above we can surely state that the first reduction wave of the Katritzky salt **1** occurs at $E^{I}_{1/2}$(**1**/**1**$^{•-}$) = –1.33 V *vs.* Fc/Fc$^+$, second occurs at $E^{II}_{1/2}$(**1**/**1**$^{-2}$) = –1.80 V *vs.* Fc/Fc$^+$. Both waves can be considered fairly as reversible peaks with a peak seperation of 80 mV for the first and 90 mV for the second peak. After the first reduction of the Katritzky salt **1** the generated radical is a persistent radical. Additional data from the optimization above has confirmed that a side reaction in the electrochemical transformation was detected (Figure S3). This can only occur if one of the radicals involved is persistent radical **Ia**.[12,13] The accumulation of the side product **Id** was an unproductive pathway. The cleavage of those side products **Id** could only be initiated under oxidative conditions.[14] Since our reaction was performed with a stainless steel sacrificial electrode which is much more favorable to be oxidized the side product coming from the persitent radical could be considered a dead end.

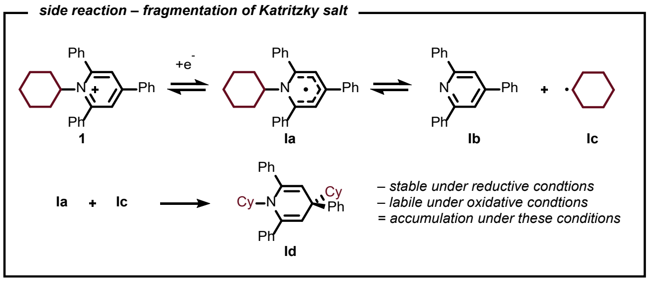

Figure S3: Formation of side product.[15]

Nevertheless, the desired C(sp^2)–C(sp^3) coupling was succesfully achieved, and it is plausible that the cleavage of the persistent radical **Ia** takes place in presence of coupling partners such as nickel catalyst and/or aryl iodide. However no cleavage was detected in the CV enviroment without other reagents in the mixture (reversibility was preserved). Thus we can state that the catalyst plays a crucial role to initiate the cleavage reaction of the reduced Katritzky salt **Ia**.

[Fig S2d] Here we measured the reduction behaviour of the nickel-catalyst at different scan rates. We assumed that the first reduction wave **Re-1** at $E_{p/2}$ = –1.52 V and the oxidation peak **Ox-1** at $E_{p/2}$ = –1.49 V can be seen as quasi-reversible (peak seperation shifted with different scan rates, indicating a quasi-reversibility). The peak current seperation is about > 200 mV (at scan rate: 500 mV/s). Nevertheless, checking the peak height I_{pc} = 16.8 µA (**Re-1** peak cathodic current) and I_{pa} = 29.3 µA (peak anodic current) it became clear to us that a different species must has formed. Additionally, this **Ox-1** peak was only observed after a full scan to –2.5 V *vs.* Fc/Fc$^+$. R. Martin and before Klein and co-workers suggested that this oxidation peak **Ox-1** is related to a solvated Ni(I) species formed *in situ* after reduction and bromide loss. These so-called EC-mechanism with cleavage of bromide are very common and has been observed for a number of diimine complexes of transition metals with halide co-ligands.[1,16,17] From these data, a weak reducing ability of the Ni catalyst was concluded, which may explain that primary Katritzky salts could not be reduced by this species under the given conditions.

However, elevated potentials at the cathode and/or temperatures resulted in the reduction of primary derivatives (see page S8). Nevertheless, the persistent pyridyl radical generated needs to dissociate which kinetically may not coincide with the nickel cycle under these tailored conditions. Only side product **IIa** (n-hexyl) was generated. The second peak **Re-2** at $E_{p/2} = -1.82$ V *vs.* Fc/Fc$^+$ is most likely related to the redox couple Ni(I)/Ni(0). Since nickel species with various oxidation states can undergo a plethora of reaction pathways such as dimerization and comproportionation, additional interpretation of follow-up peaks will be pure speculation.[18–20]

Titration of Ar-I into a solution containing nickel-catalyst

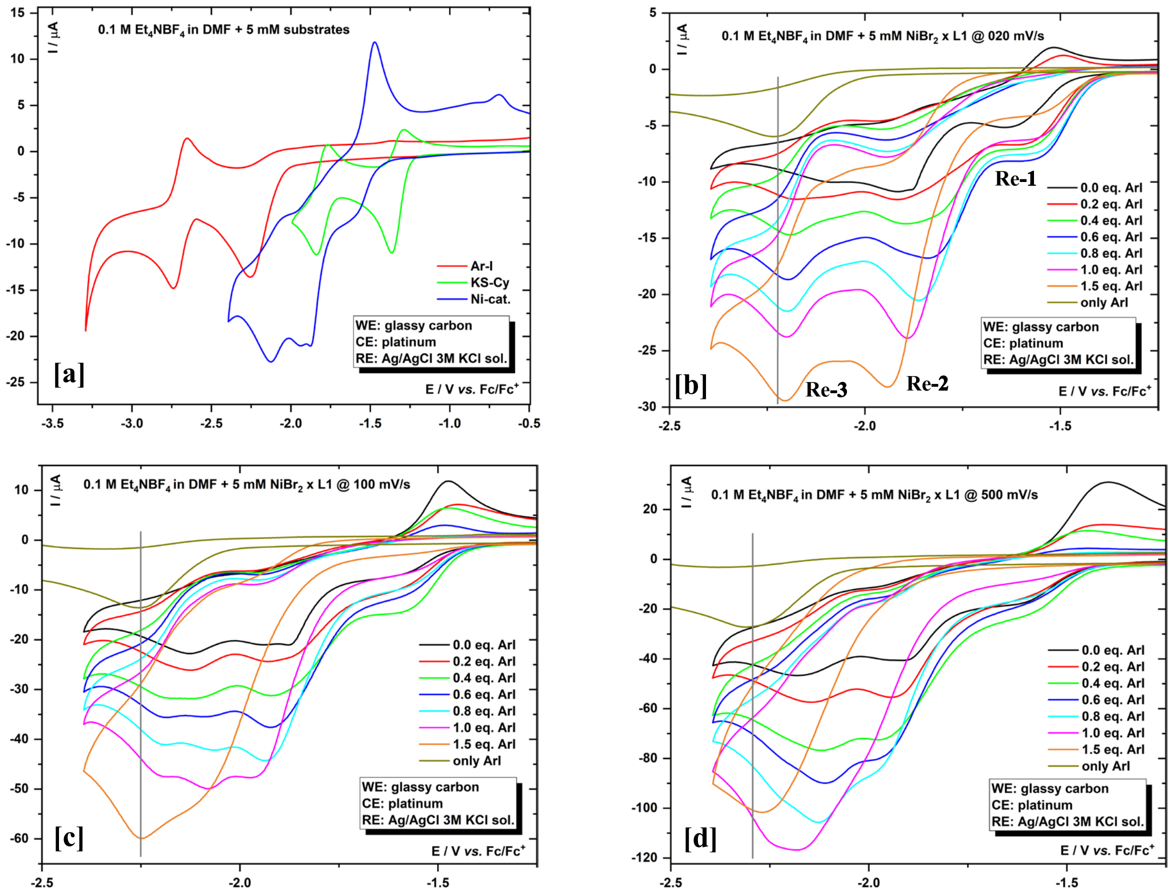

Figure S4: [a] superimposition of all compounds with supporting electrolyte tetraethyl ammonium tetrafluoroborate (Et_4NBF_4) in DMF (0.1 M), scan rate = 100 mV/s. [b] Titration of Ar–I in 5 mM Ni-cat with 0.1 M Et_4NBF_4/DMF, scan rate 20 mV/s; [c] Titration of Ar–I in 5 mM Ni-cat with 0.1 M Et_4NBF_4/DMF, scan rate 100 mV/s mV/s (green). [d] Titration of Ar–I in 5 mM Ni-cat with 0.1 M Et_4NBF_4/DMF, scan rate 500 mV/s. Working electrode (WE): glassy carbon, counter electrode (CE) : platinum wire, reference electrode (RE): Ag/AgCl 3 M KCl solution. Working temperature: 25 °C; scan starting point: 0.0 V, direction of scan: towards negative potentials.

[Fig. S4a] Superimposition of all compounds in one graph clearly shows (1) reduction of Katritzky salt **1** happens – under the given conditions – at slightly less negative potential than the potential needed to reduce Ni(II) to Ni(I) to Ni(I). Consequently, as soon as nickel catalyst is reduced at slighlty more negative potentials the resulting Ni(I) species will be able to reduce Katritzky salt **1** – again assuming no stabilizing or destabilizing interaction betwenn nickel electrode and substrate. (2) Reduction of aryl iodine **2** is detected at more negative potentials than the nickel catalyst. Under the operating conditions pure reduction of the aryl component towards hydrodehalogenation should not occur or at least only as a minor pathway. Indeed hydrodehalogenation was only observed in traces. The nickel catalyst functions as mediator for the oxidative addtion of aryl iodide **2**.

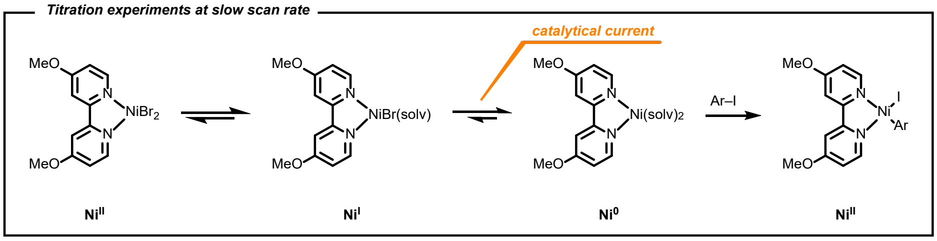

[Fig. S4b + S4c + S4d] Next, we investigated the behavior of the putative nickel species (Ni(I) or Ni(0)) responsible for the oxidative addition of the Ar–I substrate. We started our investigation by performing a cyclic voltammetry experiment with just nickel catalyst and no additives at different scan rates ranging from 20 mV/s, 100 mV/s, to 500 mV/s. Afterwards a titration experiment with the aryl component **2** was performed to see which reduction wave would undergo a catalytical current and which species would perform the oxidative addition. A significant catalytical current was observed at the second reduction wave **Re-2**. At slow scan rates of 20 mV/s, the catalytical current response is mainly located on the second reduction wave **Re-2** (Figure S4b). From a kinetic point of view, it makes sense that slow scan rates are favorable for these experiments to monitor the desired reaction of intermediate Ni(0) species with aryl substrate **2**. At faster scan rates, the catalytical current response appears to shift slightly towards reduction wave **Re-3** at E_{pc} = –2.10 V *vs.* Fc/Fc$^+$ (Figure S4c, S4d). Presumably, (1) any *in situ* generated nickel species (comproportionation, dimerization) in proximity to the electrode surface can be responsible for the oxidative addition. (2) Poisoning or saturation of the electrochemical double-layer and/or the electrode surface itself due the continuous addition of more aryl species obscures the redox response of nickel. (3) Last but not least significant amounts of arylated nickel species has formed during the course of the titration. Hence, resulting in a complex mixture of various nickel species. Especially, after the addition of 1.5 eq. of aryl iodide **2** the entire nickel curves are gone and only the first reduction wave $E_{p/2}$(**2**/**2**$^{•-}$) = –2.14 V *vs.* Fc/Fc$^+$ could be detected.

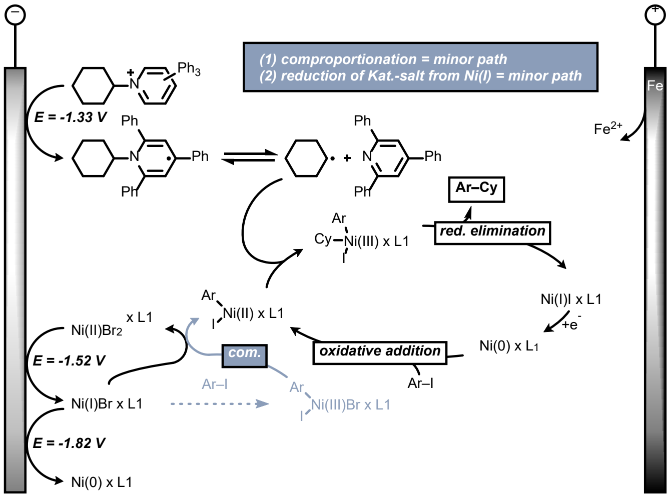

Figure S5: Proposed reaction mechanism of nickel-catalyzed XEC with Katritzky salts.

Characterization Data of Products

Arene scope

Methyl 4-cyclohexylbenzoate (3)

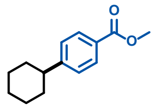

Prepared according to general procedure B, using methyl 4-iodobenzoate (104.8 mg, 0.4 mmol) and 2,4,6-triphenylpyrylium tetrafluoroborate (209.9 mg, 0.44 mmol). The reaction mixture was purified by column chromatography (pentane/toluene = 4/6) affording product **3** (64.6 mg, 74% yield) as a colorless oil. The spectroscopic data are consistent with those reported previously.[21]

^{1}H NMR (300 MHz, $CDCl_3$) δ 7.97 – 7.93 (m, 2H), 7.27 (d, J = 8.1 Hz, 2H), 3.90 (s, 3H), 2.62 – 2.48 (m, 1H), 1.92 – 1.82 (m, 4H), 1.78 – 1.74 (m, 1H), 1.50 – 1.30 (m, 4H), 1.28 – 1.23 (m, 1H).

^{13}C{1H} NMR (75 MHz, $CDCl_3$) δ 167.8, 154.0, 130.3, 128.3, 127.4, 52.5, 45.3, 34.7, 27.3, 26.6.

HRMS (FI) m/z: $[M]^+$ calculated for $C_{14}H_{18}O_2$: 218.1307, found: 218.1306.

1-Cyclohexyl-4-chlorobenzene (4)

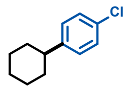

Prepared according to general procedure B, using 1-iodo-4-chlorobenzene (95.4 mg, 0.4 mmol) and 2,4,6-triphenylpyrylium tetrafluoroborate (209.9 mg, 0.44 mmol). The reaction mixture was purified by column chromatography (using pentane as eluent) affording product **4** (43 mg, 55% yield) as a colorless oil. The spectroscopic data are consistent with those reported previously.[22]

^{1}H NMR (300 MHz, $CDCl_3$) δ 7.30 – 7.26 (m, 2H), 7.19 – 7.14 (m, 2H), 2.56 – 2.47 (m, 1H), 1.91 – 1.77 (m, 5H), 1.46 – 1.25 (m, 5H).

^{13}C{1H} NMR (75 MHz, $CDCl_3$) δ 146.6, 131.4, 128.5, 128.3, 44.1, 34.6, 26.9, 26.2.

HRMS (FI) m/z: $[M]^+$ calculated for $C_{12}H_{15}Cl$: 194.0862, found: 194.0871.

1-Cyclohexyl-3-chlorobenzene (5)

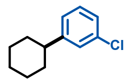

Prepared according to general procedure B, using 1-iodo-3-chlorobenzene (95.4 mg, 0.4 mmol) and 2,4,6-triphenylpyrylium tetrafluoroborate (209.9 mg, 0.44 mmol). The reaction mixture was purified by column chromatography (using pentane as eluent) affording product **5** (35.7 mg, 46% yield) as a colorless oil. The spectroscopic data are consistent with those reported previously.[23]

^{1}H NMR (300 MHz, $CDCl_3$) δ 7.24 – 7.16 (m, 3H), 7.11 – 7.06 (m, 1H), 2.53 – 2.44 (m, 1H), 1.87 – 1.69 (m, 5H), 1.46 – 1.30 (m, 5H).

^{13}C{1H} NMR (75 MHz, $CDCl_3$) δ 150.3, 134.2, 129.7, 127.2, 126.0, 125.2, 44.5, 34.4, 26.9, 26.2.

HRMS (FI) m/z: $[M]^+$ calculated for $C_{12}H_{15}Cl$: 194.0862, found: 194.0858.

1,3-Dichloro-5-cyclohexylbenzene (6)

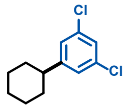

Prepared according to general procedure B, using 1,3-dichloro-5-iodobenzene (109.2 mg, 0.4 mmol) and 2,4,6-triphenylpyrylium tetrafluoroborate (209.9 mg, 0.44 mmol). The reaction mixture was purified by column chromatography (using pentane as eluent) affording product **6** (31.9 mg, 35% yield) as a colorless oil.

^{1}H NMR (300 MHz, $CDCl_3$) δ 7.17 (t, J = 1.9 Hz, 1H), 7.08 (d, J = 1.9, 2H), 2.54 – 2.38 (m, 1H), 1.90 – 1.69 (m, 5H), 1.48 – 1.17 (m, 5H).

^{13}C{1H} NMR (75 MHz, $CDCl_3$) δ 151.5, 134.8, 126.1, 125.7, 44.4, 34.2, 26.8, 26.1.

HRMS (EI) m/z: $[M]^+$ calculated for $C_{12}H_{14}Cl_2$: 228.0473, found: 228.0471.

1-Cyclohexyl-4-(trifluoromethyl)benzene (7)

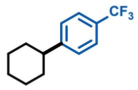

Prepared according to general procedure B, using 1-iodo-4-(trifluoromethyl)benzene (108.8 mg, 0.4 mmol) and 2,4,6-triphenylpyrylium tetrafluoroborate (209.9 mg, 0.44 mmol). The reaction mixture was purified by column chromatography (using pentane as eluent) affording product **7** (41.0 mg, 45% yield) as a colorless oil. The spectroscopic data are consistent with those reported previously.[1]

^{1}H NMR (300 MHz, $CDCl_3$) δ 7.53 (d, J = 8.1 Hz, 2H), 7.31 (d, J = 8.1 Hz, 2H), 2.62 – 2.49 (m, 1H), 2.00 – 1.72 (m, 5H), 1.49 – 1.24 (m, 5H).

^{13}C{1H} NMR (75 MHz, $CDCl_3$) δ 152.2, 128.2 (q, J = 32.4 Hz), 127.3, 126.6, 126.3, 125.4 (q, J = 3.8 Hz), 124.5 (q, J = 271.7 Hz), 122.8, 44.7, 34.4, 26.9, 26.2.

^{19}F NMR (282 MHz, $CDCl_3$) δ -62.25.

HRMS (FI) m/z: $[M]^+$ calculated for $C_{13}H_{15}F_3$: 228.1126, found: 228.1126.

1-Cyclohexyl-3-(trifluoromethyl)benzene (8)

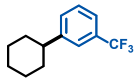

Prepared according to general procedure B, using 1-iodo-3-(trifluoromethyl)benzene (108.8 mg, 0.4 mmol) and 2,4,6-triphenylpyrylium tetrafluoroborate (209.9 mg, 0.44 mmol). The reaction mixture was purified by column chromatography (using pentane as eluent) affording product **8** (43.8 mg, 48% yield) as a colorless oil. The spectroscopic data are consistent with those reported previously.[24]

^{1}H NMR (300 MHz, $CDCl_3$) δ 7.50 – 7.33 (m, 4H), 2.66 – 2.47 (m, 1H), 1.99 – 1.82 (m, 4H), 1.82 – 1.74 (m, 1H), 1.49 – 1.38 (m, 4H), 1.36 – 1.25 (m, 1H).

^{13}C{1H} NMR (75 MHz, $CDCl_3$) δ 149.0, 130.9 (q, J = 32.0 Hz), 130.4, 128.8, 124.5 (q, J = 272.0 Hz), 123.7 (q, J = 3.9 Hz), 122.8 (q, J = 3.9 Hz), 44.6, 34.4, 26.9, 26.2.

^{19}F NMR (282 MHz, $CDCl_3$) δ -62.48.

HRMS (FI) m/z: $[M]^+$ calculated for $C_{13}H_{15}F_3$: 228.1126, found: 228.1137.

4-Cyclohexylbenzonitrile (9)

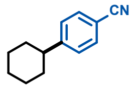

Prepared according to general procedure B1, using 1-iodo-4-benzonitrile (91.6 mg, 0.4 mmol) and 2,4,6-triphenylpyrylium tetrafluoroborate (209.9 mg, 0.44 mmol). The reaction mixture was purified by column chromatography (gradient elution, pentane/ethyl acetate = 1/0 to pentane/ethyl acetate = 9/1) affording product **9** (41 mg, 55% yield) as a colorless oil. The spectroscopic data are consistent with those reported previously.[25]

^{1}H NMR (300 MHz, $CDCl_3$) δ 7.57 (d, J = 8.3 Hz, 2H), 7.30 (d, J = 8.3 Hz, 2H), 2.60 – 2.51 (m, 1H), 1.91 – 1.80 (m, 5H), 1.44 – 1.24 (m, 5H).

^{13}C{1H} NMR (75 MHz, $CDCl_3$) δ 153.5, 132.2, 127.7, 119.2, 109.6, 44.8, 34.0, 26.6, 25.9.

HRMS (FI) m/z: $[M]^+$ calculated for $C_{13}H_{15}N$: 185.1204, found: 185.1198.

N-(4-cyclohexylphenyl)acetamide (10)

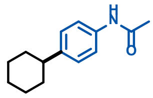

Prepared according to general procedure B, using N-(4-iodophenyl)acetamide (104.4 mg, 0.4 mmol) and 2,4,6-triphenylpyrylium tetrafluoroborate (209.9 mg, 0.44 mmol). The reaction mixture was purified first by normal-phase silica column chromatography (gradient elution, pentane/ethyl acetate = 1/0 to pentane/ethyl acetate = 95/5) and second by reversed-phase silica column chromatography (acetonitrile/water = 60/4 with 0.1% formic acid) affording product **10** (32.1 mg, 37% yield) as a colorless oil. The spectroscopic data are consistent with those reported previously.[26]

^{1}H NMR (300 MHz, $CDCl_3$) δ 7.39 (d, J = 8.5 Hz, 2H), 7.15 (d, J = 8.5 Hz, 2H), 2.55 – 2.38 (m, 1H), 2.15 (s, 3H), 1.85 – 1.71 (m, 5H), 1.42 – 1.26 (m, 5H).

^{13}C{1H} NMR (75 MHz, $CDCl_3$) δ 168.4, 144.4, 135.7, 127.4, 120.3, 44.1, 34.6, 27.0, 26.2, 24.6.

HRMS (EI) m/z: $[M]^+$ calculated for $C_{14}H_{19}NO$: 217.1467, found: 217.1474.

4-(4-Cyclohexylphenyl)morpholine (11)

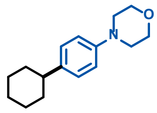

Prepared according to general procedure B, using 4-(4-iodophenyl)morpholine (115.6 mg, 0.4 mmol) and 2,4,6-triphenylpyrylium tetrafluoroborate (209.9 mg, 0.44 mmol). The reaction mixture was purified first by normal-phase silica column chromatography (gradient elution, pentane/ethyl acetate = 1/0 to pentane/ethyl acetate = 9/1) and second by reversed-phase silica column chromatography (acetonitrile/water = 60/40 with 0.1% formic acid) affording product **11** (53.9 mg, 55% yield) as a colorless oil. The spectroscopic data are consistent with those reported previously.[1]

^{1}H NMR (300 MHz, $CDCl_3$) δ 7.16 – 7.10 (m, 2H), 6.89 – 6.83 (m, 2H), 3.86 (dd, J = 4.2, 2.5 Hz, 4H), 3.13 (dd, J = 4.2, 2.5 Hz, 4H), 2.50 – 2.34 (m, 1H), 1.91 – 1.67 (m, 5H), 1.44 – 1.30 (m, 5H).

^{13}C{1H} NMR (75 MHz, $CDCl_3$) δ 149.5, 140.1, 127.6, 115.9, 67.2, 49.8, 43.8, 34.7, 27.1, 26.3.

HRMS (EI) m/z: $[M]^+$ calculated for $C_{16}H_{23}NO$: 245.1780, found: 245.1784.

1-Cyclohexyl-4-methoxybenzene (12)

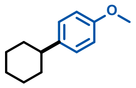

Prepared according to general procedure B, using 1-iodo-4-methoxybenzene (93.6 mg, 0.4 mmol) and 2,4,6-triphenylpyrylium tetrafluoroborate (209.9 mg, 0.44 mmol). The reaction mixture was purified by column chromatography (using pentane as eluent) affording product **12** (15.2 mg, 20% yield) as a colorless oil. The spectroscopic data are consistent with those reported previously.[1]

^{1}H NMR (300 MHz, $CDCl_3$) δ 7.14 (d, J = 8.6 Hz, 2H), 6.85 (d, J = 8.6 Hz, 2H), 3.79 (s, 3H), 2.51 – 2.39 (m, 1H), 1.91 – 1.71 (m, 5H), 1.44 – 1.26 (m, 5H).

^{13}C{1H} NMR (75 MHz, $CDCl_3$) δ 157.8, 140.6, 127.8, 113.8, 55.4, 43.9, 34.9, 27.1, 26.4.

HRMS (EI) m/z: $[M]^+$ calculated for $C_{13}H_{18}O$: 190.1358, found: 190.1367.

Cyclohexylbenzene (13)

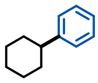

Prepared according to general procedure B using iodobenzene (81.6 mg, 0.4 mmol) and 2,4,6-triphenylpyrylium tetrafluoroborate (209.9 mg, 0.44 mmol). The reaction mixture was purified by column chromatography (using pentane as eluent) affording product **13** (9.6 mg, 15% yield) as a slightly brown oil. The spectroscopic data are consistent with those reported previously.[27]

^{1}H NMR (300 MHz, $CDCl_3$) δ 77.36 – 7.29 (m, 2H), 7.28 – 7.20 (m, 3H), 2.58 – 2.49 (m, 1H), 1.95 – 1.85 (m, 4H), 1.79 (dd, J = 13.4, 1.6 Hz, 1H), 1.50 – 1.40 (m, 4H), 1.38 – 1.28 (m, 1H).

^{13}C NMR (75 MHz, $CDCl_3$) δ 148.3, 128.4, 127.0, 125.9, 44.8, 34.6, 27.1, 26.4.

HRMS (FI) m/z: $[M]^+$ calculated for $C_{12}H_{16}$: 160.1252, found: 160.1250.

1-Cyclohexyl-4-methylbenzene (14)

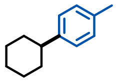

Prepared according to general procedure B, using 1-iodo-4-methylbenzene (87.2 mg, 0.4 mmol) and 2,4,6-triphenylpyrylium tetrafluoroborate (209.9 mg, 0.44 mmol). The reaction mixture was purified by column chromatography (using pentane as eluent) affording product **14** (33.4 mg, 48% yield) as a colorless oil. The spectroscopic data are consistent with those reported previously.[28]

^{1}H NMR (300 MHz, $CDCl_3$) δ 7.14 (s, 4H), 2.54 – 2.45 (m, 1H), 2.35 (s, 3H), 1.94 – 1.74 (m, 5H), 1.49 – 1.26 (m, 5H).

^{13}C{1H} NMR (75 MHz, $CDCl_3$) δ 145.3, 135.3, 129.1, 126.8, 44.3, 34.7, 27.1, 26.3, 21.1.

1-Cyclohexyl-2-methylbenzene (15)

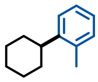

Prepared according to general procedure B using 1-iodo-2-methylbenzene (87.2 mg, 0.4 mmol) and 2,4,6-triphenylpyrylium tetrafluoroborate (209.9 mg, 0.44 mmol). The reaction mixture was purified by column chromatography (using pentane as eluent) affording product **15** (29.3 mg, 42% yield) as a colorless oil. The spectroscopic data are consistent with those reported previously.[29]

^{1}H NMR (300 MHz, $CDCl_3$) δ 7.26 – 7.09 (m, 4H), 2.76 – 2.69 (m, 1H), 2.35 (s, 3H), 1.93 – 1.74 (m, 5H), 1.49 – 1.25 (m, 5H).

^{13}C{1H} NMR (75 MHz, $CDCl_3$) δ 146.0, 135.2, 130.3, 126.2, 125.6, 125.5, 40.2, 33.8, 27.3, 26.5, 19.5.

HRMS (EI) m/z: $[M]^+$ calculated for $C_{13}H_{18}$: 174.1409, found: 174.1409.

1-Cyclohexyl-4-phyenylbenzene (16)

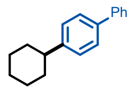

Prepared according to general procedure B, using 1-iodo-4-phenylbenzene (112.0 mg, 0.4 mmol) and 2,4,6-triphenylpyrylium tetrafluoroborate (209.9 mg, 0.44 mmol). The reaction mixture was purified by column chromatography (using pentane as eluent) affording product **16** (49.1 mg, 52% yield) as a colorless oil. The spectroscopic data are consistent with those reported previously.[30]

^{1}H NMR (300 MHz, $CDCl_3$) δ 7.67 – 7.55 (m, 4H), 7.47 (t, J = 7.4 Hz, 2H), 7.40 – 7.30 (m, 3H), 2.64 – 2.55 (m, 1H), 2.05 – 1.79 (m, 5H), 1.56 – 1.30 (m, 5H).

^{13}C{1H} NMR (75 MHz, $CDCl_3$) δ 147.4, 141.4, 138.9, 128.9, 127.4, 127.23, 127.21, 127.1, 44.4, 34.7, 27.1, 26.4.

HRMS (EI) m/z: $[M]^+$ calculated for $C_{18}H_{20}$: 236.1565, found: 236.1567.

4-Cyclohexylacetophenone (17)

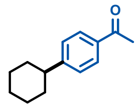

Prepared according to general procedure B, using 4-iodoacetophenone (98.4 mg, 0.4 mmol) and 2,4,6-triphenylpyrylium tetrafluoroborate (209.9 mg, 0.44 mmol). The reaction mixture was purified by column chromatography (gradient elution, pentane/ethyl acetate = 1/0 to pentane/ethyl acetate = 99/1) affording product **17** (45.3 mg, 56% yield) as a colorless oil. The spectroscopic data are consistent with those reported previously.[31]

^{1}H NMR (300 MHz, $CDCl_3$) δ 7.93 – 7.84 (m, 2H), 7.29 (d, J = 8.2 Hz, 2H), 2.58 (s, 3H), 2.57 – 2.54 (m, 1H), 1.89 – 1.75 (m, 5H), 1.56 – 1.25 (m, 5H).

^{13}C{1H} NMR (75 MHz, $CDCl_3$) δ 197.5, 153.4, 134.7, 128.2, 126.7, 123.4, 116.3, 44.4, 33.8, 26.4, 26.2, 25.7.

HRMS (FI) m/z: $[M]^+$ calculated for $C_{14}H_{18}O$: 202.1358, found: 202.1358.

4-Cyclohexylbenzaldehyde (18)

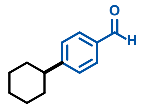

Prepared according to general procedure B, using 4-iodobenzaldehyde (92.8 mg, 0.4 mmol) and 2,4,6-triphenylpyrylium tetrafluoroborate (209.9 mg, 0.44 mmol). The reaction mixture was purified by column chromatography (gradient elution, pentane/chloroform = 1/0 to 95/5 pentane/chloroform) affording product **18** (20.3 mg, 27% yield) as a colorless oil. The spectroscopic data are consistent with those reported previously.[1]

^{1}H NMR (300 MHz, $CDCl_3$) δ 9.96 (s, 1H), 7.80 (d, J = 8.2 Hz, 2H), 7.36 (d, J = 8.2 Hz, 2H), 2.62 – 2.56 (m, 1H), 1.91 – 1.84 (m, 4H), 1.75 (d, J = 2.7 Hz, 1H), 1.50 – 1.39 (m, 4H), 1.30 – 1.24 (m, 1H).

^{13}C{1H} NMR (75 MHz, $CDCl_3$) δ 192.2, 155.5, 134.6, 130.1, 127.6, 45.0, 34.2, 26.8, 26.1.

2-Chloro-5-Cyclohexylpyridine (19)

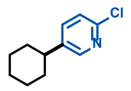

Prepared according to general procedure B, using 2-chloro-5-iodopyridine (95.8 mg, 0.4 mmol) and 2,4,6-triphenylpyrylium tetrafluoroborate (209.9 mg, 0.44 mmol). The reaction mixture was purified first by normal-phase silica column chromatography (gradient elution, pentane/ethyl acetate = 1/0 to pentane/ethyl acetate = 95/5) and second by reversed-phase silica column (gradient elution, acetonitrile/water = 5/95 to acetonitrile/water = 99/1 with 0.1% formic acid) affording product **19** (33.0 mg, 42% yield) as a colorless oil.

^{1}H NMR (300 MHz, $CDCl_3$) δ 8.27 (d, J = 2.5 Hz, 1H), 7.52 (dd, J = 8.2, 2.5 Hz, 1H), 7.29 (d, J = 8.2 Hz, 1H), 2.64 – 2.48 (m, 1H), 1.89 (m, 4H), 1.84 – 1.76 (m, 1H), 1.49 – 1.37 (m, 4H), 1.36 – 1.34 (m, 1H).

^{13}C{1H} NMR (75 MHz, $CDCl_3$) δ 155.4, 148.7, 142.0, 137.3, 124.0, 41.5, 34.2, 26.7, 26.0.

HRMS (FI) m/z: $[M]^+$ calculated for $C_{11}H_{14}ClN$: 195.0815, found: 195.0823.

Menthol-4-cyclohexylbenzoate (20)

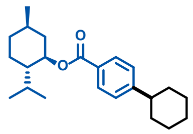

Prepared according to general procedure B, using menthol-4-iodobenzoate (154.4 mg, 0.4 mmol) and 2,4,6-triphenylpyrylium tetrafluoroborate (209.9 mg, 0.44 mmol). The reaction mixture was purified first by normal-phase silica column chromatography (gradient elution, pentane/diethyl ether = 1/0 to pentane/diethyl ether = 99/1) and second by reversed-phase silica column chromatography (acetonitrile/water = 60/40 with 0.1% formic acid) affording product **20** (94.7 mg, 69% yield) as a colorless oil.

^{1}H NMR (300 MHz, $CDCl_3$) δ 7.96 (d, J = 8.3 Hz, 2H), 7.27 (d, J = 8.3 Hz, 2H), 4.91 (td, J = 10.8, 4.4 Hz, 1H), 2.63 – 2.50 (m, 1H), 2.13 – 2.08 (m, 1H), 1.96 (dtd, J = 13.8, 6.9, 2.6 Hz, 1H), 1.90 – 1.85 (m, 4H), 1.78 – 1.69 (m, 3H), 1.60 – 1.55 (m, 1H), 1.54 – 1.50 (m, 1H), 1.47 – 1.36 (m, 4H), 1.32 – 1.28 (m, 2H), 1.20 – 1.08 (m, 2H), 0.93 (d, J = 7.0 Hz, 3H), 0.91 (d, J = 7.0, 3H), 0.78 (d, J = 6.9 Hz, 3H).

^{13}C{1H} NMR (75 MHz, $CDCl_3$) δ 166.3, 153.4, 129.8, 128.6, 127.0, 74.7, 47.4, 44.8, 41.2, 34.5, 34.33, 34.31, 31.6, 26.9, 26.6, 26.2, 23.8, 22.2, 20.9, 16.7.

HRMS (FI) m/z: $[M]^+$ calculated for $C_{23}H_{34}O_2$: 342.2553, found: 342.2559.

3-Methoxy-17-estradiol-4-cyclohexylbenzoate (21)

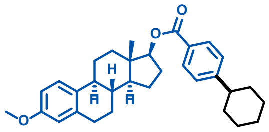

Prepared according to general procedure B, using 3-methoxy-17-estradiol-4-iodobenzoate (206.6 mg, 0.4 mmol) and 2,4,6-triphenylpyrylium tetrafluoroborate (209.9 mg, 0.44 mmol). The reaction mixture was purified by column chromatography (gradient elution, pentane/chloroform = 1/0 to pentane/chloroform = 1/1) affording product **21** (75.6 mg, 40% yield) as a colorless oil.

^{1}H NMR (300 MHz, $CDCl_3$) δ 7.97 (d, J = 8.3 Hz, 2H), 7.32 – 7.17 (m, 3H), 6.71 (dd, J = 8.5, 2.8 Hz, 1H), 6.64 (d, J = 2.8 Hz, 1H), 4.92 (dd, J = 9.1, 7.6 Hz, 1H), 3.78 (s, 3H), 2.88 (q, J = 5.1 Hz, 2H), 2.56 (s, 1H), 2.46 – 2.21 (m, 3H), 2.01 – 1.60 (m, 9H), 1.58 – 1.19 (m, 15H).

^{13}C{1H} NMR (75 MHz, $CDCl_3$) δ 166.8, 157.6, 153.5, 138.1, 132.7, 129.8, 128.5, 127.0, 126.5, 114.0, 111.6, 83.2, 55.4, 50.0, 44.9, 44.0, 43.5, 38.8, 37.2, 34.3, 30.0, 28.0, 27.4, 26.9, 26.4, 26.2, 23.6, 12.5.

HRMS (FD) m/z: $[M]^+$ calculated for $C_{32}H_{40}O_3$: 472.2973, found: 472.2977.

Amine scope

Methyl 4-(4-phenylbutan-2-yl)benzoate (22)

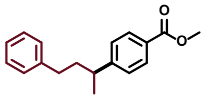

Prepared according to general procedure B, using methyl 4-iodobenzoate (104.8 mg, 0.4 mmol) and 2,4,6-triphenylpyrylium tetrafluoroborate (232.1 mg, 0.44 mmol). The reaction mixture was purified by column chromatography (gradient elution, pentane/ethyl acetate = 1/0 to pentane/ethyl acetate = 1/1) affording product **22** (60.9 mg, 57% yield) as a slightly yellow oil. The spectroscopic data are consistent with those reported previously.[26]

^{1}H NMR (300 MHz, $CDCl_3$) δ 7.99 (d, J = 8.3 Hz, 2H), 7.28 – 7.23 (m, 4H), 7.18 (d, J = 7.2 Hz, 1H), 7.13 – 7.08 (m, 2H), 3.91 (s, 3H), 2.78 (h, J = 7.0 Hz, 1H), 2.56 – 2.46 (m, 2H), 1.93 (q, J = 7.5 Hz, 2H), 1.27 (d, J = 7.0 Hz, 3H).

^{13}C{1H} NMR (75 MHz, $CDCl_3$) δ 167.3, 152.9, 142.3, 130.0, 128.5 (2C), 128.2, 127.3, 125.9, 52.1, 39.8, 39.7, 33.9, 22.3.

HRMS (FI) m/z: $[M]^+$ calculated for $C_{18}H_{20}O_2$: 268.1463, found: 268.1458.

Methyl 4-(heptan-2-yl)benzoate (23)

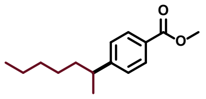

Prepared according to general procedure B, using methyl 4-iodobenzoate (104.8 mg, 0.4 mmol) and 2,4,6-triphenylpyrylium tetrafluoroborate (217.1 mg, 0.44 mmol). The reaction mixture was purified by column chromatography (gradient elution, pentane/ethyl acetate = 1/0 to pentane/ethyl acetate = 9/1) affording product **23** (43.1 mg, 46% yield) as a colorless solid. The spectroscopic data are consistent with those reported previously.[32]

^{1}H NMR (300 MHz, $CDCl_3$) δ 7.96 (d, J = 8.3 Hz, 2H), 7.25 (d, J = 8.3 Hz, 2H), 3.90 (s, 3H), 2.74 (h, J = 7.0 Hz, 1H), 1.61 – 1.53 (m, 2H), 1.24 –1.06 (br m, 9H), 0.89 – 0.81 (m, 3H).

^{13}C NMR (75 MHz, $CDCl_3$) δ 167.3, 153.7, 129.8, 127.9, 127.2, 52.1, 40.2, 38.3, 32.0, 27.4, 22.7, 22.2, 14.2.

HRMS (FD) m/z: $[M]^+$ calculated for $C_{15}H_{22}O_2$: 234.1620, found: 234.1614.

Methyl 4-isopropylbenzoate (24)

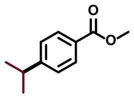

Prepared according to general procedure B, using methyl 4-iodobenzoate (104.8 mg, 0.4 mmol) and 2,4,6-triphenylpyrylium tetrafluoroborate (192.4 mg, 0.44 mmol). The reaction mixture was purified by column chromatography (gradient elution, pentane/chloroform = 1/0 to pentane/chloroform = 4/1) affording product **24** (39.9 mg, 56% yield) as a colorless oil. The spectroscopic data are consistent with those reported previously.[33]

^{1}H NMR (300 MHz, $CDCl_3$) δ 7.96 (d, J = 8.3 Hz, 2H), 7.29 (d, J = 8.3 Hz, 2H), 3.90 (s, 3H), 2.96 (hept, J = 6.9 Hz, 1H), 1.27 (d, J = 6.9 Hz, 6H).

^{13}C{1H} NMR (75 MHz, $CDCl_3$) δ 167.4, 154.5, 129.9, 128.0, 126.7, 52.1, 34.4, 23.9.

HRMS (FI) m/z: $[M]^+$ calculated for $C_{11}H_{14}O_2$: 178.0994, found: 178.0995.

Methyl 4-sec-buthylbenzoate (25)

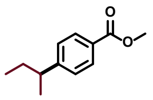

Prepared according to general procedure B, using methyl 4-iodobenzoate (104.8 mg, 0.4 mmol) and 2,4,6-triphenylpyrylium tetrafluoroborate (198.6 mg, 0.44 mmol). The reaction mixture was purified by column chromatography (gradient elution, pentane/chloroform = 1/0 to pentane/chloroform = 4/1) affording product **25** (44.6 mg, 58% yield) as a colorless solid. The spectroscopic data are consistent with those reported previously.[33]

^{1}H NMR (300 MHz, $CDCl_3$) δ 7.96 (d, J = 8.3 Hz, 2H), 7.24 (d, J = 8.3 Hz, 2H), 3.90 (s, 3H), 2.66 (h, J = 7.0 Hz, 1H), 1.62 (q, J = 7.4 Hz, 2H), 1.25 (d, J = 7.0 Hz, 3H), 0.81 (t, J = 7.4 Hz, 3H).

^{13}C{1H} NMR (75 MHz, $CDCl_3$) δ 167.4, 153.4, 129.8, 128.0, 127.3, 52.1, 42.0, 31.1, 21.8, 12.3.

HRMS (FI) m/z: $[M]^+$ calculated for $C_{12}H_{16}O_2$: 192.1150, found: 192.1145.

Methyl 4-(pentan-3-yl)benzoate (26)

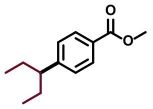

Prepared according to general procedure B, using methyl 4-iodobenzoate (104.8 mg, 0.4 mmol) and 2,4,6-triphenylpyrylium tetrafluoroborate (204.8 mg, 0.44 mmol). The reaction mixture was purified by column chromatography (gradient elution, pentane/chloroform = 1/0 to pentane/chloroform = 7/3) affording product **26** (46.2 mg, 56% yield) as a colorless oil.

^{1}H NMR (300 MHz, $CDCl_3$) δ 7.96 (d, J = 8.3 Hz, 2H), 7.20 (d, J = 8.3 Hz, 2H), 3.90 (s, 3H), 2.39 (tt, J = 9.2, 5.4 Hz, 1H), 1.70 (m, 2H), 1.60 – 1.48 (m, 2H), 0.76 (t, J = 7.4 Hz, 6H).

^{13}C NMR (75 MHz, $CDCl_3$) δ 167.4, 151.7, 129.7, 128.0, 52.1, 49.9, 29.3, 12.2.

HRMS (EI) m/z: $[M]^+$ calculated for $C_{13}H_{18}O_2$: 206.1307, found: 206.1310.

Methyl 4-cyclopentylbenzoate (27)

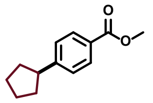

Prepared according to general procedure B, using methyl 4-iodobenzoate (104.8 mg, 0.4 mmol) and 2,4,6-triphenylpyrylium tetrafluoroborate (203.8 mg, 0.44 mmol). The reaction mixture was purified by column chromatography (pentane/toluene = 3/7) affording product **27** (34.3 mg, 42% yield) as a colorless oil. The spectroscopic data are consistent with those reported previously.[32]

^{1}H NMR (300 MHz, $CDCl_3$) δ 7.95 (d, J = 8.2 Hz, 2H), 7.32 – 7.28 (m, 2H), 3.90 (s, 3H), 3.13 – 2.97 (m, 1H), 2.14 – 2.03 (m, 2H), 1.85 – 1.55 (m, 6H).

^{13}C{1H} NMR (75 MHz, $CDCl_3$) δ 167.3, 152.4, 129.7, 127.8, 127.3, 52.1, 46.1, 35.6, 26.3.

HRMS (FI) m/z: $[M]^+$ calculated for $C_{13}H_{16}O_2$: 204.1150, found: 201.1142.

Methyl 4-cycloheptylbenzoate (28)

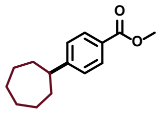

Prepared according to general procedure B, using methyl 4-iodobenzoate (104.8 mg, 0.4 mmol) and 2,4,6-triphenylpyrylium tetrafluoroborate (216.2 mg, 0.44 mmol). The reaction mixture was purified first by normal-phase silica column chromatography (pentane/toluene = 4/6) and second by reversed-phased silica column chromatography (gradient elution, acetonitrile/water = 5/95 to acetonitrile/water = 99/1 with 0.1% formic acid) affording product **28** (52.9 mg, 57% yield) as colorless solid. The spectroscopic data are consistent with those reported previously.[32]

^{1}H NMR (300 MHz, $CDCl_3$) δ 7.94 (d, J = 8.3 Hz, 2H), 7.25 (d, J = 8.3 Hz, 2H), 3.89 (s, 3H), 2.77 – 2.66 (m, 1H), 1.90 – 1.55 (m, 12H).

^{13}C{1H} NMR (75 MHz, $CDCl_3$) δ 167.3, 155.5, 129.9, 127.6, 126.9, 52.1, 47.2, 36.6, 28.0, 27.4.

4-(tetrahydro-2H-pyran-4-yl)benzonitrile (29)

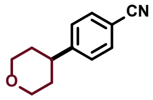

Prepared according to general procedure B1, using 4-iodobenzonitrile (91.6 mg, 0.4 mmol) and 2,4,6-triphenylpyrylium tetrafluoroborate (210.9 mg, 0.44 mmol). The reaction mixture was purified first by column chromatography (gradient elution, pentane/ethyl acetate = 1/0 to pentane/ethyl acetate = 95/5) and second by another column (pentane/chloroform = 1/0 to pentane/chloroform = 3/7) affording product **29** (49.4 mg, 66% yield) as a colorless oil. The spectroscopic data are consistent with those reported previously.[32]

^{1}H NMR (300 MHz, $CDCl_3$) δ 7.64 – 7.58 (m, 2H), 7.36 – 7.29 (m, 2H), 4.16 – 4.03 (m, 2H), 3.53 (td, J = 11.3, 3.3 Hz, 2H), 2.90 – 2.75 (m, 1H), 1.86 – 1.74 (m, 4H).

^{13}C NMR (75 MHz, $CDCl_3$) δ 151.3, 132.6, 127.8, 119.1, 110.4, 68.2, 41.9, 33.5.

HRMS (FI) m/z: $[M]^+$ calculated for $C_{12}H_{13}NO$: 187.0997, found: 187.0997.

4-(4-chlorophenyl)tetrahydro-2H-pyran (30)

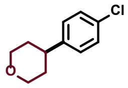

Prepared according to general procedure B, using 1-chloro-4-iodobenzene (95.4 mg, 0.4 mmol) and 2,4,6-triphenylpyrylium tetrafluoroborate (210.9 mg, 0.44 mmol). The reaction mixture was purified by column chromatography (gradient elution, pentane/chloroform = 1/0 to pentane/chloroform 3/7) affording product **30** (15.7 mg, 20% yield) as a colorless oil. The spectroscopic data are consistent with those reported previously.[34]

^{1}H NMR (300 MHz, $CDCl_3$) δ 7.33 – 7.23 (m, 2H), 7.21 – 7.10 (m, 2H), 4.13 – 4.03 (m, 2H), 3.52 (dd, J = 14.5, 11.3 Hz, 2H), 2.81 – 2.66 (m, 1H), 1.85 – 1.68 (m, 4H).

^{13}C{1H} NMR (75 MHz, $CDCl_3$) δ 144.4, 132.1, 128.8, 128.2, 68.4, 41.1, 34.0.

HRMS (FI) m/z: $[M]^+$ calculated for $C_{11}H_{13}ClO$: 196.0655, found: 196.0660.

Methyl 4-(tetrahydro-2H-pyran-4-yl)benzoate (31)

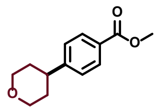

Prepared according to general procedure B, using methyl 4-iodobenzoate (104.8 mg, 0.4 mmol) and 2,4,6-triphenylpyrylium tetrafluoroborate (210.9 mg, 0.44 mmol). The reaction mixture was purified first by column chromatography (gradient elution, pentane/ethyl acetate = 1/0 to pentane/ethyl acetate = 1/1) and second by preparative TLC (pentane/toluene = 4/6) affording product **31** (59.9 mg, 68% yield) as a colorless solid. The spectroscopic data are consistent with those reported previously.[33]

^{1}H NMR (300 MHz, $CDCl_3$) δ 7.97 (d, J = 8.3 Hz, 2H), 7.27 (d, J = 8.4 Hz, 2H), 4.11 – 4.03 (m, 2H), 3.88 (s, 3H), 3.51 (td, J = 11.4, 3.0 Hz, 2H), 2.79 (td, J = 11.0, 4.8 Hz, 1H), 1.89 – 1.70 (m, 4H).

^{13}C{1H} NMR (75 MHz, $CDCl_3$) δ 167.1, 151.2, 130.0, 128.4, 126.9, 68.3, 52.1, 41.7, 33.7.

HRMS (FI) m/z: $[M]^+$ calculated for $C_{13}H_{16}O_3$: 220.1099, found: 220.1090.

Methyl 4-(1-hydroxy-3-phenylpropan-2-yl)benzoate (32)

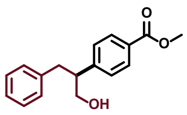

Prepared according to general procedure B, using methyl 4-iodobenzoate (104.8 mg, 0.4 mmol) and 2,4,6-triphenylpyrylium tetrafluoroborate (232.9 mg, 0.44 mmol). The reaction mixture was purified by column chromatography (gradient elution, pentane/ethyl acetate = 1/0 to pentane/ethyl acetate = 4/1) affording product **32** (56.2 mg, 52% yield) as a colorless oil.

^{1}H NMR (300 MHz, $CDCl_3$) δ 7.97 (d, J = 8.3 Hz, 2H), 7.27 (d, J = 8.3 Hz, 2H), 7.21 – 7.14 (m, 3H), 7.09 – 7.03 (m, 2H), 3.90 (s, 3H), 3.86 – 3.81 (m, 2H), 3.22 – 3.02 (m, 2H), 2.90 (dd, J = 13.4, 7.8 Hz, 1H).

^{13}C NMR (75 MHz, $CDCl_3$) δ 167.1, 147.7, 139.5, 130.0, 129.1, 128.9, 128.5, 128.3, 126.3, 66.2, 52.2, 50.4, 38.7.

HRMS (FI) m/z: $[M]^+$ calculated for $C_{17}H_{18}O_3$: 270.1256, found: 270.1254.

Methyl 4-(1-hydroxy-3-methylbutan-2-yl)benzoate (33)

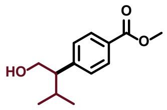

Prepared according to general procedure B, using methyl 4-iodobenzoate (104.8 mg, 0.4 mmol) and 2,4,6-triphenylpyrylium tetrafluoroborate (211.8 mg, 0.44 mmol). The reaction mixture was purified by column chromatography (gradient elution, pentane/ethyl acetate = 1/0 to pentane/ethyl acetate = 7/3) affording product **33** (32.9 mg, 37% yield) as a colorless oil.

^{1}H NMR (300 MHz, $CDCl_3$) δ 7.99 (d, J = 8.3 Hz, 2H), 7.27 (d, J = 8.3 Hz, 2H), 4.03 – 3.80 (m, 5H), 2.58 (td, J = 8.6, 4.9 Hz, 1H), 1.97 (dhept, J = 8.6, 6.7 Hz, 1H), 1.01 (d, J = 6.7 Hz, 3H), 0.72 (d, J = 6.7 Hz, 3H).

^{13}C{1H} NMR (75 MHz, $CDCl_3$) δ 167.2, 147.8, 129.9, 128.9, 128.7, 65.0, 55.9, 52.2, 30.1, 21.1, 21.0.

HRMS (EI) m/z: $[M]^+$ calculated for $C_{13}H_{18}O_3$: 222.1256, found: 222.1261.

Methyl 4-(4-hydroxycyclohexyl)benzoate (34a, iso-1**)**

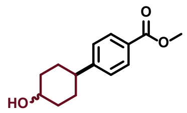

Prepared according to general procedure B, using methyl 4-iodobenzoate (104.8 mg, 0.4 mmol) and 2,4,6-triphenylpyrylium tetrafluoroborate (217.1 mg, 0.44 mmol). The reaction mixture was purified by column chromatography (gradient elution, pentane/ethyl acetate = 1/0 to pentane/ethyl acetate = 8/2) affording product **1ma** (36.0 mg, 39% yield) as a colorless oil.

^{1}H NMR (300 MHz, $CDCl_3$) δ 7.96 (d, J = 8.4 Hz, 2H), 7.30 (d, J = 8.4 Hz, 2H), 4.16 – 4.12 (m, 1H), 3.89 (s, 3H), 2.67 – 2.53 (m, 1H), 1.97 – 1.83 (m, 4H), 1.68 (td, J = 8.9, 3.3 Hz, 4H).

^{13}C{1H} NMR (75 MHz, $CDCl_3$) δ 167.3, 152.9, 129.9, 128.0, 127.0, 65.6, 52.1, 44.1, 33.0, 27.6.

HRMS (FI) m/z: $[M]^+$ calculated for $C_{14}H_{18}O_3$: 234.1256, found: 234.1248.

Methyl 4-(4-hydroxycyclohexyl)benzoate (34b, iso-2**)**

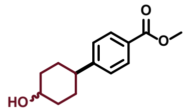

Prepared according to general procedure B, using methyl 4-iodobenzoate (104.8 mg, 0.4 mmol) and 2,4,6-triphenylpyrylium tetrafluoroborate (217.1 mg, 0.44 mmol). The reaction mixture was purified by column chromatography (gradient elution, pentane/ethyl acetate = 1/0 to pentane/ethyl acetate = 8/2) affording product **1mb** (38.0 mg, 41% yield) as a colorless oil.

^{1}H NMR (300 MHz, $CDCl_3$) δ 7.95 (d, J = 8.3 Hz, 2H), 7.26 (d, J = 8.3 Hz, 2H), 3.89 (s, 3H), 3.74 – 3.62 (m, 1H), 2.55 (tt, J = 11.9, 3.5 Hz, 1H), 2.16 – 2.07 (m, 2H), 1.99 – 1.86 (m, 2H), 1.60 – 1.38 (m, 4H).

^{13}C NMR (75 MHz, $CDCl_3$) δ 167.3, 152.1, 129.9, 128.2, 127.0, 70.6, 52.1, 43.7, 35.9, 32.3.

HRMS (FI) m/z: $[M]^+$ calculated for $C_{14}H_{18}O_3$: 234.1256, found: 234.1248.

tert-butyl 4-(4-methoxycarbonylphenyl)piperidine-1-carboxylate (35)

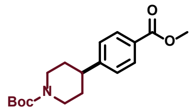

Prepared according to general procedure B, using methyl 4-iodobenzoate (172.9 mg, 0.66 mmol) and 2,4,6-triphenylpyrylium tetrafluoroborate (254.5 mg, 0.44 mmol). The reaction mixture was purified by column chromatography (gradient elution, pentane/diethyl ether = 1/0 to pentane/diethyl ether = 85/15) affording product **35** (80.4 mg, 63% yield) as a colorless solid. The spectroscopic data are consistent with those reported previously.[26]

^{1}H NMR (300 MHz, $CDCl_3$) δ 7.98 (d, J = 8.2 Hz, 2H), 7.27 (d, J = 8.2 Hz, 2H), 4.26 (br, 2H), 3.90 (s, 3H), 2.85 – 2.76 (br t, J = 12.4 Hz, 2H), 2.73 – 2.65 (overlapping tt, J = 12.0 Hz, 1H), 1.85 –1.70 (m, 2H), 1.63 (qd, J = 12.4, 4.2 Hz, 2H), 1.49 (s, 9H).

^{13}C{1H} NMR (75 MHz, $CDCl_3$) δ 167.1, 154.9, 151.1, 130.0, 128.4, 126.9, 79.6, 52.1, 44.3, 42.9, 33.0, 28.6.

HRMS (FD) m/z: $[M]^+$ calculated for $C_{18}H_{25}NO_4$: 319.1784, found: 319.1777.

Methyl 4-1-(2,6-dimethylphenoxy)propan-2-yl)benzoate (36)

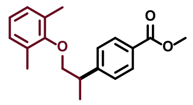

Prepared according to general procedure B, using methyl 4-iodobenzoate (104.8 mg, 0.4 mmol) and 2,4,6-triphenylpyrylium tetrafluoroborate (245.3 mg, 0.44 mmol). The reaction mixture was purified by column chromatography (gradient elution, pentane/chloroform = 1/0 to pentane/chloroform = 8/2) affording product **36** (45.3 mg, 38% yield) as a colorless solid. The spectroscopic data are consistent with those reported previously.[1]

^{1}H NMR (300 MHz, $CDCl_3$) δ 8.02 (d, J = 8.3 Hz, 2H), 7.41 (d, J = 8.4 Hz, 2H), 6.97 (d, J = 7.3 Hz, 2H), 6.89 (dd, J = 8.6, 6.1 Hz, 1H), 3.92 (s, 3H), 3.85 (dd, J = 6.5, 1.6 Hz, 2H), 3.33 (h, J = 6.8 Hz, 1H), 2.15 (s, 6H), 1.49 (d, J = 7.0 Hz, 3H).

^{13}C{1H} NMR (75 MHz, $CDCl_3$) δ 167.2, 155.6, 149.7, 131.0, 129.8, 128.9, 128.6, 127.7, 123.9, 76.8, 52.1, 40.9, 18.0, 16.2.

HRMS (FI) m/z: $[M]^+$ calculated for $C_{19}H_{22}O_3$: 298.1569, found: 298.1568.

Side product (Id)

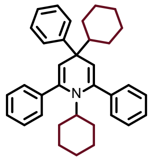

side product was formed via a radical-radical coupling between cyclohexyl radical with dihydropyridine radical. Product is reported by Wang et al.[15] The side product was purified by column chromatography (isocratic elution, pentane/toluene = 4/6) affording the product **Id** (19 mg, 20% yield - with respect to the Katritzky salt) as slightly yellow solid.

^{1}H NMR (300 MHz, $CDCl_3$) δ 7.63–7.60 (m, 4H), 7.45–7.30 (m, 10H), 7.16–7.12 (m, 1H), 5.28 (s, 1H), 2.84–2.76 (m, 1H), 1.66–1.38 (m, 6H), 1.43–1.27 (br, 4H), 1.20–0.94 (m, 6H), 0.91–0.84 (m, 1H), 0.78–0.59 (m, 4H)

MS (FD) m/z: $[M]^+$ calculated for $C_{35}H_{39}N$: 473.3, found: 473.3.

Upscaling of Coupling Reaction

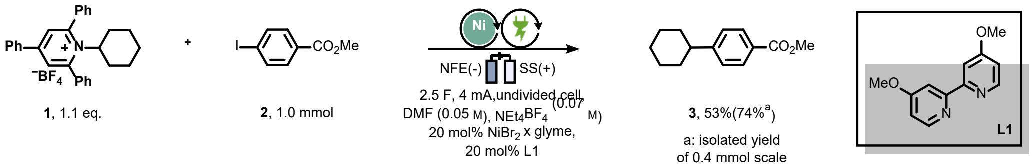

Prepared according to general procedure B, using methyl 4-iodobenzoate (262.0 mg, 1.0 mmol) and 2,4,6-triphenylpyrylium tetrafluoroborate (525.0 mg, 1.1 mmol). Concentration was adjusted to 0.1 M (10 mL reaction volume). Same reaction vessel was used as for the entire scope and no further optimization for the scale up was needed (electrode distance: 0.7 cm) The reaction mixture was purified by column chromatography (pentane/toluene = 4/6) affording product **3** (140 mg, 53% yield) as a colorless oil. The spectroscopic data are consistent with those reported previously.[21]

2. References

(1) Martin-Montero, R.; Yatham, V. R.; Yin, H.; Davies, J.; Martin, R. Ni-Catalyzed Reductive Deaminative Arylation at Sp 3 Carbon Centers. *Org. Lett.* **2019**, *21* (8), 2947–2951.

(2) Plunkett, S.; Basch, C. H.; Santana, S. O.; Watson, M. P. Harnessing Alkylpyridinium Salts as Electrophiles in Deaminative Alkyl–Alkyl Cross-Couplings. *J. Am. Chem. Soc.* **2019**, *141* (6), 2257–2262.

(3) Balssa, F.; Fischer, M.; Bonnaire, Y. An Easy Stereoselective Synthesis of 5(10)-Estrene-3β,17α-Diol, a Biological Marker of Pregnancy in the Mare. *Steroids* **2014**, *86*, 1–4.

(4) Huang, H.-M.; Bellotti, P.; Chen, P.-P.; Houk, K. N.; Glorius, F. Allylic C(Sp3)–H Arylation of Olefins via Ternary Catalysis. *Nat. Synth.* **2022**, *1* (1), 59–68.

(5) Zhang, B.; Gao, Y.; Hioki, Y.; Oderinde, M. S.; Qiao, J. X.; Rodriguez, K. X.; Zhang, H.-J.; Kawamata, Y.; Baran, P. S. Ni-Electrocatalytic Csp3–Csp3 Doubly Decarboxylative Coupling. *Nature* **2022**, *606* (7913), 313–318.

(6) Tsuji, K.; Elving, P. J. Conversion of Polarographic and Other Potentials from One Reference Electrode to Another. *Anal. Chem.* **1969**, *41* (1), 216–218.

(7) Haynes, W. M.; Lide, D. R.; Bruno, T. J. *CRC Handbook of Chemistry and Physics 97 Th Edition*.

(8) Truesdell, B. L.; Hamby, T. B.; Sevov, C. S. General C(Sp 2)–C(Sp 3) Cross-Electrophile Coupling Reactions Enabled by Overcharge Protection of Homogeneous Electrocatalysts. *J. Am. Chem. Soc.* **2020**, *142* (12), 5884–5893.

(9) Li, Q. L.; Guo, L. Y.; Shi, J.; Xiang, T. X.; Li, Q.; He, K. H.; Wang, B. Q.; Feng, C.; Pan, F. Nickel-Catalyzed Deaminative Cross-Coupling of Disulfides with Katritzky Pryidium Salts to Construct Sulfides. *Asian J. Org. Chem.* **2021**, *10* (10), 2525–2529.

(10) Kim, I.; Im, H.; Lee, H.; Hong, S. N-Heterocyclic Carbene-Catalyzed Deaminative Cross-Coupling of Aldehydes with Katritzky Pyridinium Salts. *Chem. Sci.* **2020**, *11* (12), 3192–3197.

(11) Franke, M. C.; Longley, V. R.; Rafiee, M.; Stahl, S. S.; Hansen, E. C.; Weix, D. J. Zinc-Free, Scalable Reductive Cross-Electrophile Coupling Driven by Electrochemistry in an Undivided Cell. *ACS Catal.* **2022**, *12* (20), 12617–12626.

(12) Wu, J.; Grant, P. S.; Li, X.; Noble, A.; Aggarwal, V. K. Catalyst-Free Deaminative Functionalizations of Primary Amines by Photoinduced Single-Electron Transfer. *Angew. Chemie Int. Ed.* **2019**, *58* (17), 5697–5701.

(13) Ociepa, M.; Turkowska, J.; Gryko, D. Redox-Activated Amines in C(Sp 3)–C(Sp) and C(Sp 3)–C(Sp 2) Bond Formation Enabled by Metal-Free Photoredox Catalysis. *ACS Catal.* **2018**, *8* (12), 11362–11367.

(14) Wang, K.; Liu, X.; Yang, S.; Tian, Y.; Zhou, M.; Zhou, J.; Jia, X.; Li, B.; Liu, S.; Chen, J. In Situ Alkyl Radical Recycling-Driven Decoupled Electrophotochemical Deamination. *Org. Lett.* **2022**, *24* (19), 3471–3476.

(15) Wang, C.; Qi, R.; Xue, H.; Shen, Y.; Chang, M.; Chen, Y.; Wang, R.; Xu, Z. Visible-Light-Promoted C(Sp 3)−H Alkylation by Intermolecular Charge Transfer: Preparation of Unnatural A-Amino Acids and Late-Stage Modification of Peptides. *Angew. Chemie Int. Ed.* **2020**, *59* (19), 7461–7466.

(16) Klein, A.; Feth, M. P.; Bertagnolli, H.; Záliš, S. On the Electronic Structure of Mesitylnickel Complexes of α-Diimines - Combining Structural Data, Spectroscopy and Calculations. *Eur. J. Inorg. Chem.* **2004**, No. 13, 2784–2796.

(17) Klein, A.; Kaiser, A.; Sarkar, B.; Wanner, M.; Fiedler, J. The Electrochemical Behaviour of Organonickel Complexes: Mono-, Di- and Trivalent Nickel. *Eur. J. Inorg. Chem.* **2007**,

No. 7, 965–976.

(18) Till, N. A.; Oh, S.; MacMillan, D. W. C.; Bird, M. J. The Application of Pulse Radiolysis to the Study of Ni(I) Intermediates in Ni-Catalyzed Cross-Coupling Reactions. *J. Am. Chem. Soc.* **2021**, *143* (25), 9332–9337.

(19) Steiman, T. J.; Liu, J.; Mengiste, A.; Doyle, A. G. Synthesis of β-Phenethylamines via Ni/Photoredox Cross-Electrophile Coupling of Aliphatic Aziridines and Aryl Iodides. *J. Am. Chem. Soc.* **2020**, *142* (16), 7598–7605.

(20) Ting, S. I.; Williams, W. L.; Doyle, A. G. Oxidative Addition of Aryl Halides to a Ni(I)-Bipyridine Complex. *J. Am. Chem. Soc.* **2022**, *144* (12), 5575–5582.

(21) Perry, I. B.; Brewer, T. F.; Sarver, P. J.; Schultz, D. M.; DiRocco, D. A.; MacMillan, D. W. C. Direct Arylation of Strong Aliphatic C–H Bonds. *Nature* **2018**, *560* (7716), 70–75.

(22) McLean, E. B.; Mooney, D. T.; Burns, D. J.; Lee, A.-L. Direct Hydrodecarboxylation of Aliphatic Carboxylic Acids: Metal- and Light-Free. *Org. Lett.* **2022**, *24* (2), 686–691.

(23) Zhou, J. (Steve); Fu, G. C. Suzuki Cross-Couplings of Unactivated Secondary Alkyl Bromides and Iodides. *J. Am. Chem. Soc.* **2004**, *126* (5), 1340–1341.

(24) Toriyama, F.; Cornella, J.; Wimmer, L.; Chen, T.-G.; Dixon, D. D.; Creech, G.; Baran, P. S. Redox-Active Esters in Fe-Catalyzed C–C Coupling. *J. Am. Chem. Soc.* **2016**, *138* (35), 11132–11135.

(25) Sun, Z.; Ji, R.; Wu, J.; Zhao, J.; Fang, F.; Wang, F.; Jiang, C.; Liu, Z.-Q. Electrochemical Deoxygenative Hydrogenation and Deuteration of Aldehydes/Ketones by Protic Acids in Water. *Adv. Synth. Catal.* **2023**, *365* (4), 476–481.

(26) Yedase, G. S.; Jha, A. K.; Yatham, V. R. Visible-Light Enabled C(s P3)-C(s P2) Cross-Electrophile Coupling via Synergistic Halogen-Atom Transfer (XAT) and Nickel Catalysis. *J. Org. Chem.* **2022**, *87* (8), 5442–5450.

(27) Lyons, D. J. M.; Dinh, A. H.; Ton, N. N. H.; Crocker, R. D.; Mai, B. K.; Nguyen, T. V. Ring Contraction of Tropylium Ions into Benzenoid Derivatives. *Org. Lett.* **2022**, *24* (13), 2520–2525.

(28) Han, B.; Ren, C.; Jiang, M.; Wu, L. Titanium-Catalyzed Exhaustive Reduction of Oxo-Chemicals. *Angew. Chemie - Int. Ed.* **2022**, *61* (46).

(29) Hashimoto, T.; Maruyama, T.; Yamaguchi, T.; Matsubara, Y.; Yamaguchi, Y. Cross-Coupling Reactions of Alkyl Halides with Aryl Grignard Reagents Using a Tetrachloroferrate with an Innocent Countercation. *Adv. Synth. Catal.* **2019**, *361* (18), 4232–4236.

(30) Zhang, G.; Guan, C.; Zhao, Y.; Miao, H.; Ding, C. 'Awaken' Aryl Sulfonyl Fluoride: A New Partner in the Suzuki–Miyaura Coupling Reaction. *New J. Chem.* **2022**, *46* (8), 3560–3564.

(31) Watanabe, E.; Chen, Y.; May, O.; Ley, S. V. A Practical Method for Continuous Production of Sp3-Rich Compounds from (Hetero)Aryl Halides and Redox-Active Esters. *Chem. - A Eur. J.* **2020**, *26* (1), 186–191.

(32) Zhang, P.; Le, C. C.; MacMillan, D. W. C. Silyl Radical Activation of Alkyl Halides in Metallaphotoredox Catalysis: A Unique Pathway for Cross-Electrophile Coupling. *J. Am. Chem. Soc.* **2016**, *138* (26), 8084–8087.

(33) Primer, D. N.; Karakaya, I.; Tellis, J. C.; Molander, G. A. Single-Electron Transmetalation: An Enabling Technology for Secondary Alkylboron Cross-Coupling. *J. Am. Chem. Soc.* **2015**, *137* (6), 2195–2198.

(34) Wang, H.; Zhao, Y.; Zhang, F.; Wu, Y.; Li, R.; Xiang, J.; Wang, Z.; Han, B.; Liu, Z. Hydrogen-Bonding Catalyzed Ring-Closing C−O/C−O Metathesis of Aliphatic Ethers over

Ionic Liquid under Metal-Free Conditions. *Angew. Chemie - Int. Ed.* **2020**, *59* (29), 11850–11855.

3. NMR Spectra

3, ^{1}H NMR (400 MHz, $CDCl_3$)

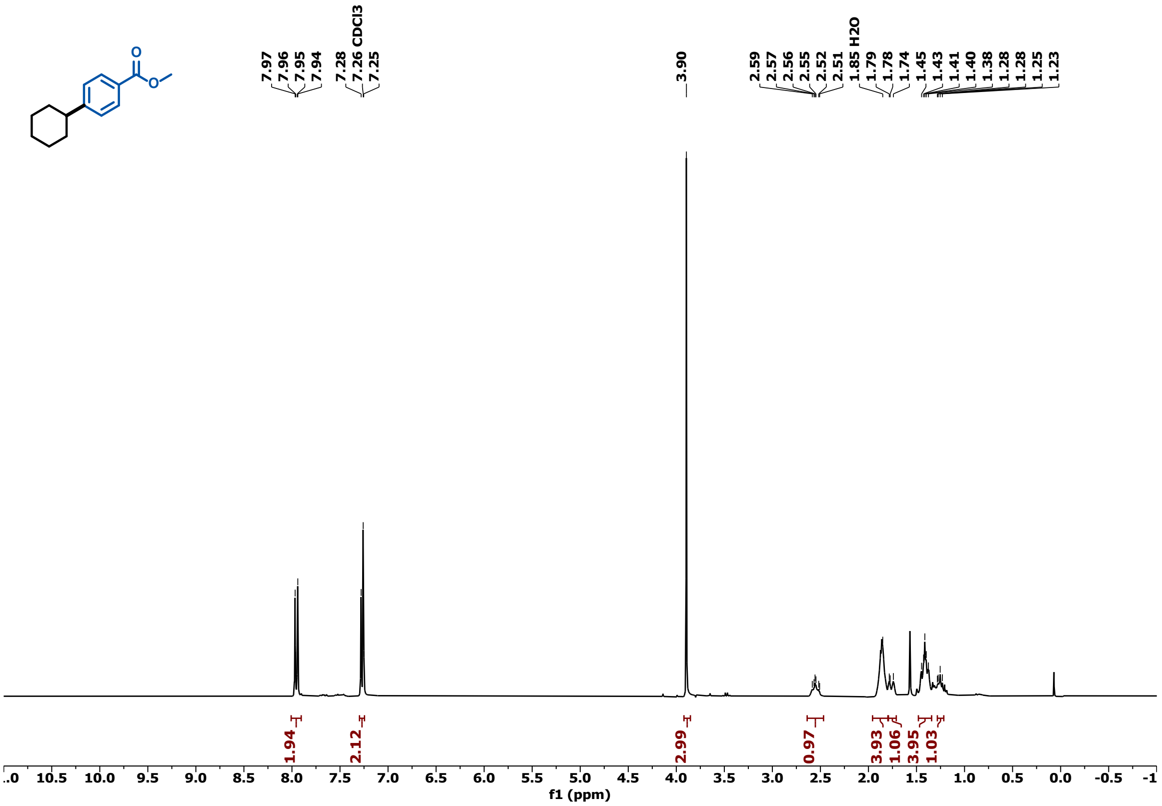

3, ^{13}C NMR (75 MHz, $CDCl_3$)

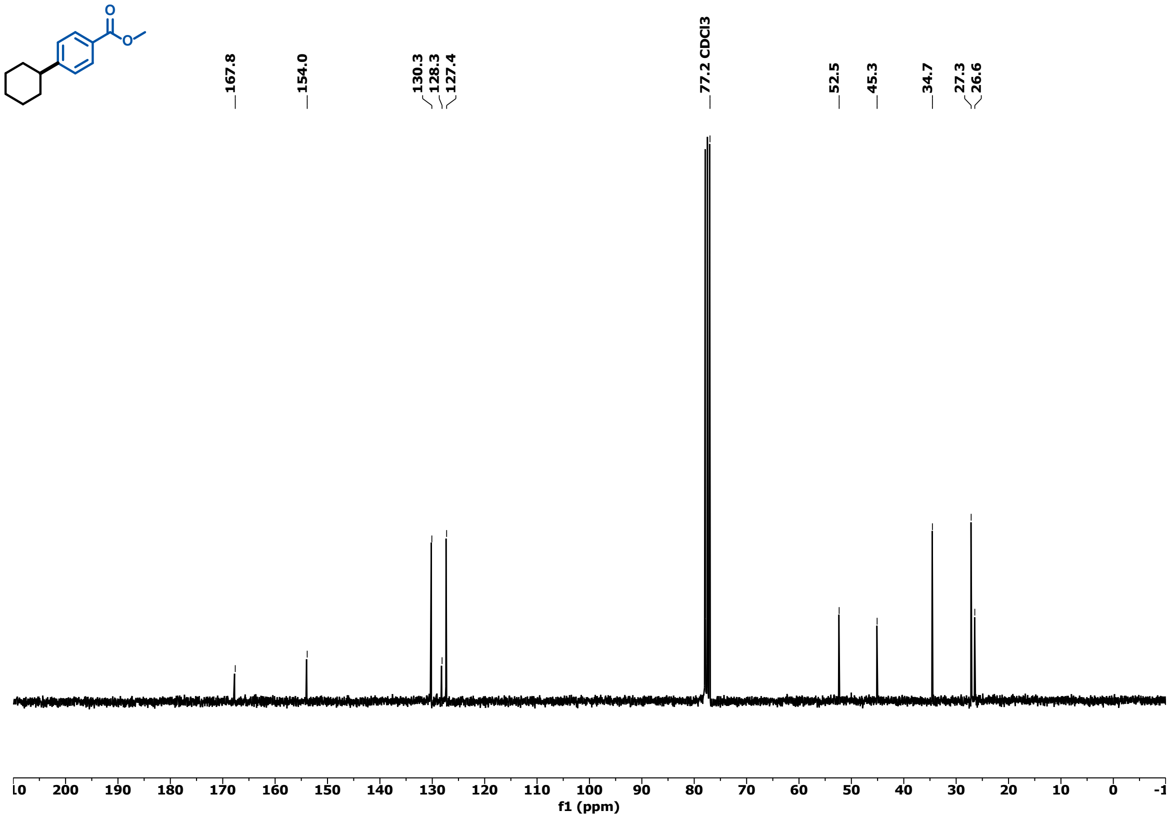

4, ^{1}H NMR (400 MHz, $CDCl_3$)

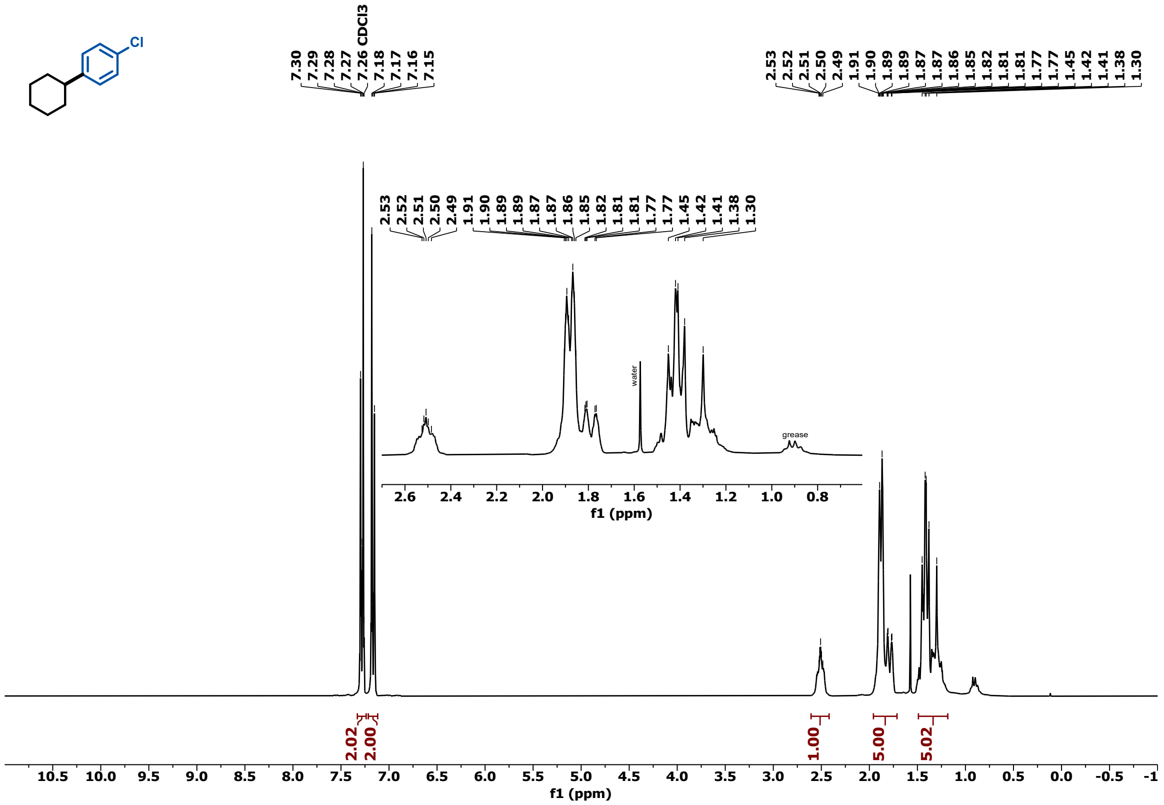

4, ^{13}C NMR (75 MHz, $CDCl_3$)

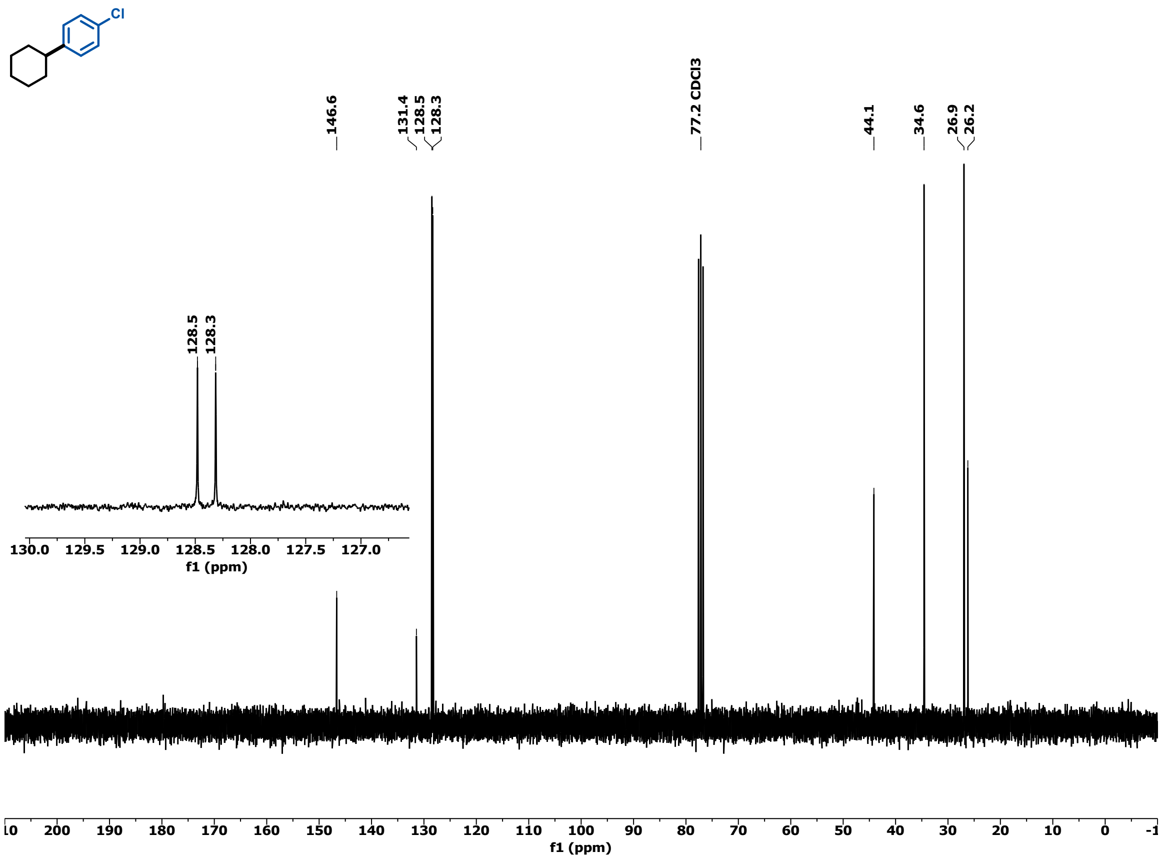

5, ^{1}H NMR (400 MHz, $CDCl_3$)

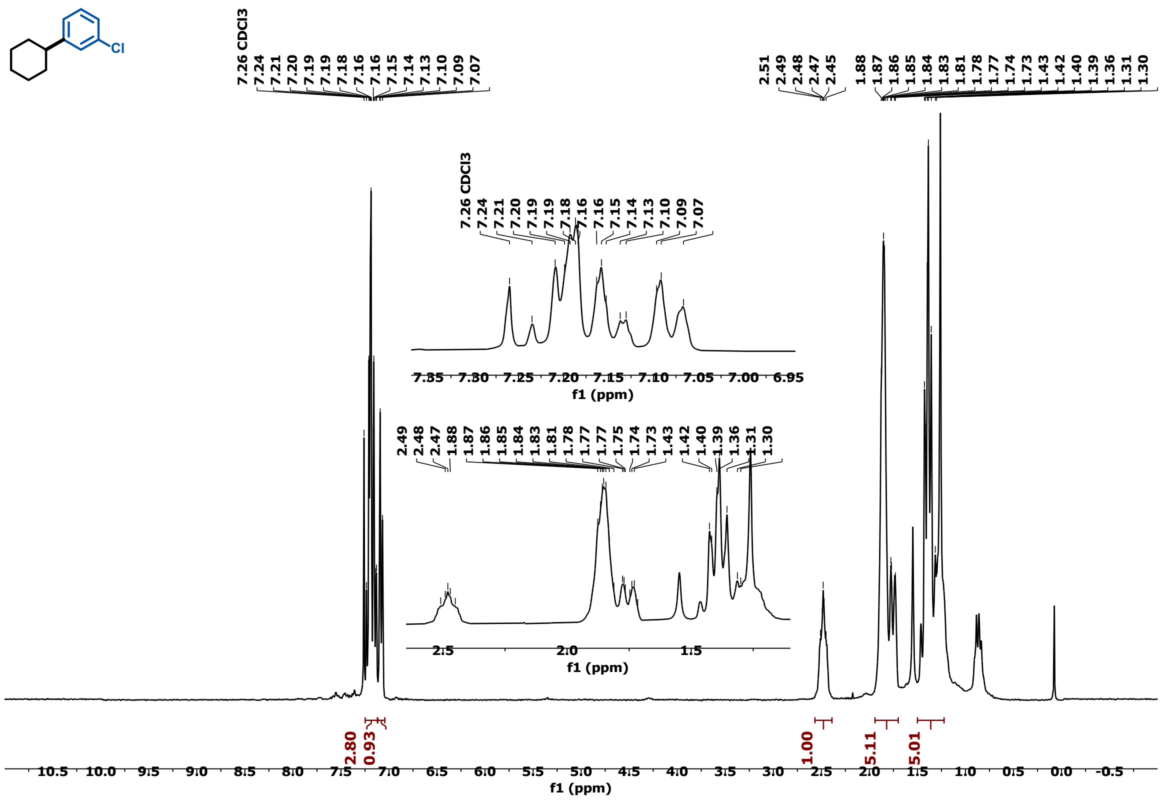

5, ^{13}C NMR (75 MHz, $CDCl_3$)

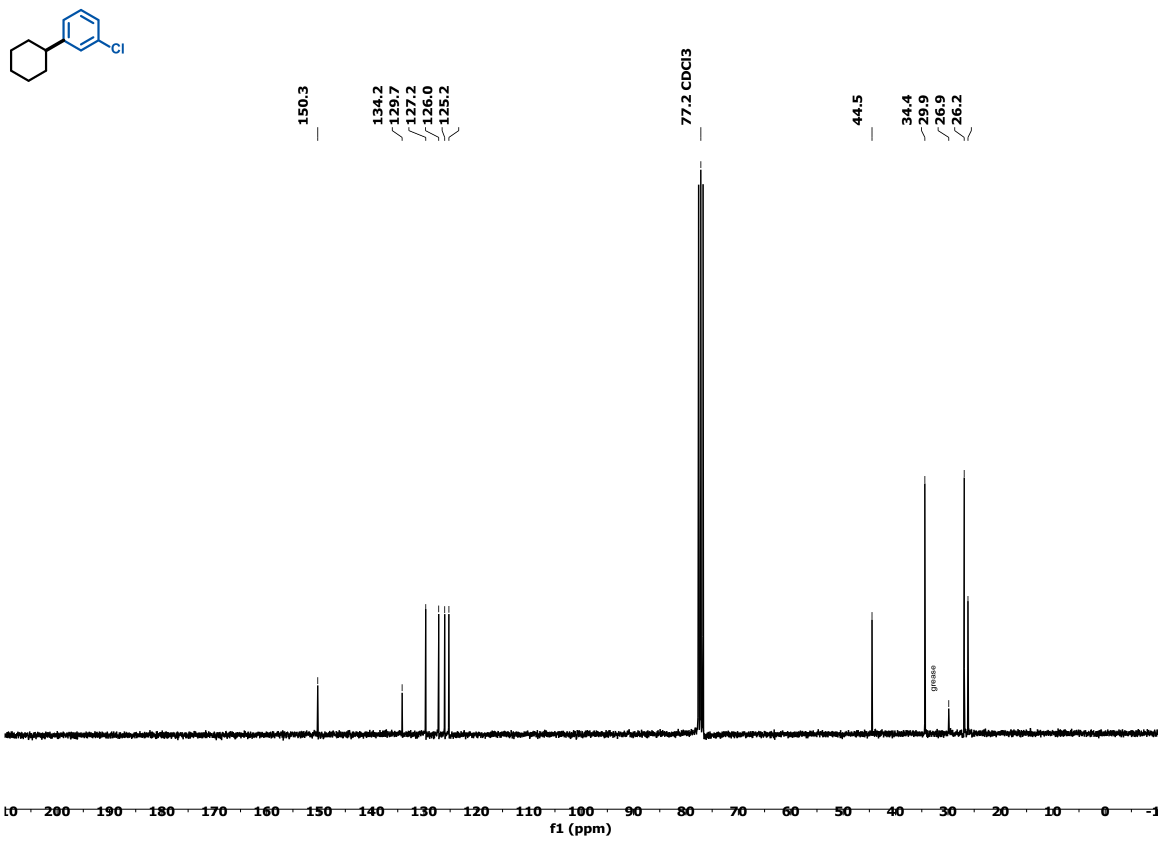

6, ^{1}H NMR (400 MHz, $CDCl_3$)

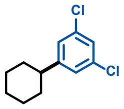

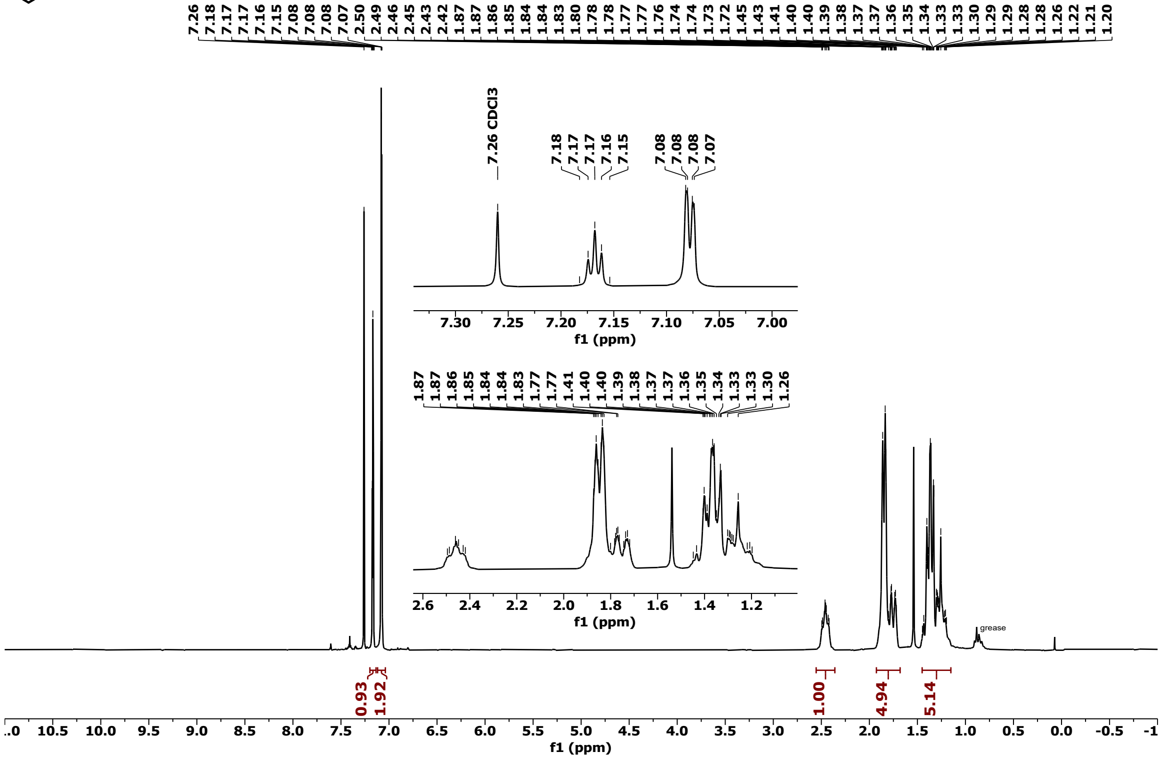

6, ^{13}C NMR (75 MHz, $CDCl_3$)

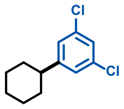

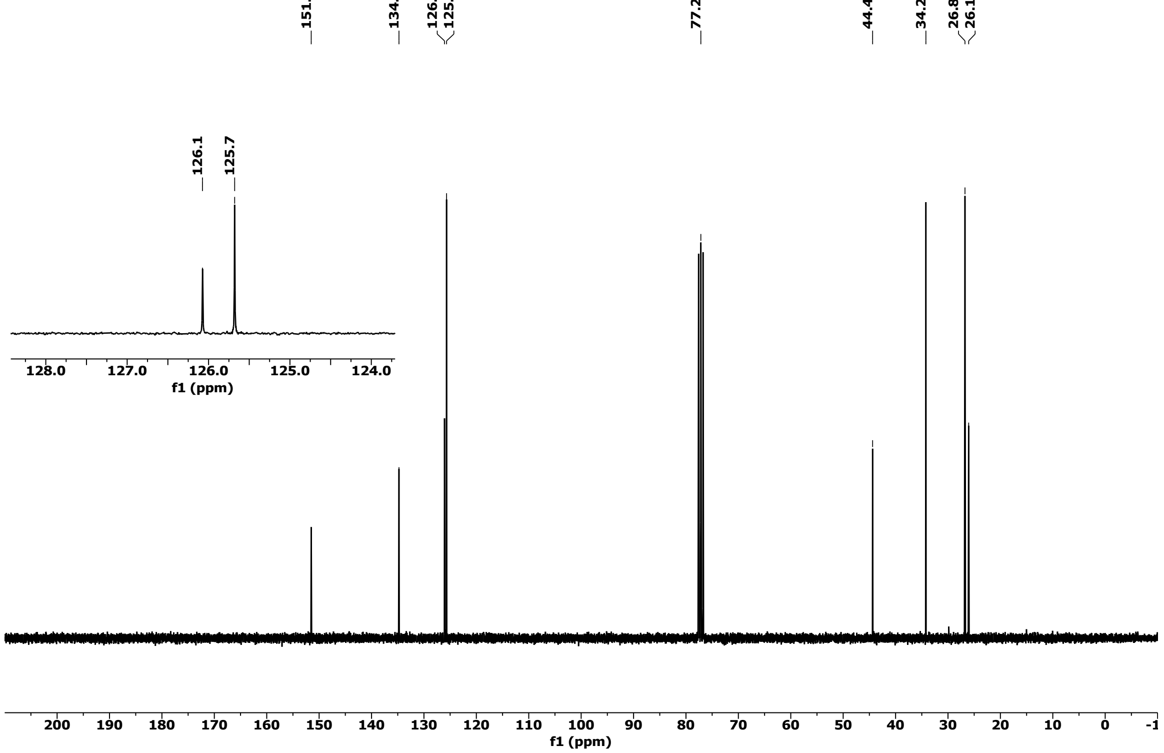

7, ^{1}H NMR (400 MHz, $CDCl_3$)

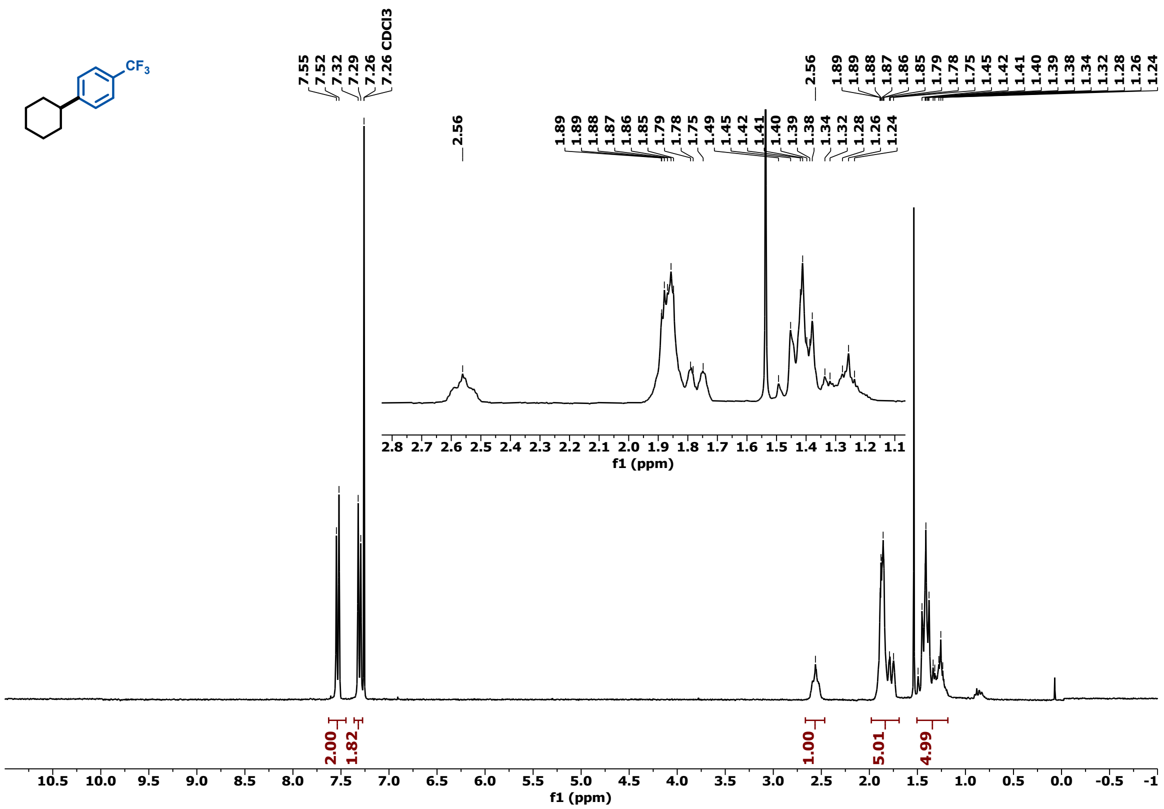

7, ^{13}C NMR (75 MHz, $CDCl_3$)

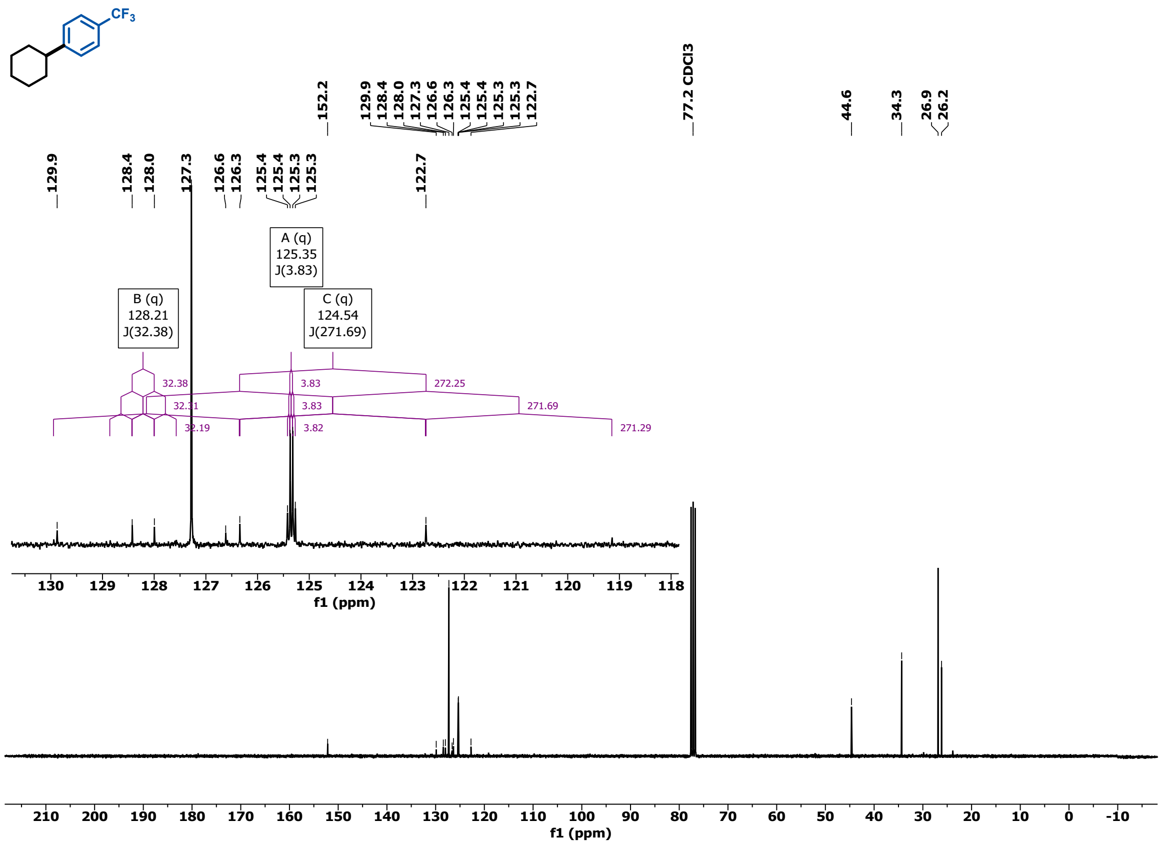

7, ^{19}F NMR (282 MHz, $CDCl_3$)

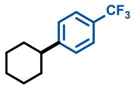

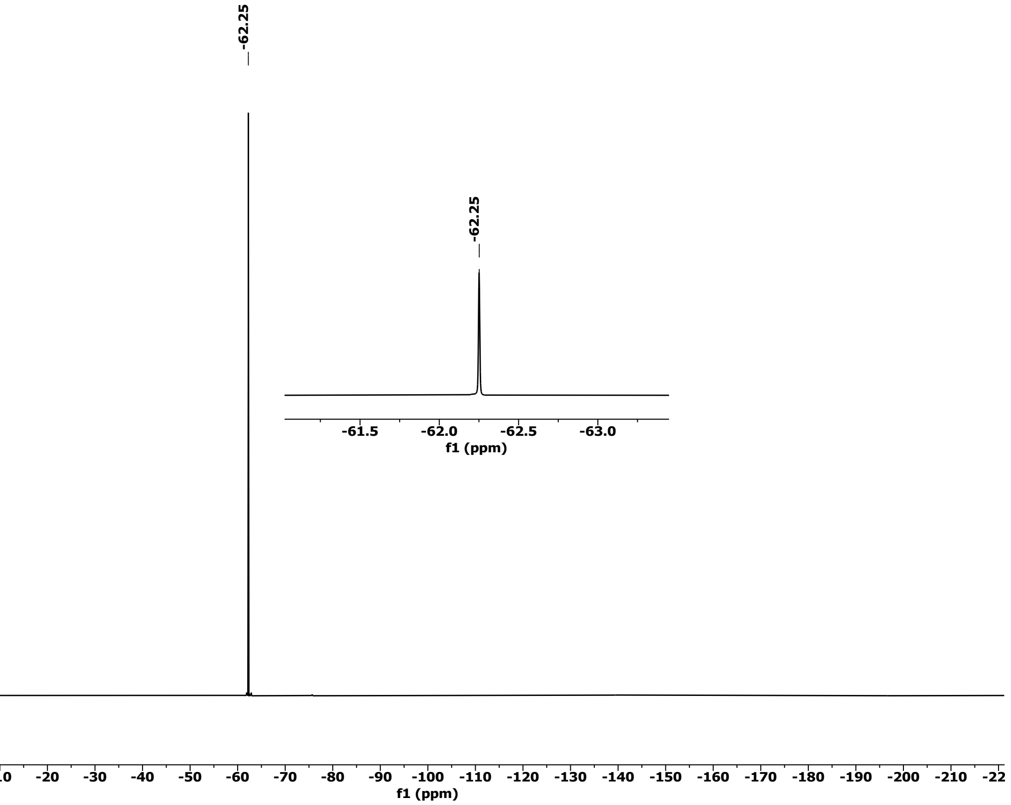

8, ^{1}H NMR (400 MHz, $CDCl_3$)

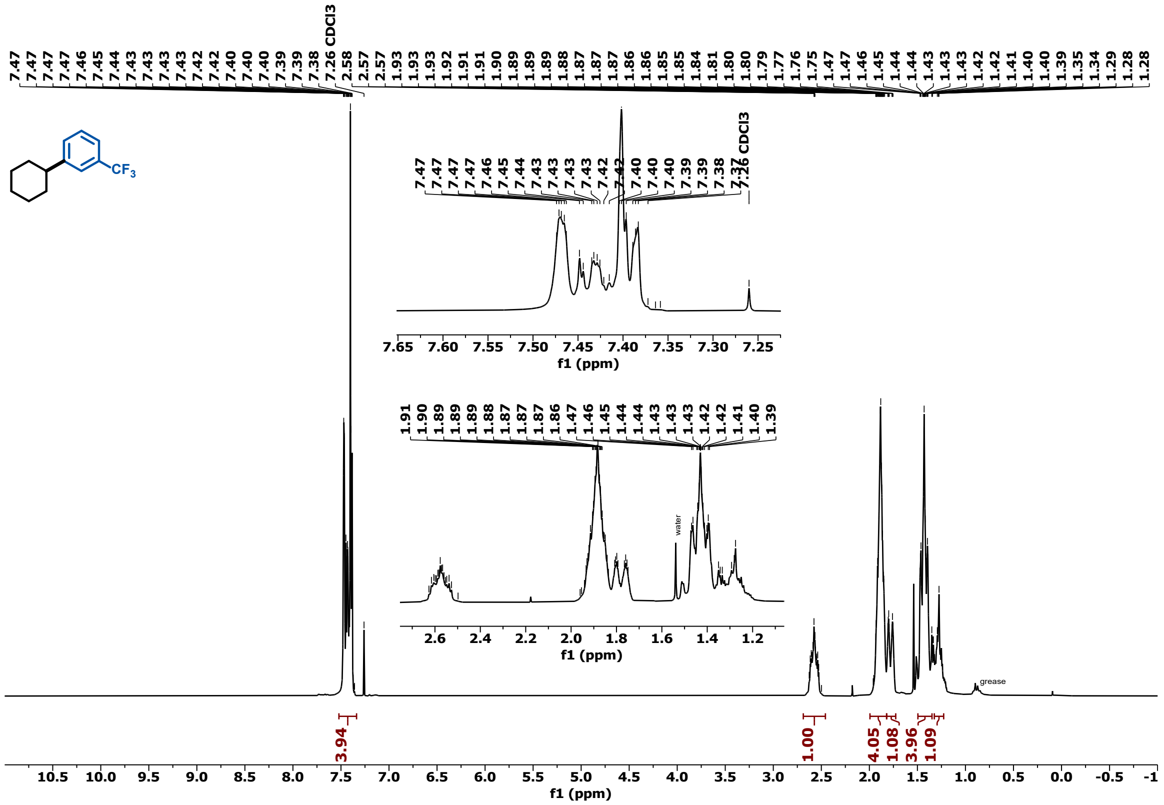

8, ^{13}C NMR (75 MHz, $CDCl_3$)

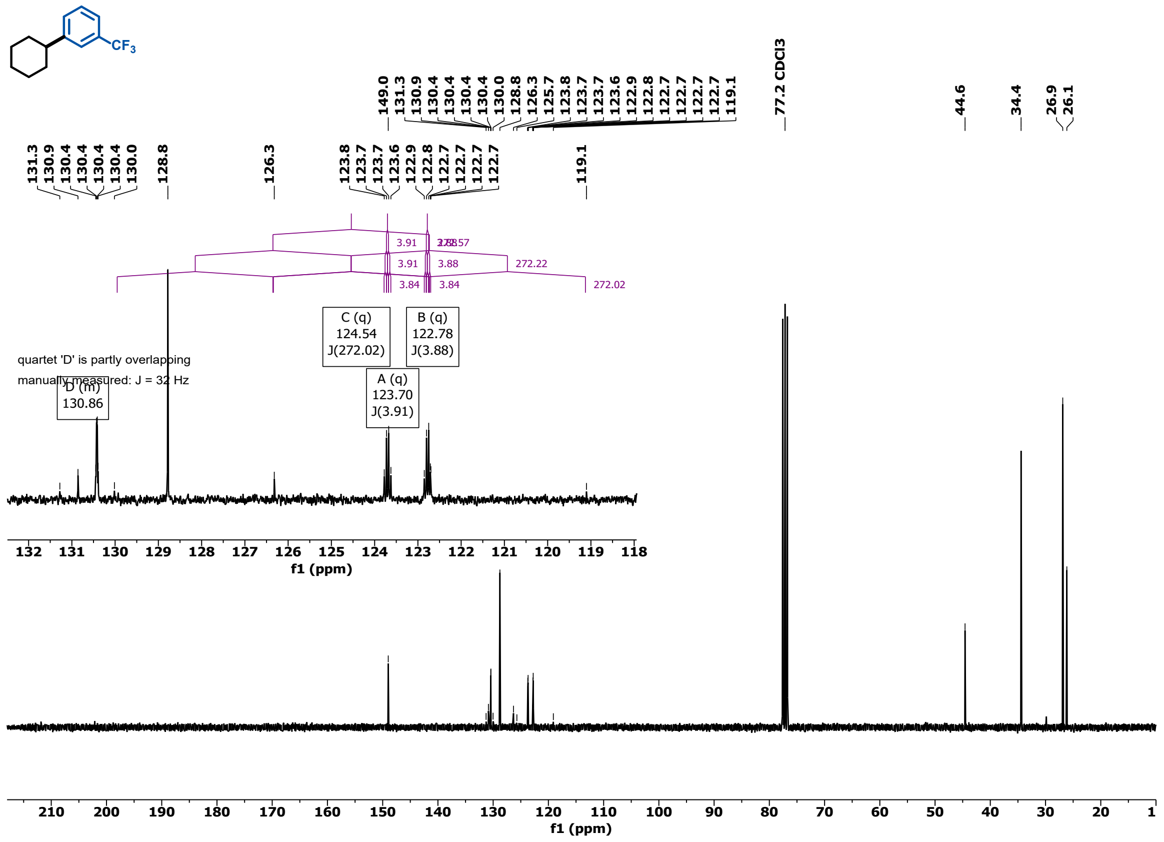

8, ^{19}F NMR (282 MHz, $CDCl_3$)

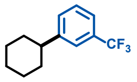

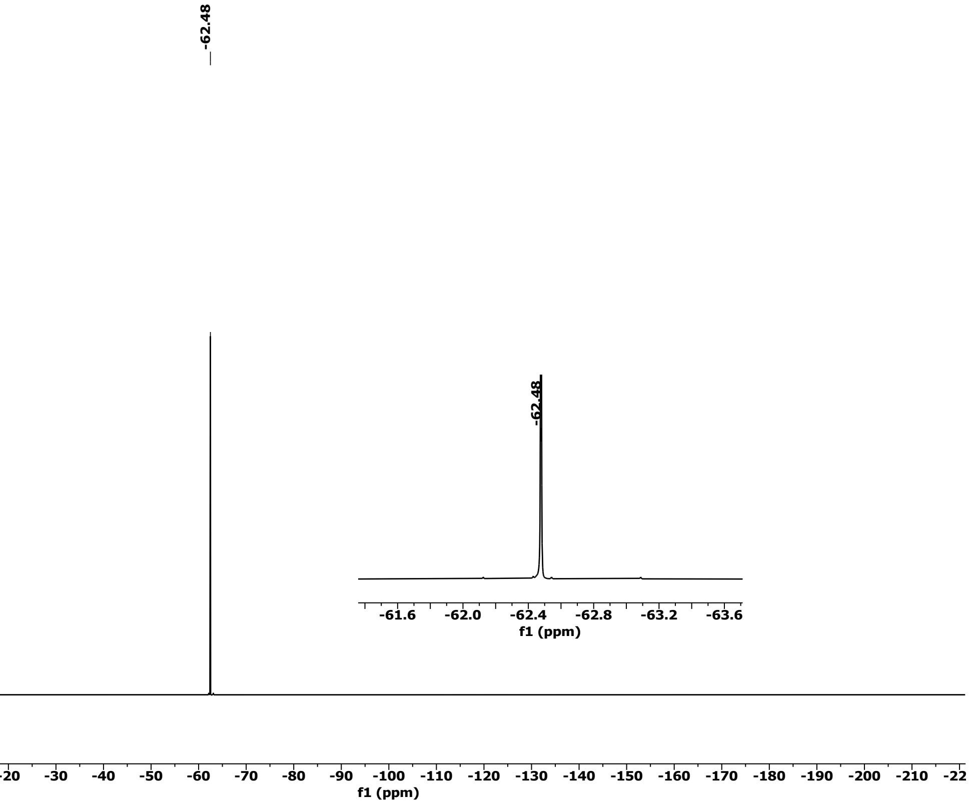

9, ^{1}H NMR (400 MHz, $CDCl_3$)

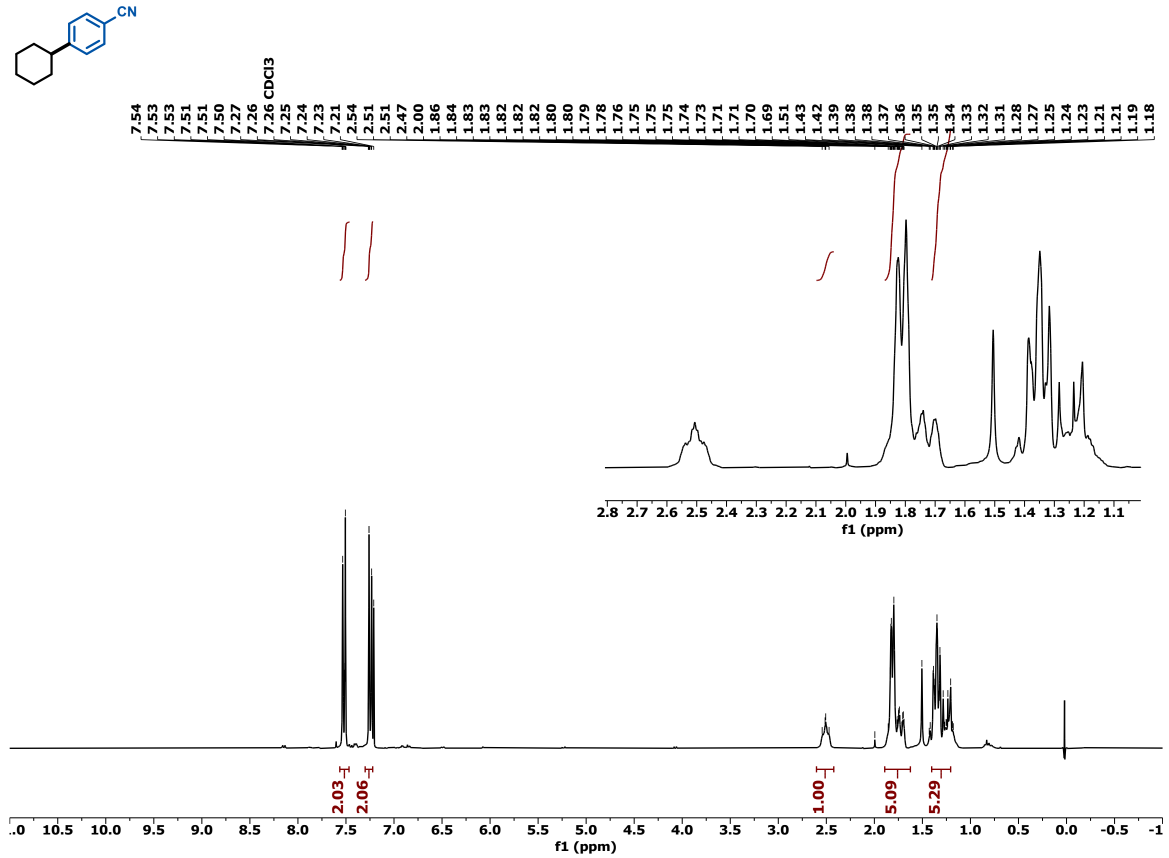

9, ^{13}C NMR (75 MHz, $CDCl_3$)

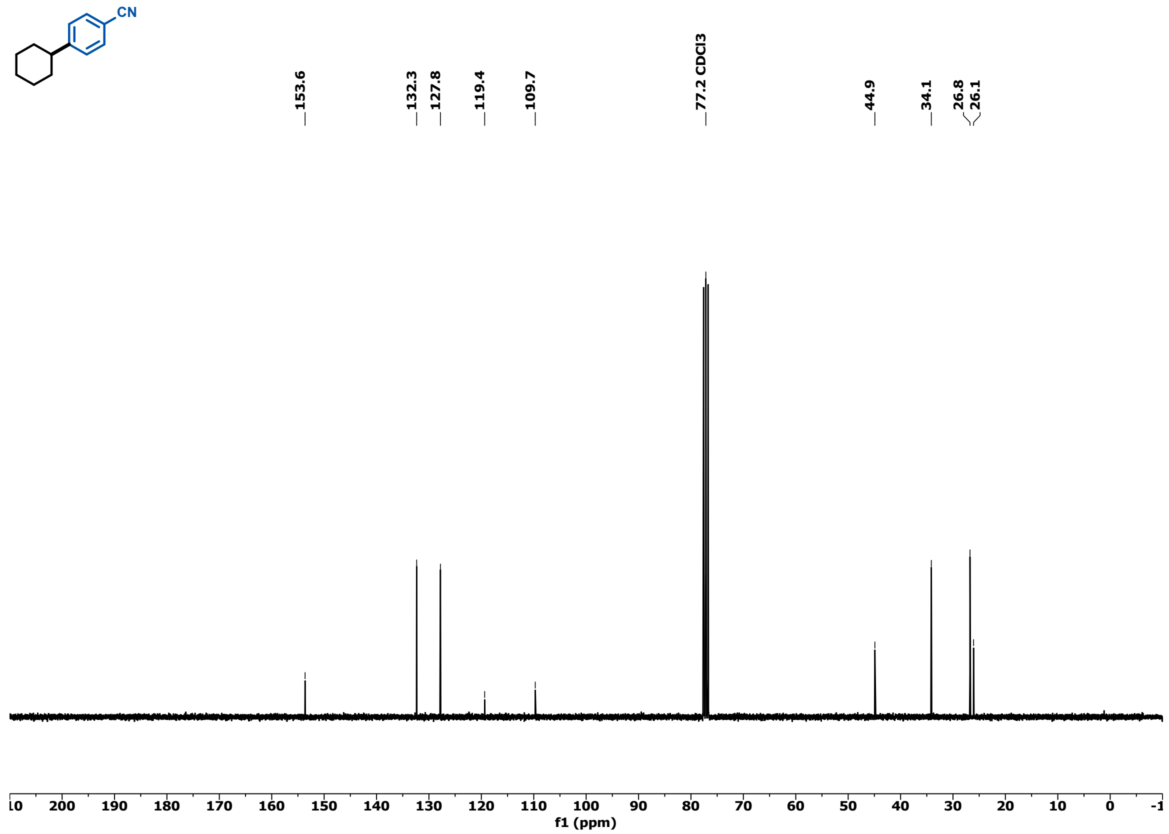

10, ^{1}H NMR (400 MHz, $CDCl_3$)

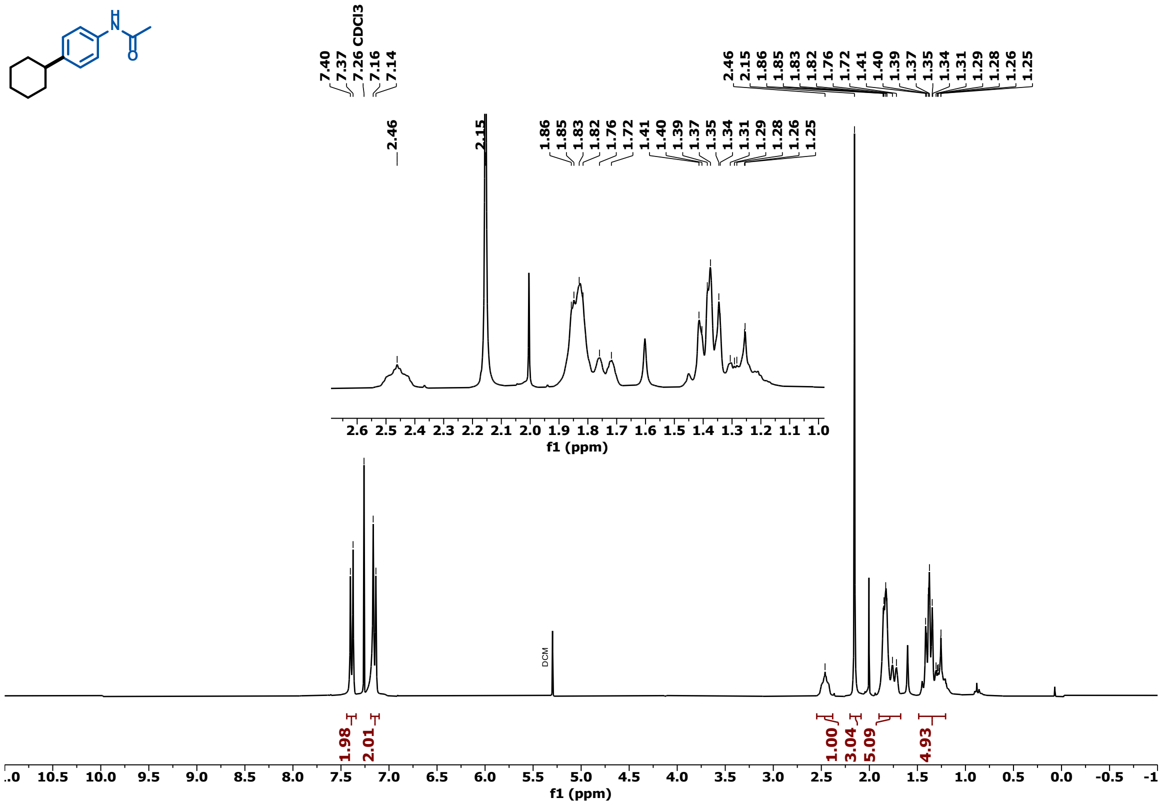

10, ^{13}C NMR (75 MHz, $CDCl_3$)

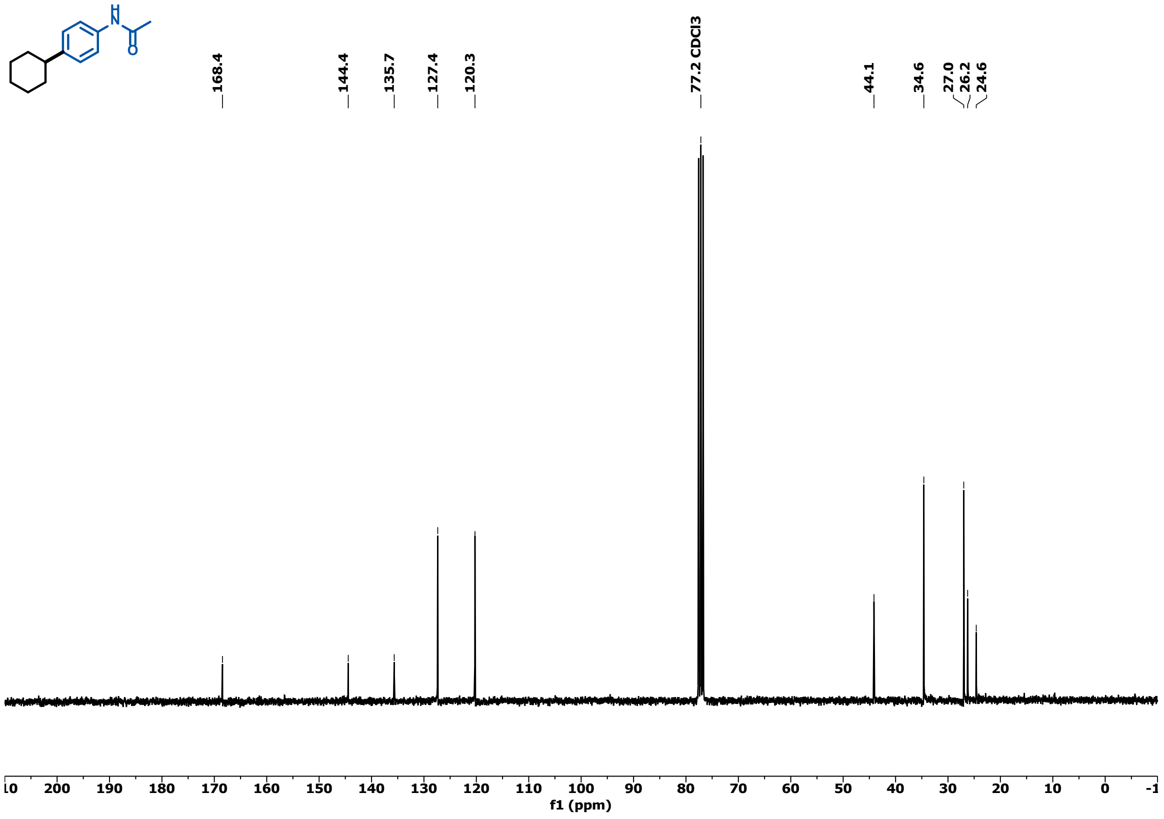

11, ^{1}H NMR (400 MHz, $CDCl_3$)

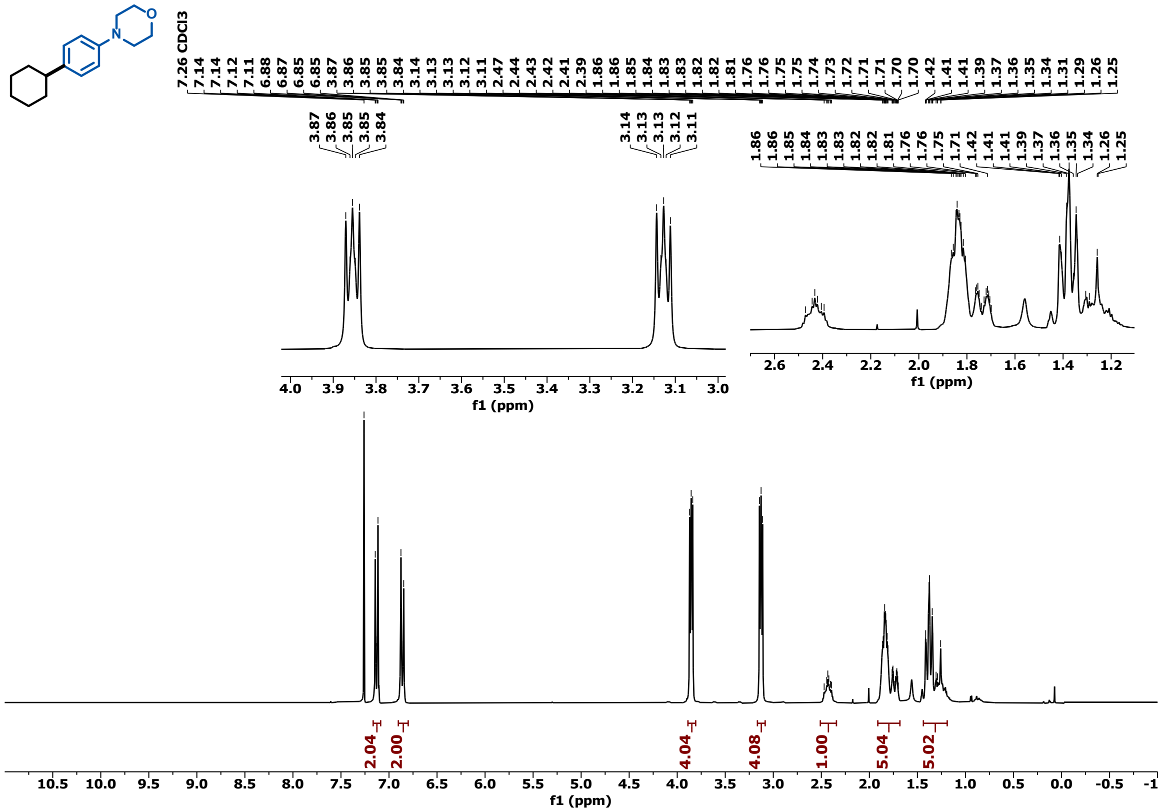

11, ^{13}C NMR (75 MHz, $CDCl_3$)

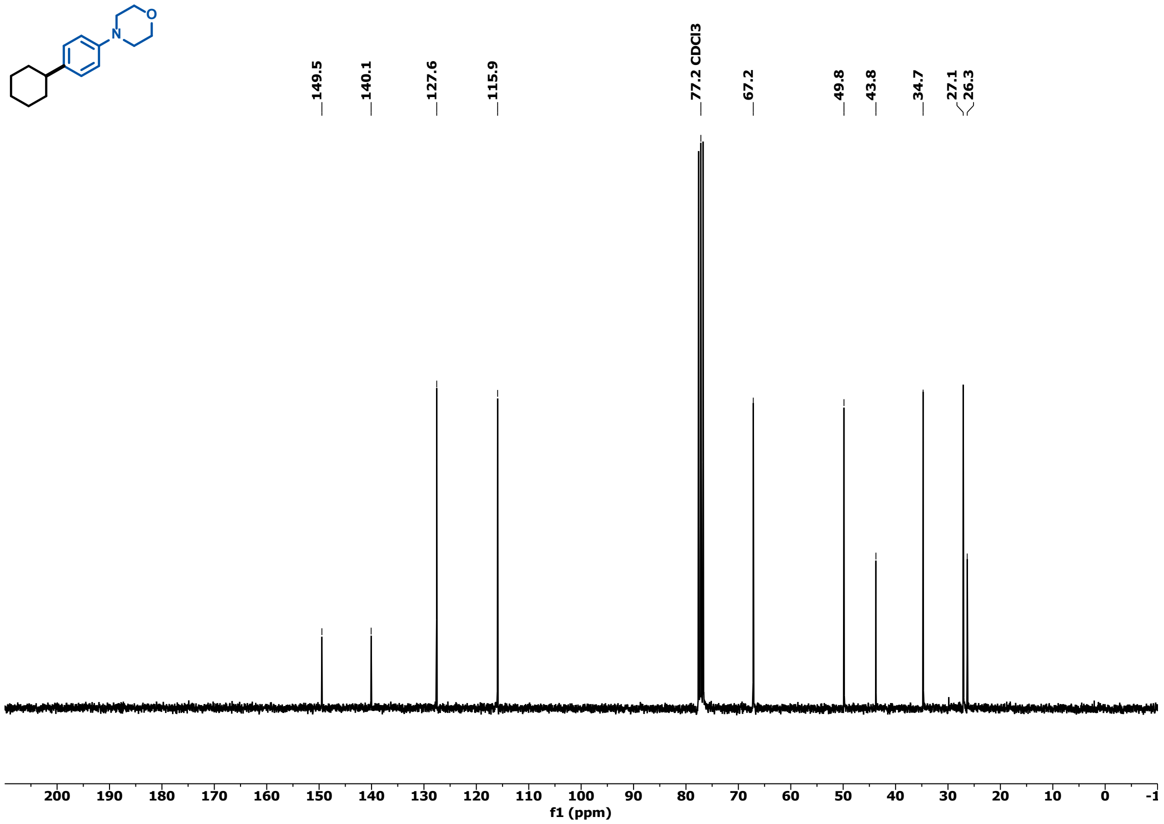

12, ^{1}H NMR (400 MHz, $CDCl_3$)

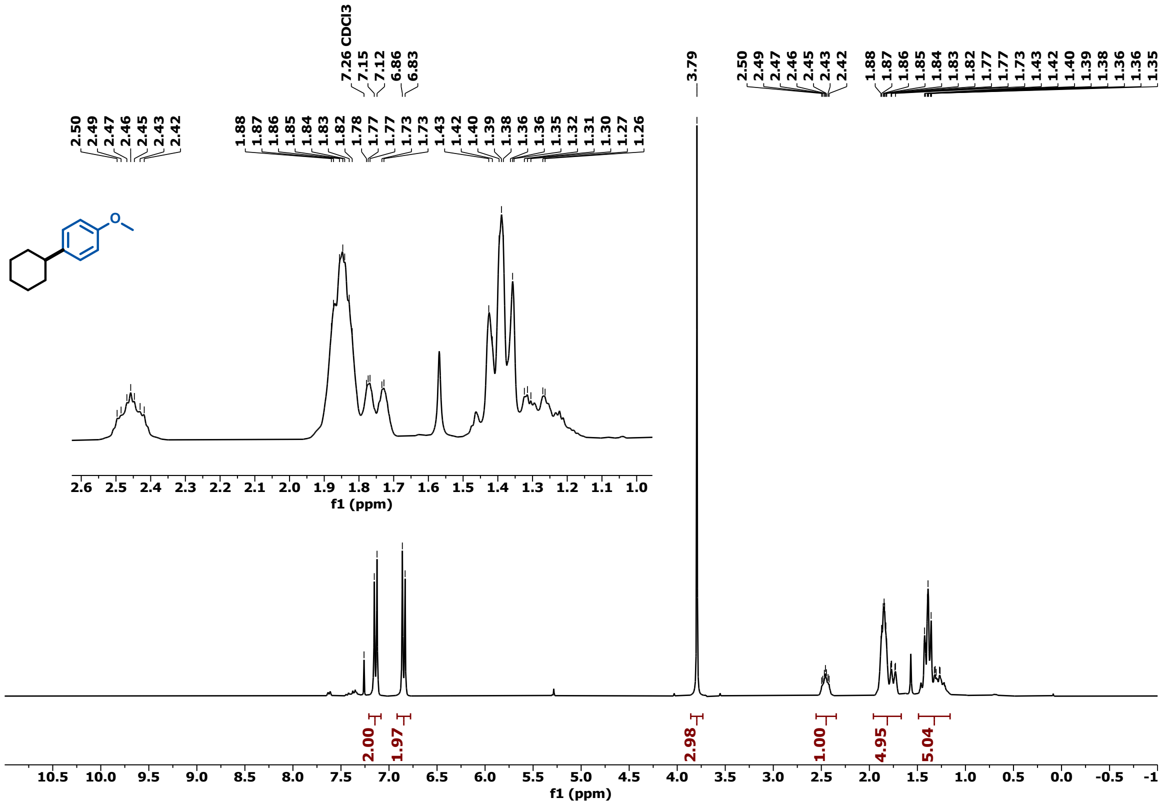

12, ^{13}C NMR (75 MHz, $CDCl_3$)

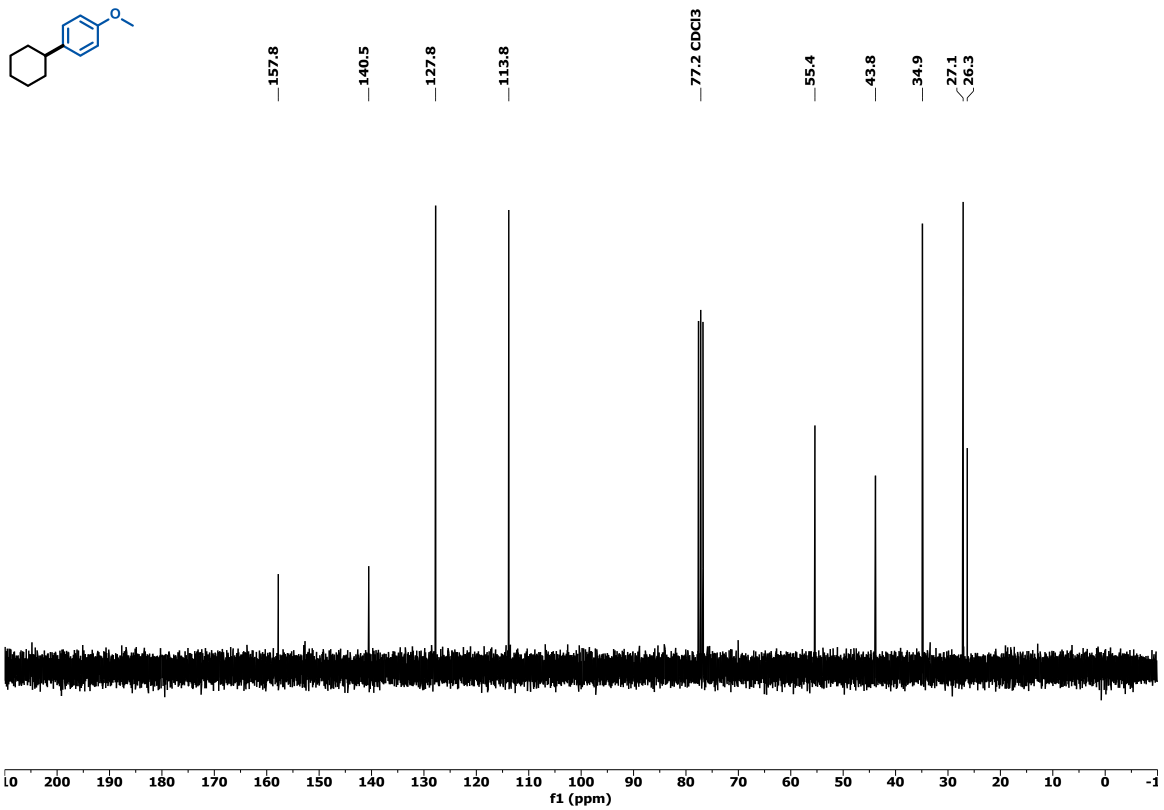

13, ^{1}H NMR (400 MHz, $CDCl_3$)

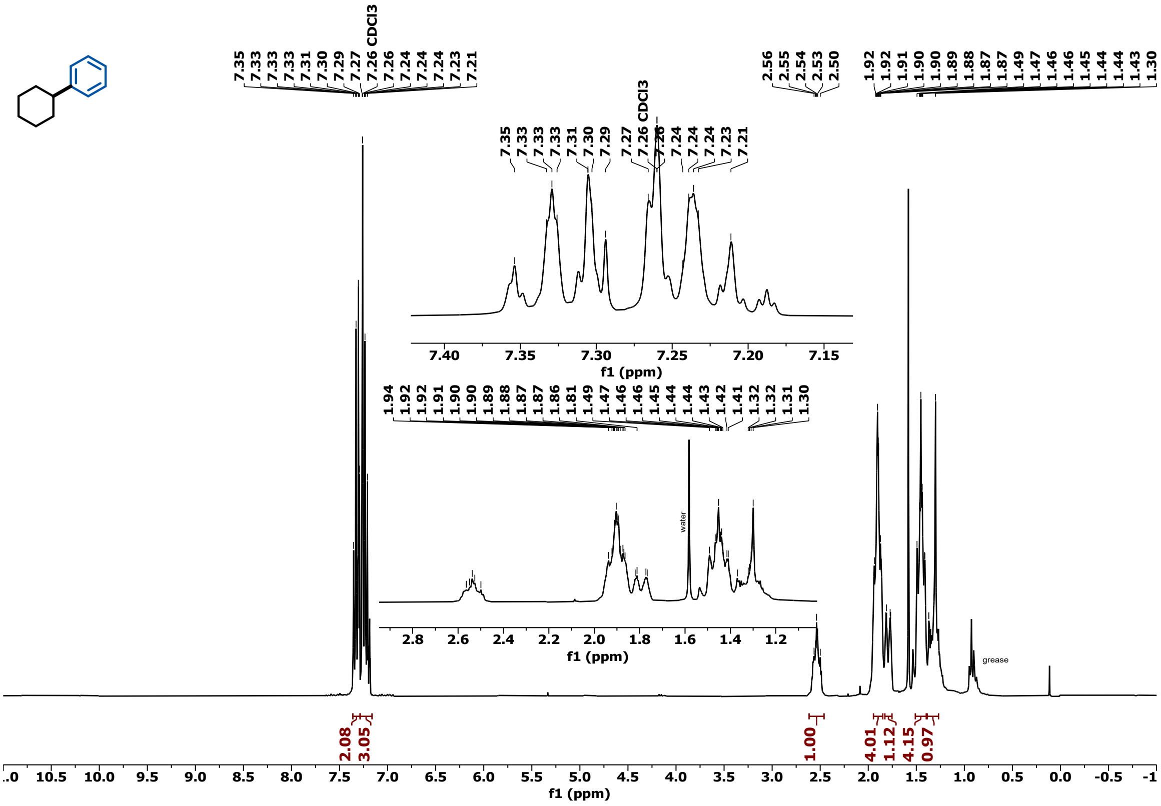

13, ^{13}C NMR (75 MHz, $CDCl_3$)

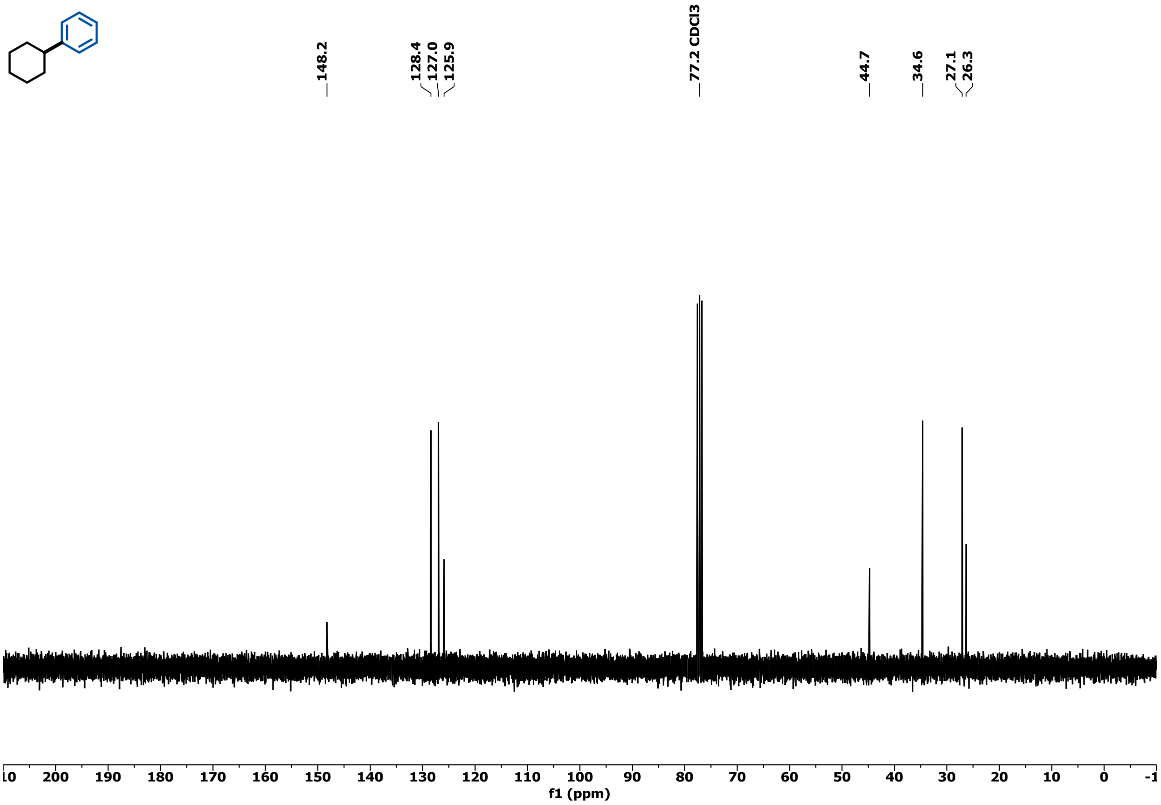

14, ^{1}H NMR (400 MHz, $CDCl_3$)

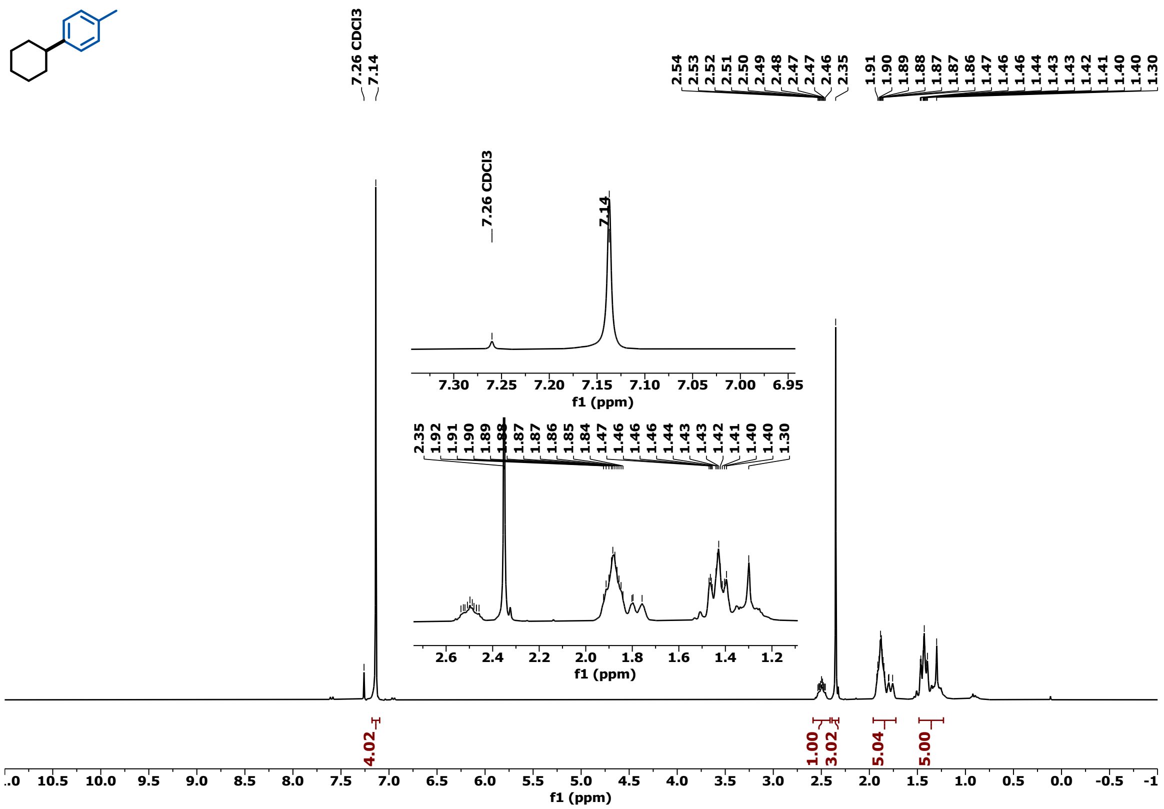

14, ^{13}C NMR (75 MHz, $CDCl_3$)

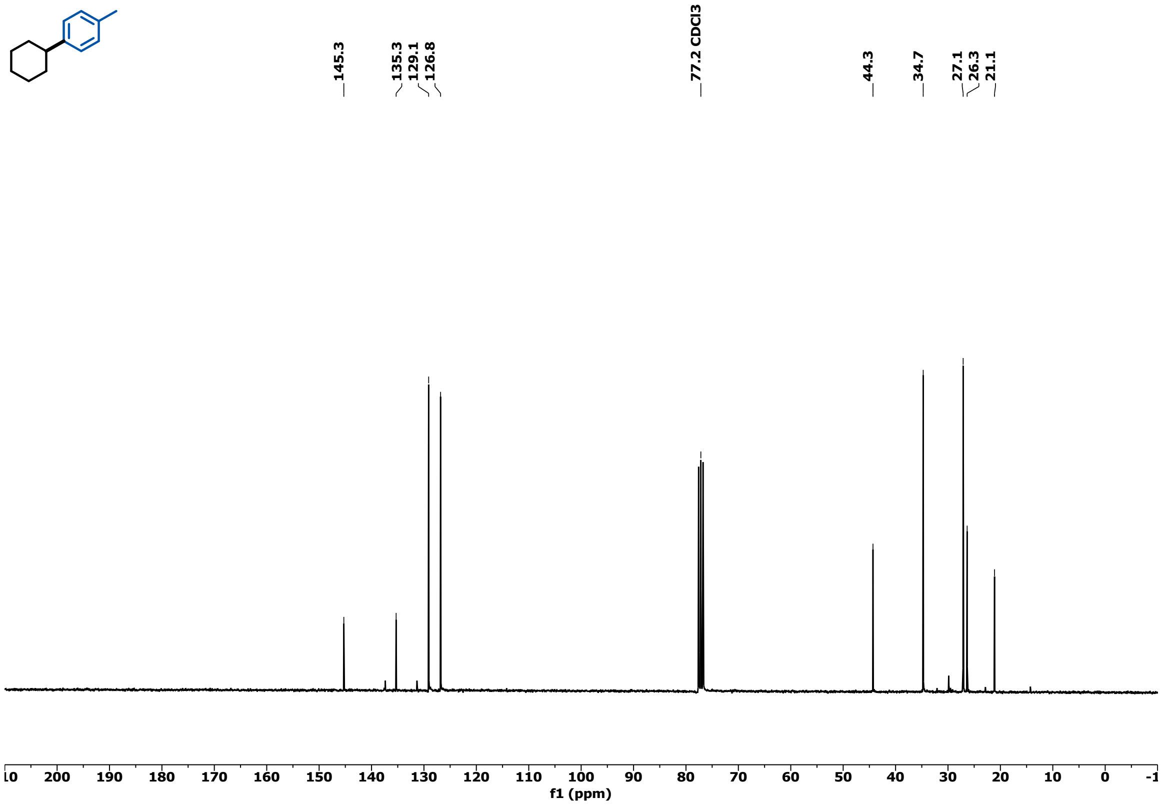

15, ^{1}H NMR (400 MHz, $CDCl_3$)

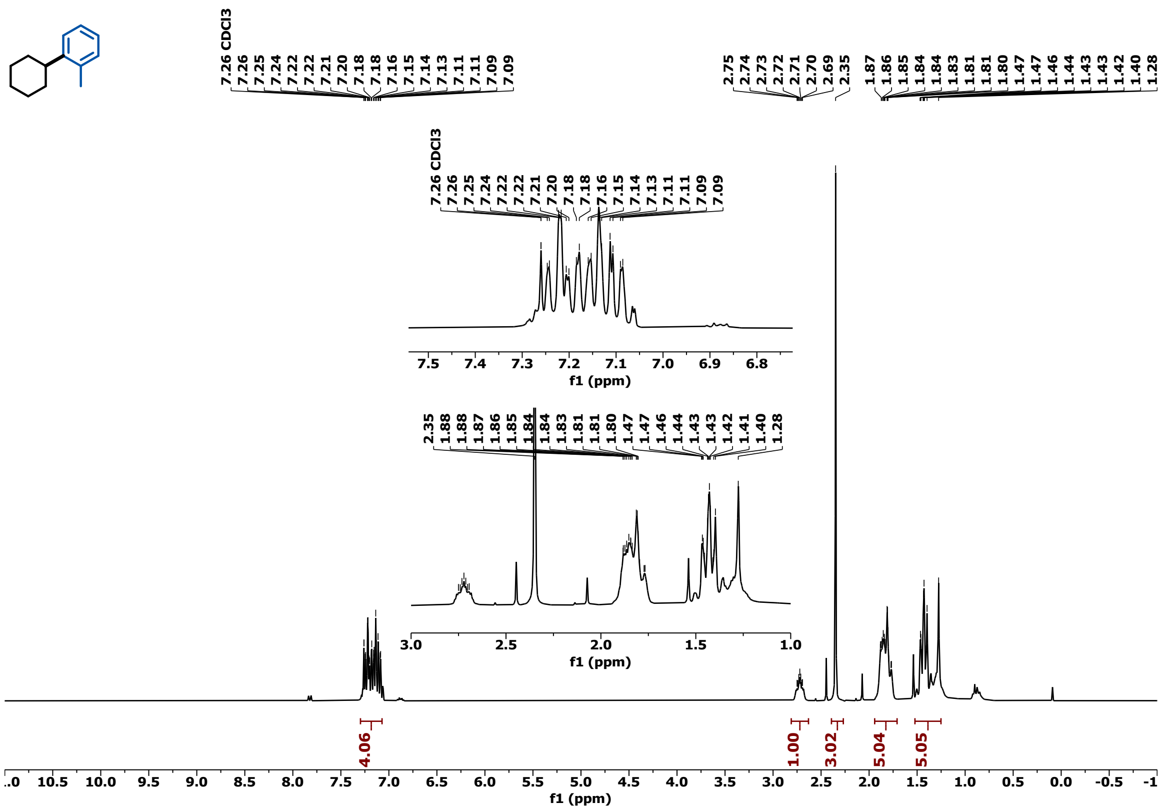

15, ^{13}C NMR (75 MHz, $CDCl_3$)

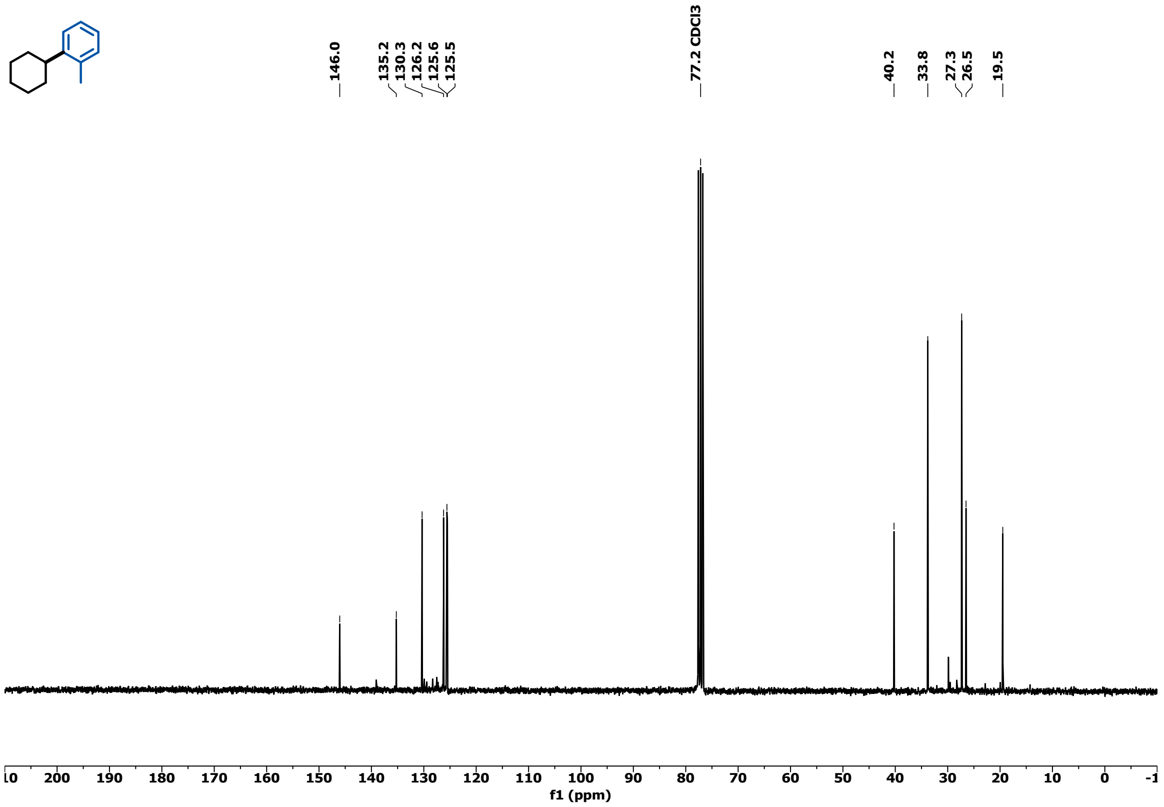

16, ^{1}H NMR (400 MHz, $CDCl_3$)

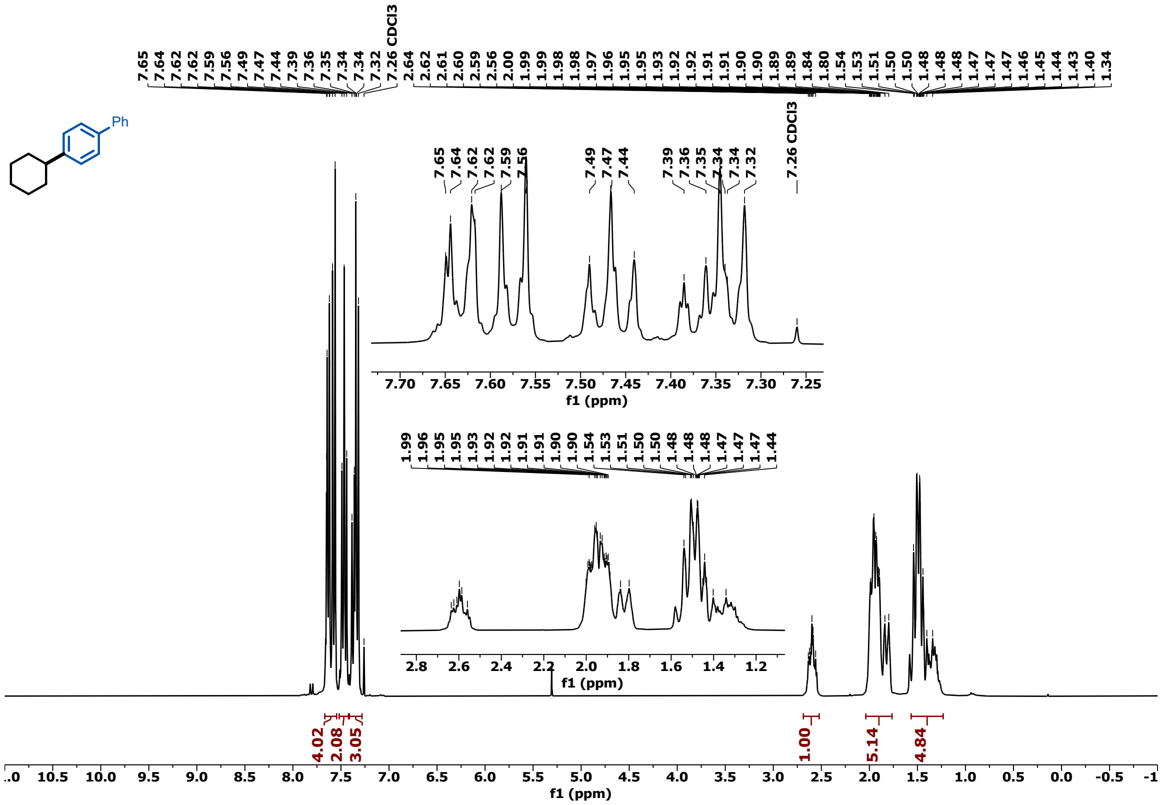

16, ^{13}C NMR (75 MHz, $CDCl_3$)

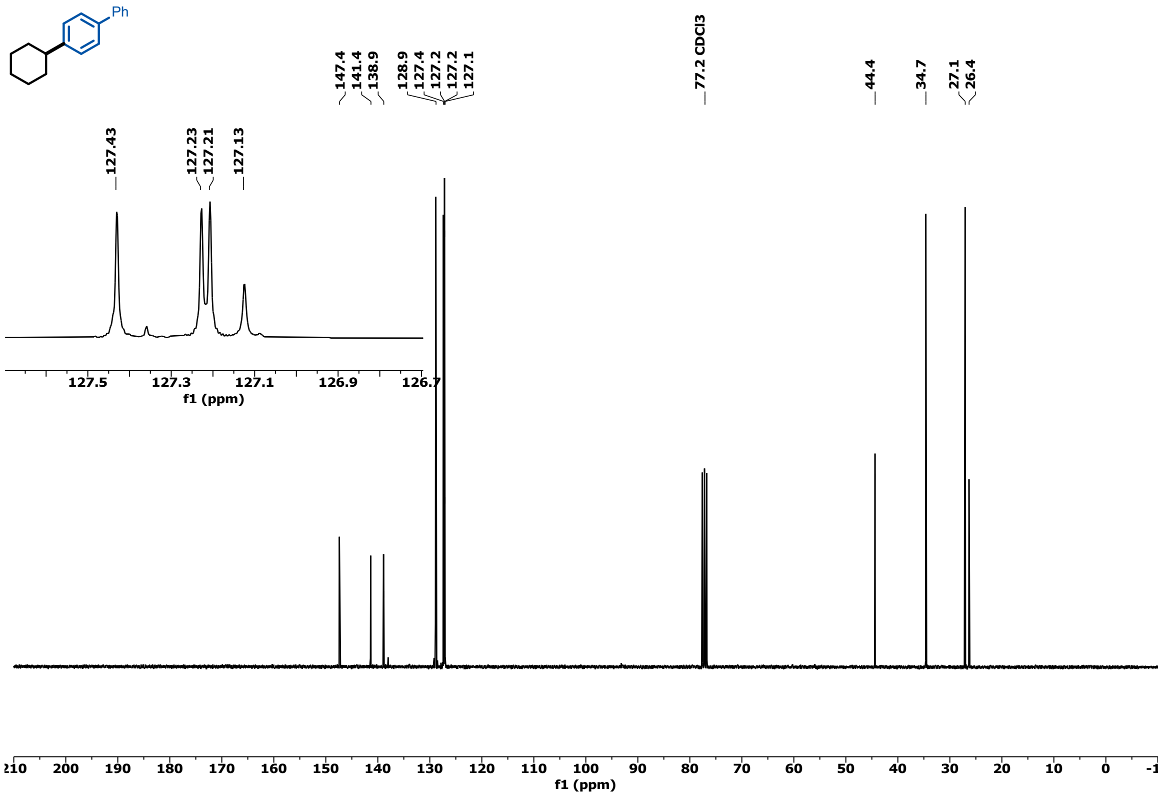

17, ^{1}H NMR (400 MHz, $CDCl_3$)

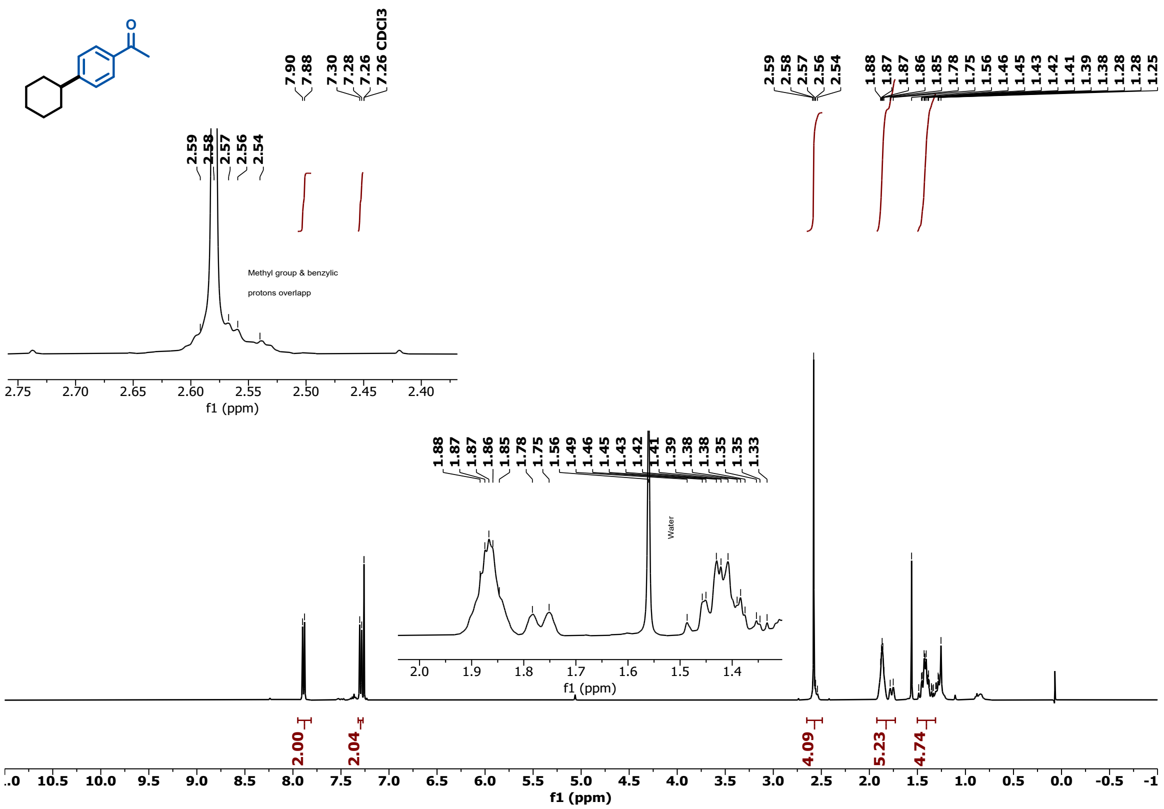

17, ^{13}C NMR (75 MHz, $CDCl_3$)

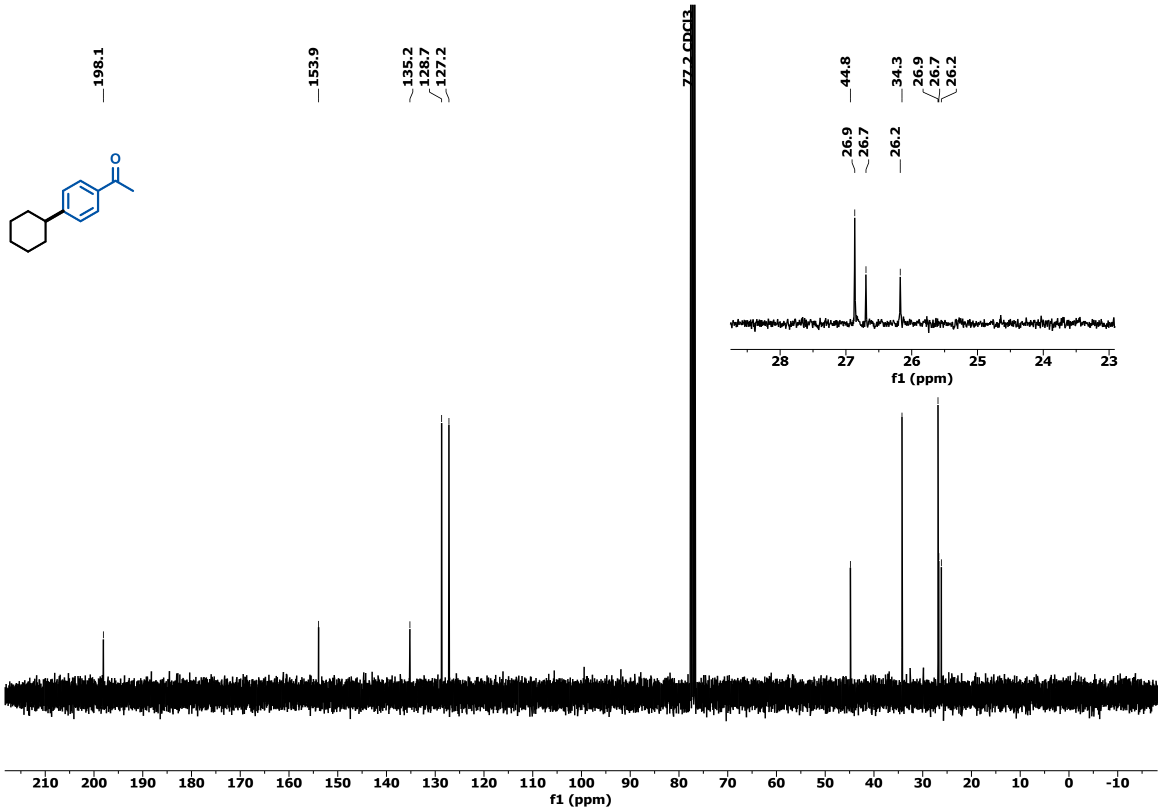

18, ^{1}H NMR (400 MHz, $CDCl_3$)

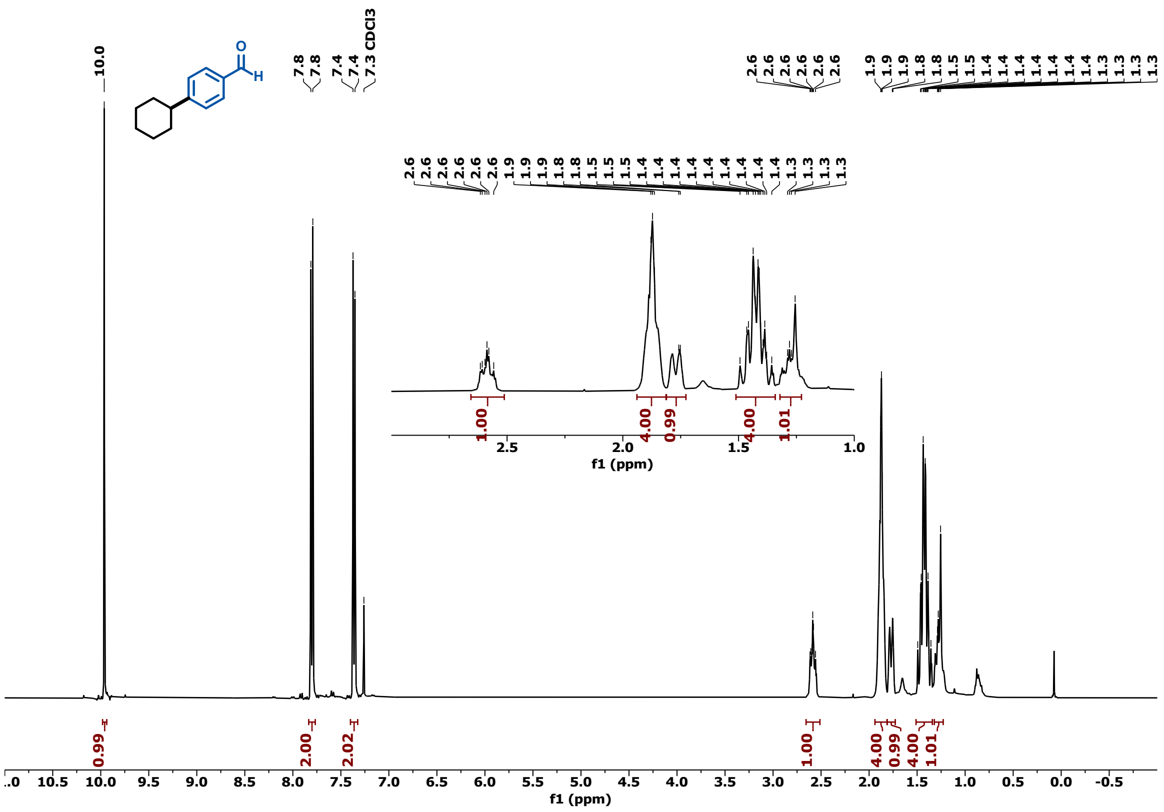

18, ^{13}C NMR (75 MHz, $CDCl_3$)

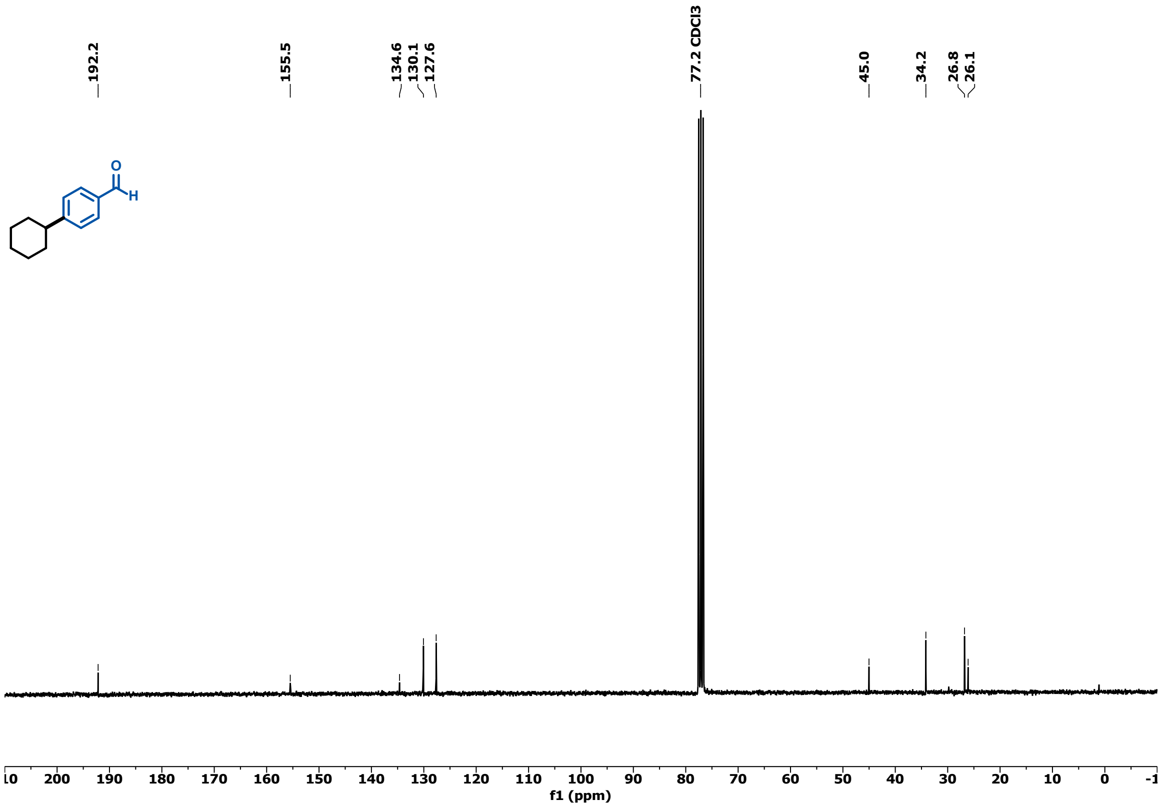

19, ^{1}H NMR (400 MHz, $CDCl_3$)

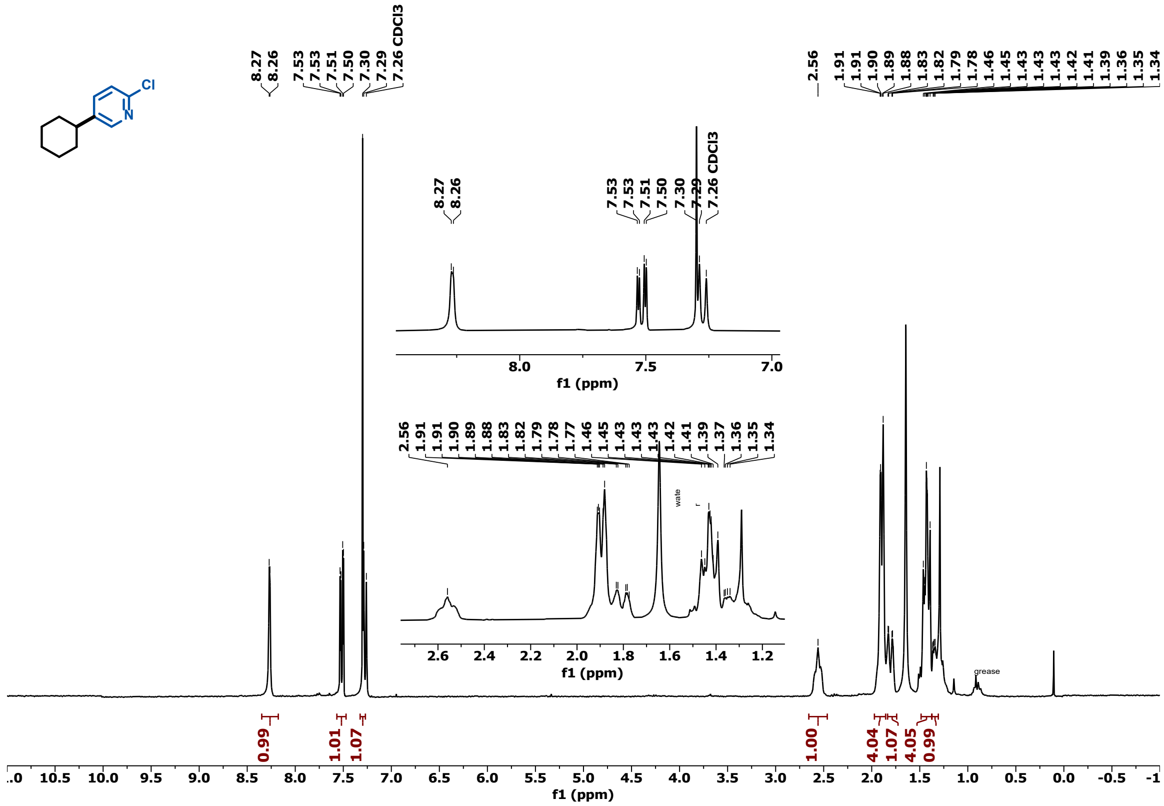

19, ^{13}C NMR (75 MHz, $CDCl_3$)

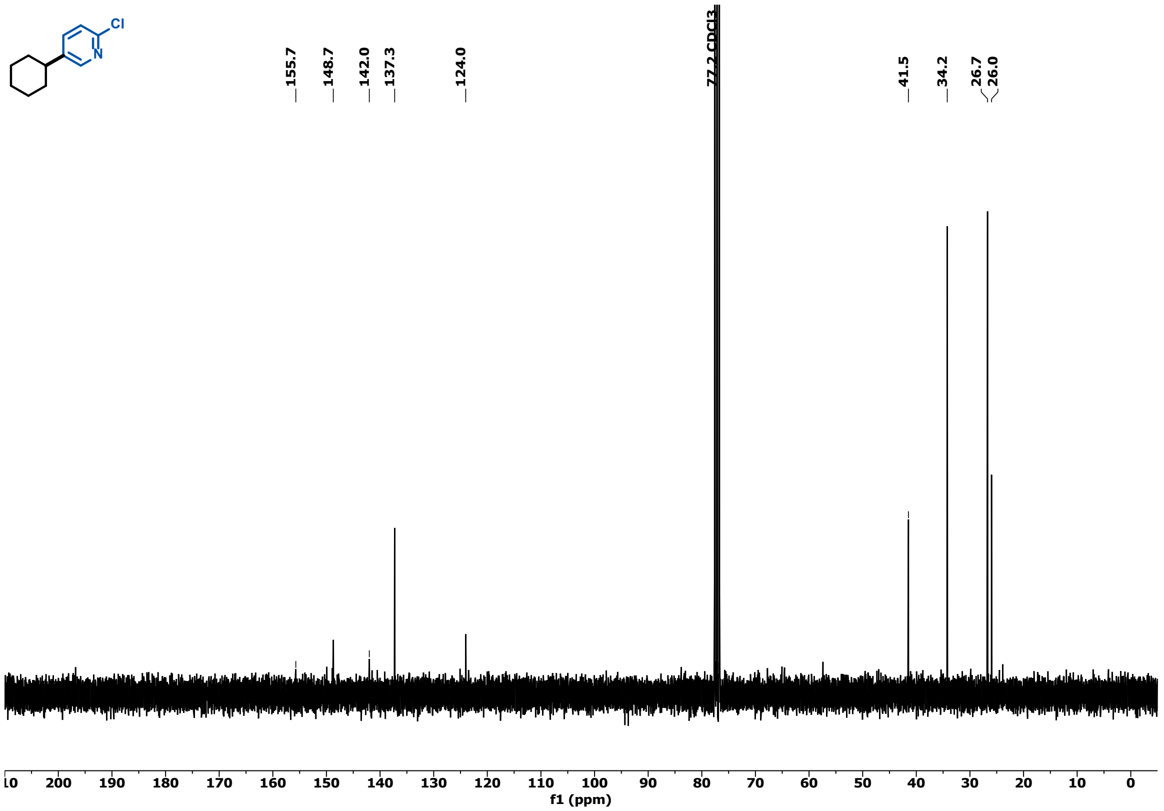

20, ^{1}H NMR (400 MHz, $CDCl_3$)

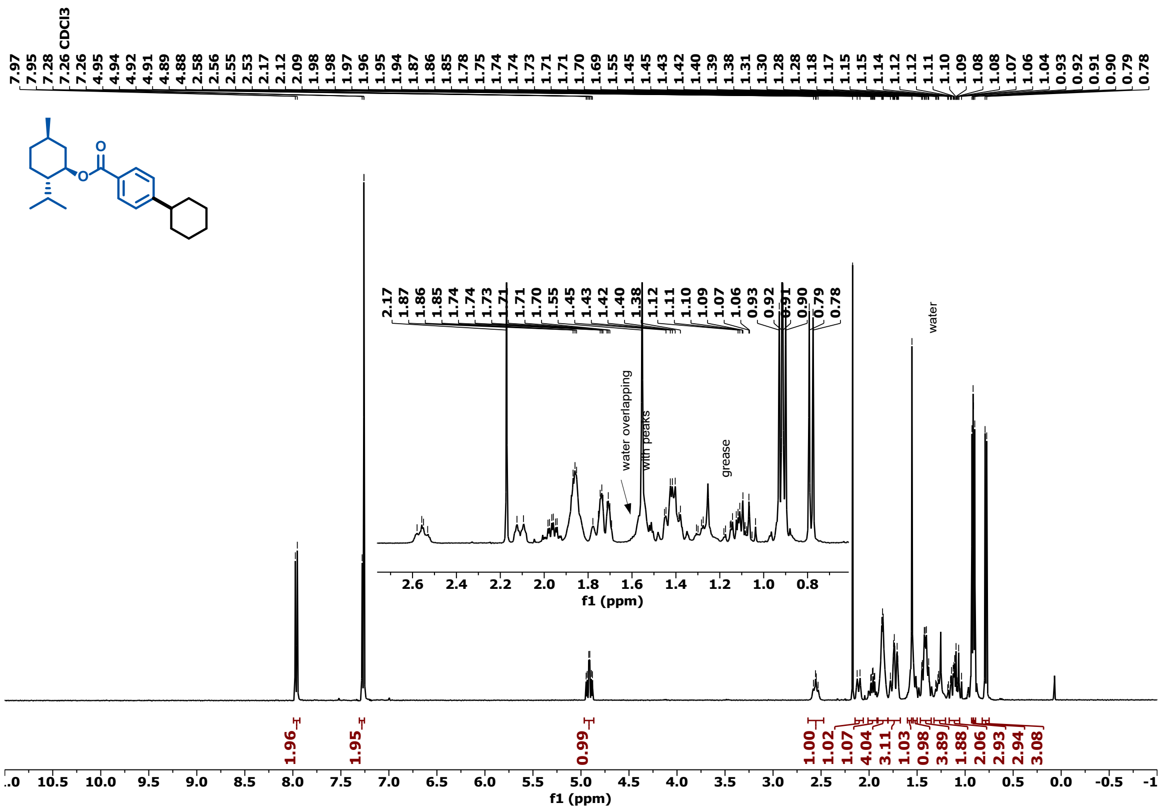

20, ^{13}C NMR (75 MHz, $CDCl_3$)

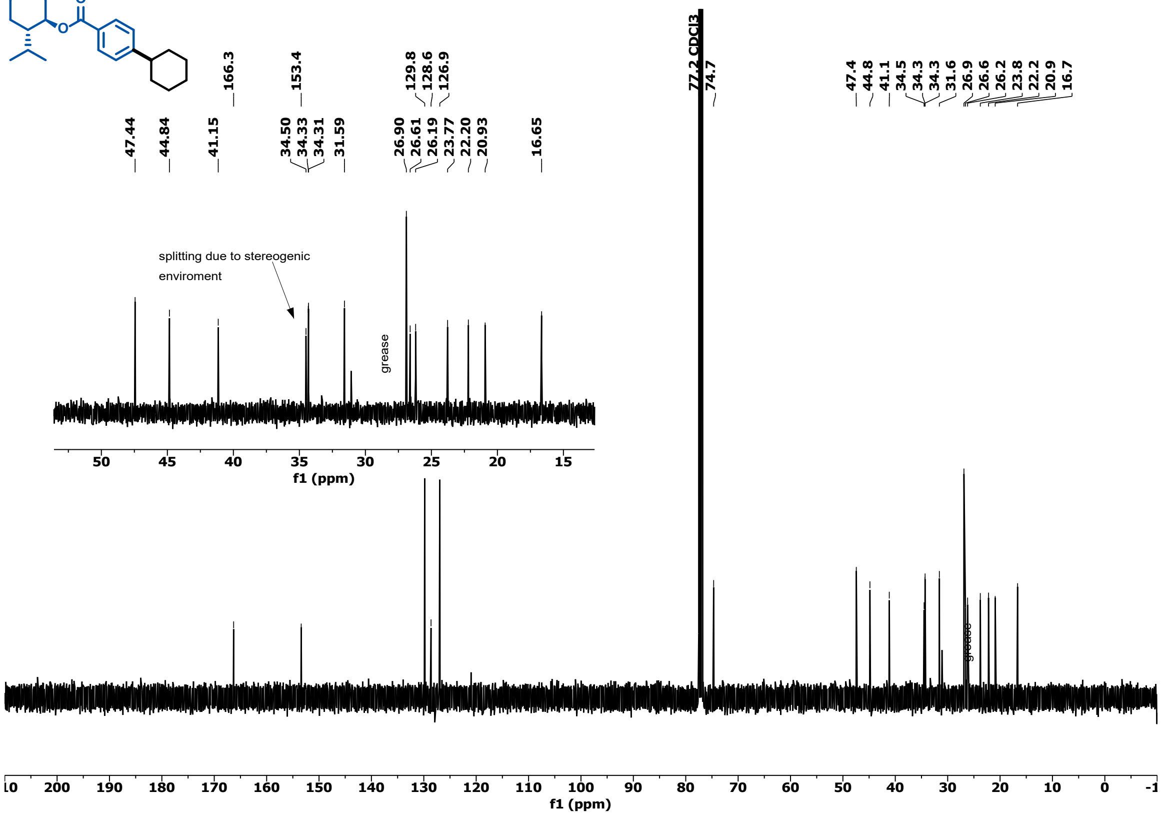

21, ^{1}H NMR (400 MHz, $CDCl_3$)

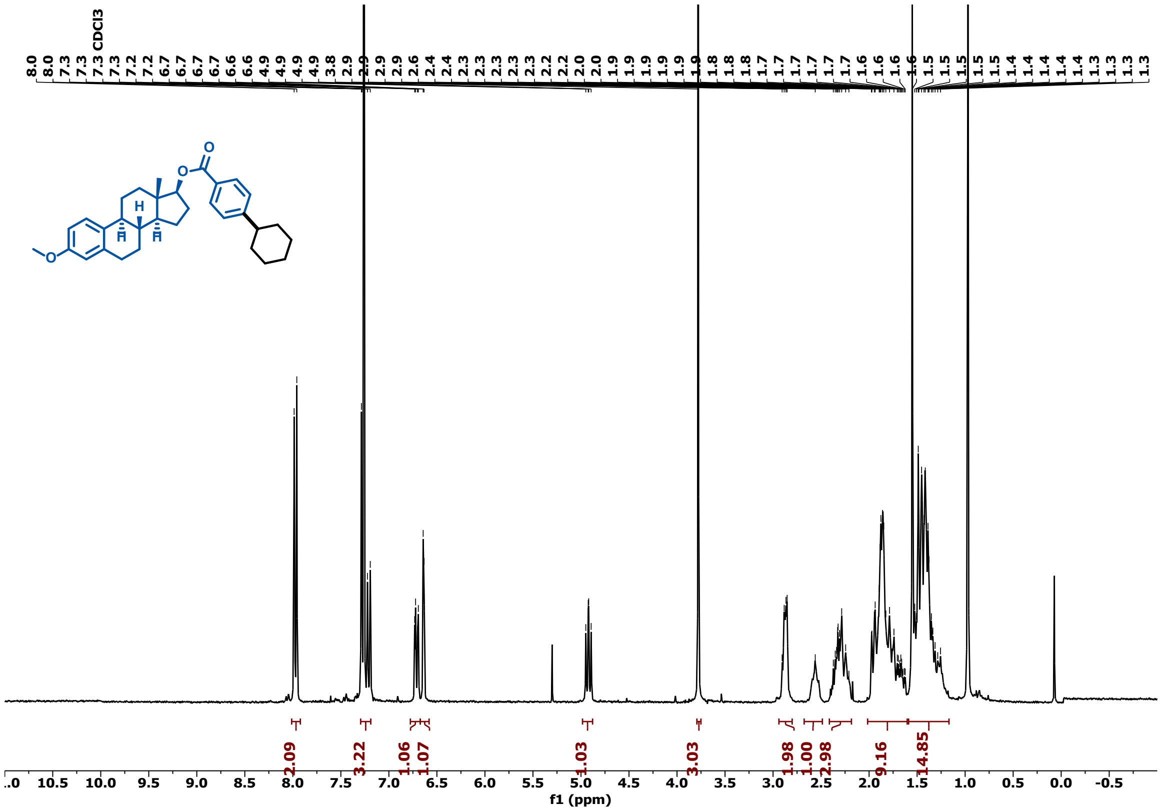

21, ^{13}C NMR (75 MHz, $CDCl_3$)

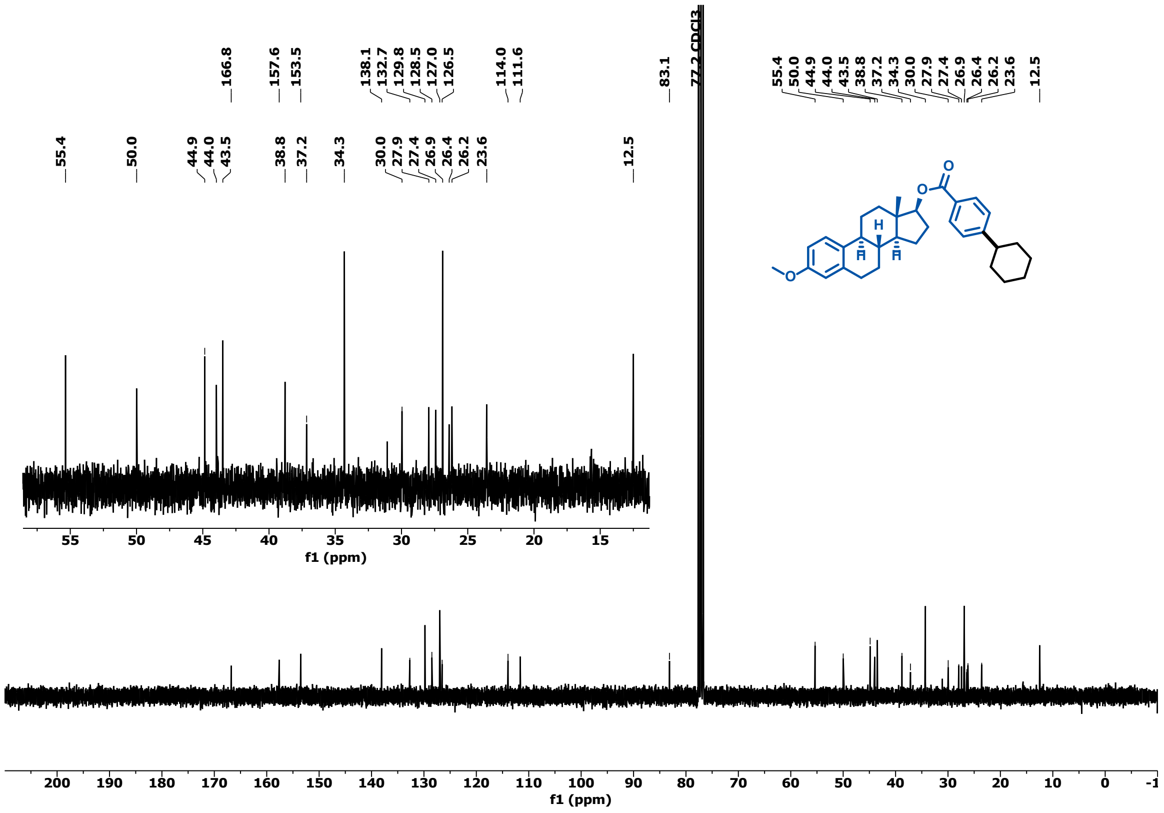

22, ^{1}H NMR (400 MHz, $CDCl_3$)

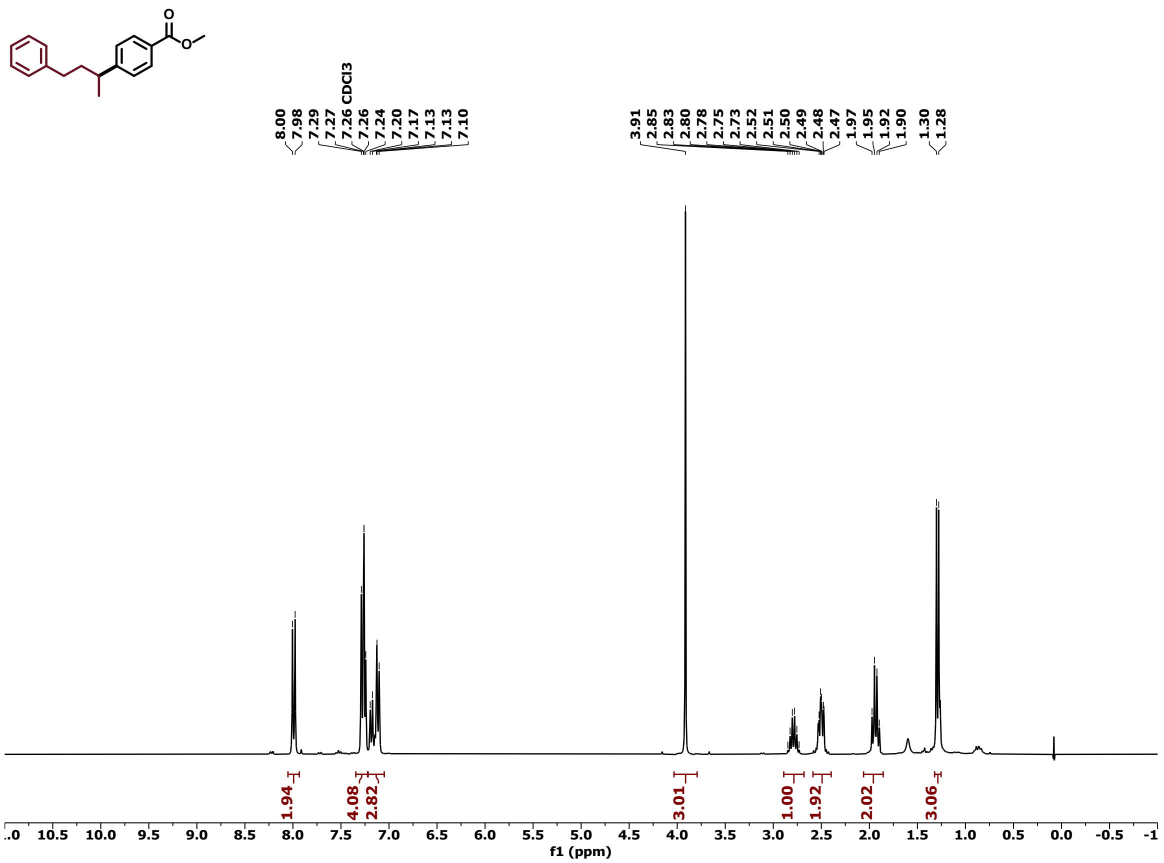

22, ^{13}C NMR (75 MHz, $CDCl_3$)

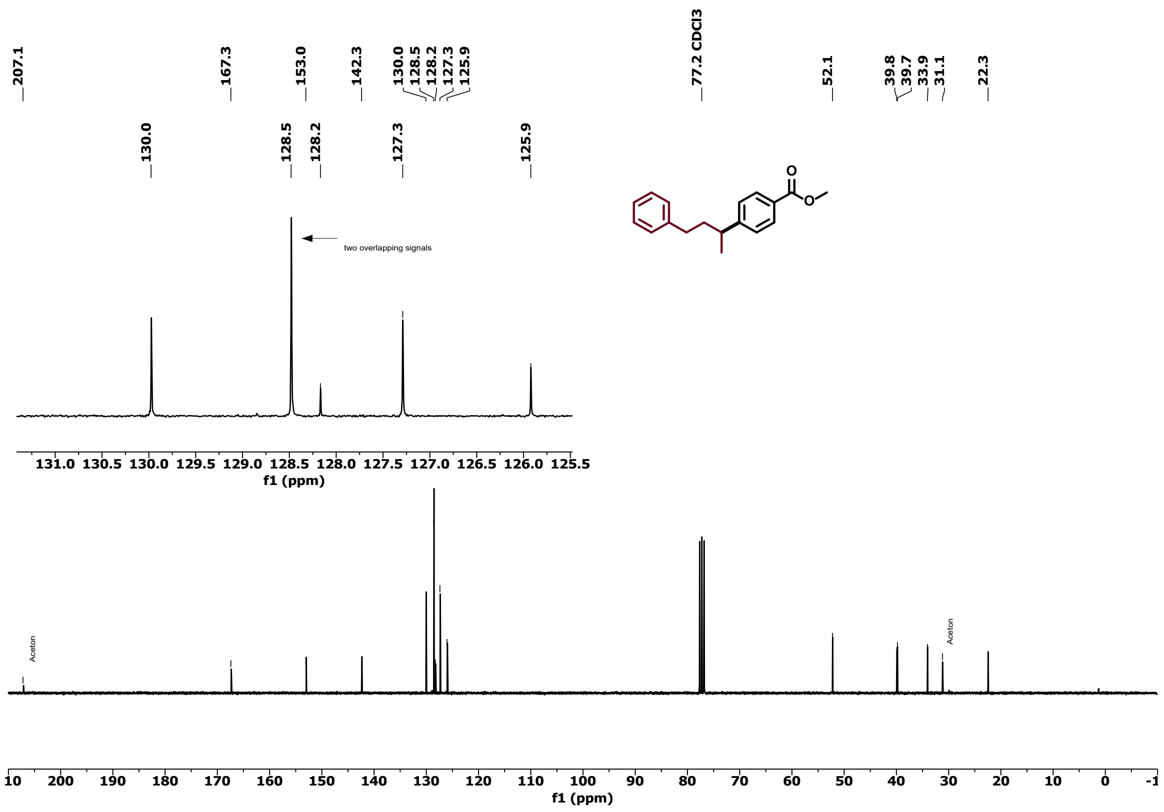

23, ^{1}H NMR (400 MHz, $CDCl_3$)

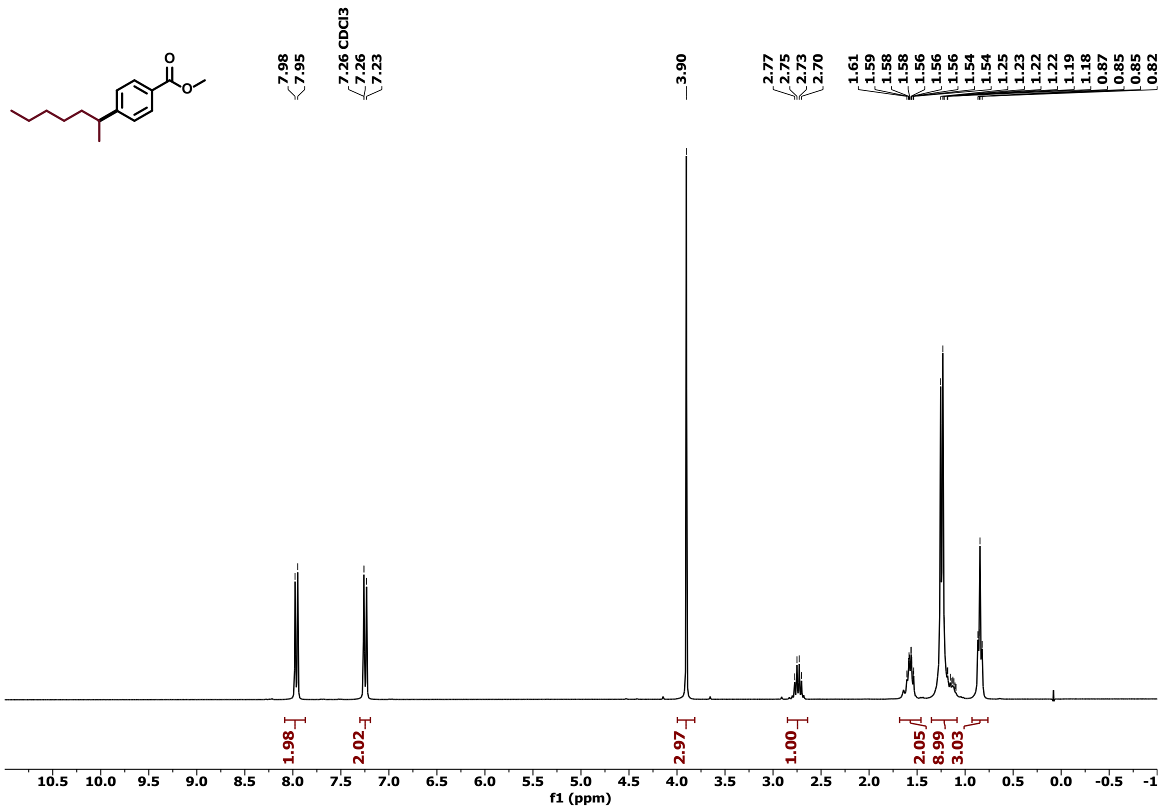

23, ^{13}C NMR (75 MHz, $CDCl_3$)

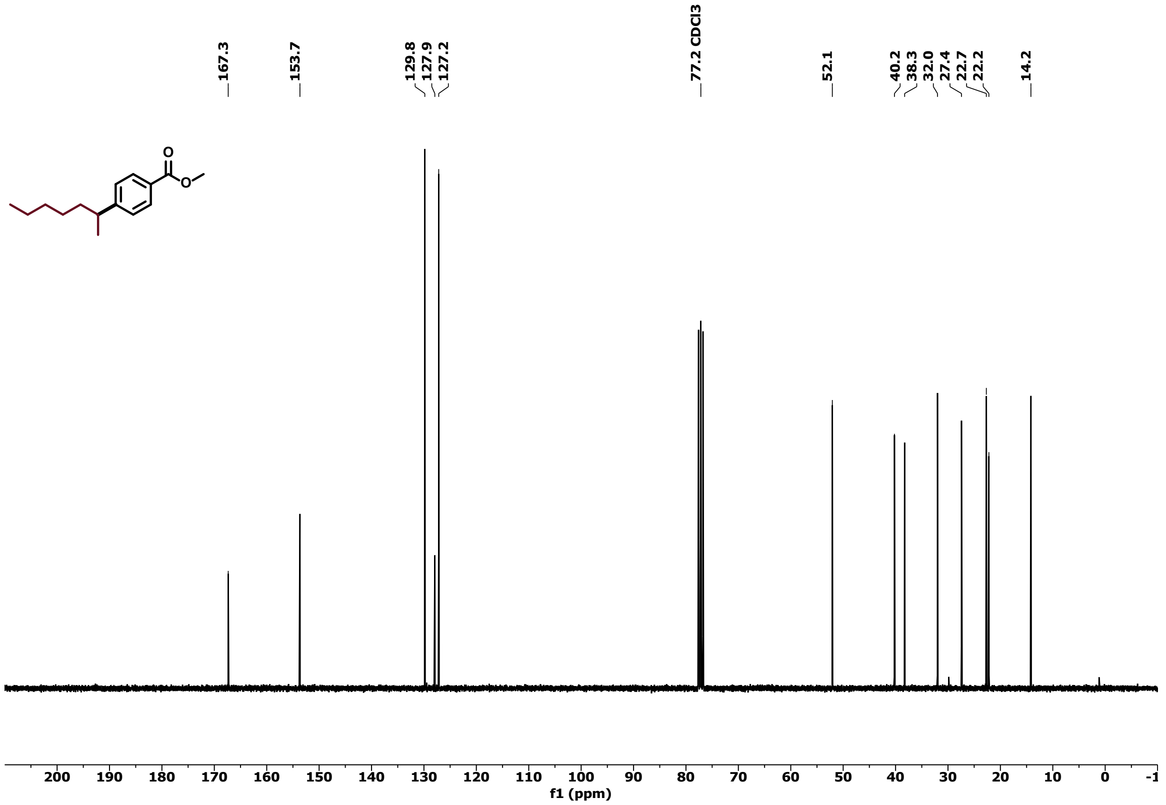

24, ^{1}H NMR (400 MHz, $CDCl_3$)

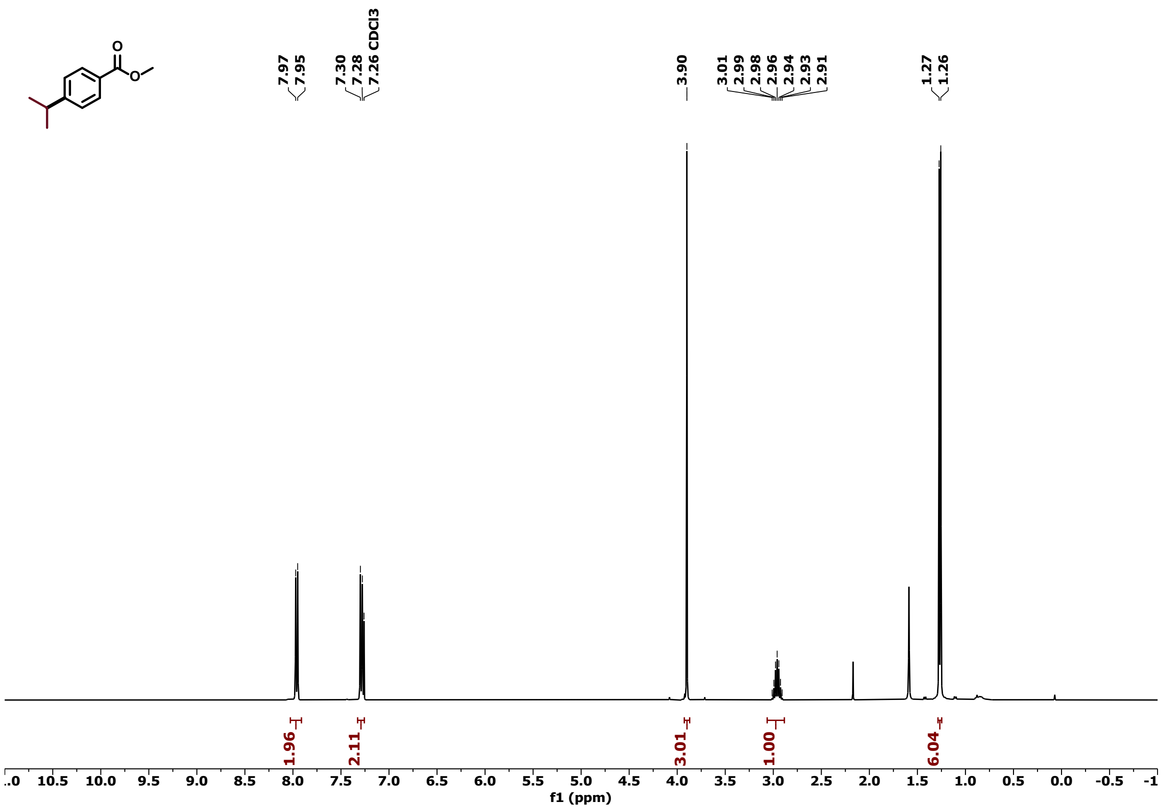

24, ^{13}C NMR (75 MHz, $CDCl_3$)

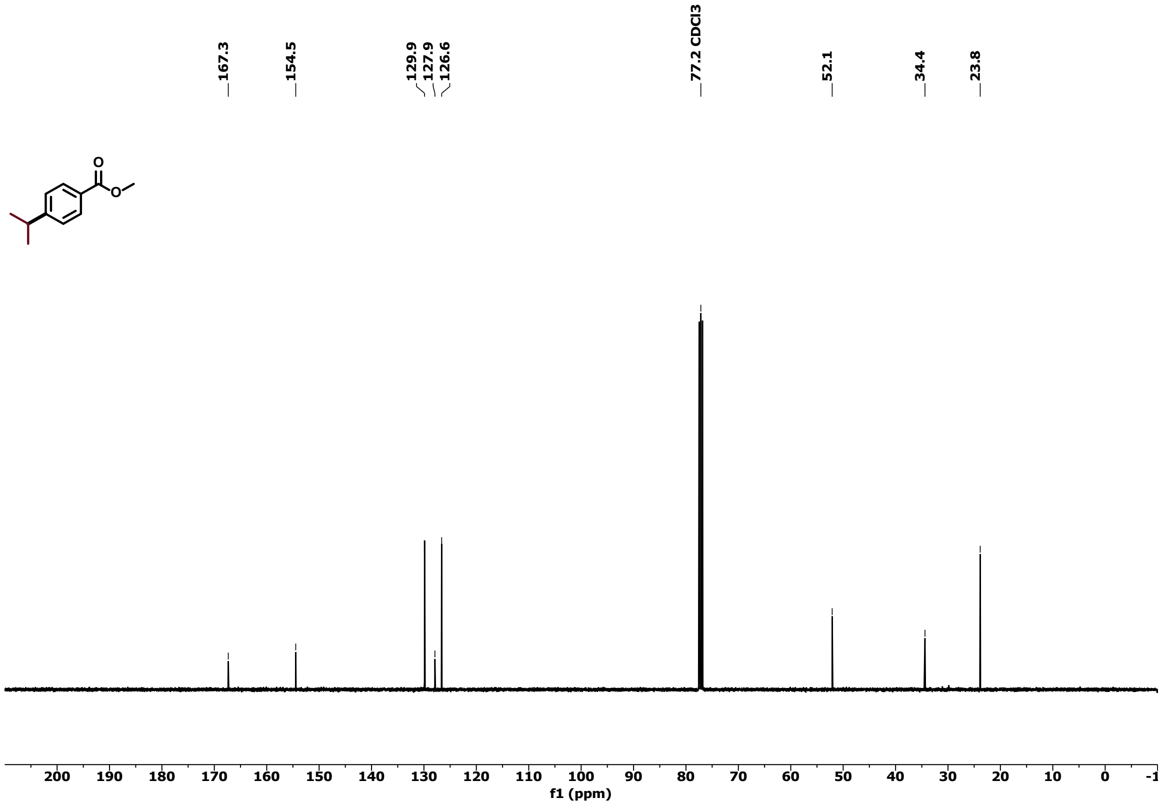

25, ^{1}H NMR (400 MHz, $CDCl_3$)

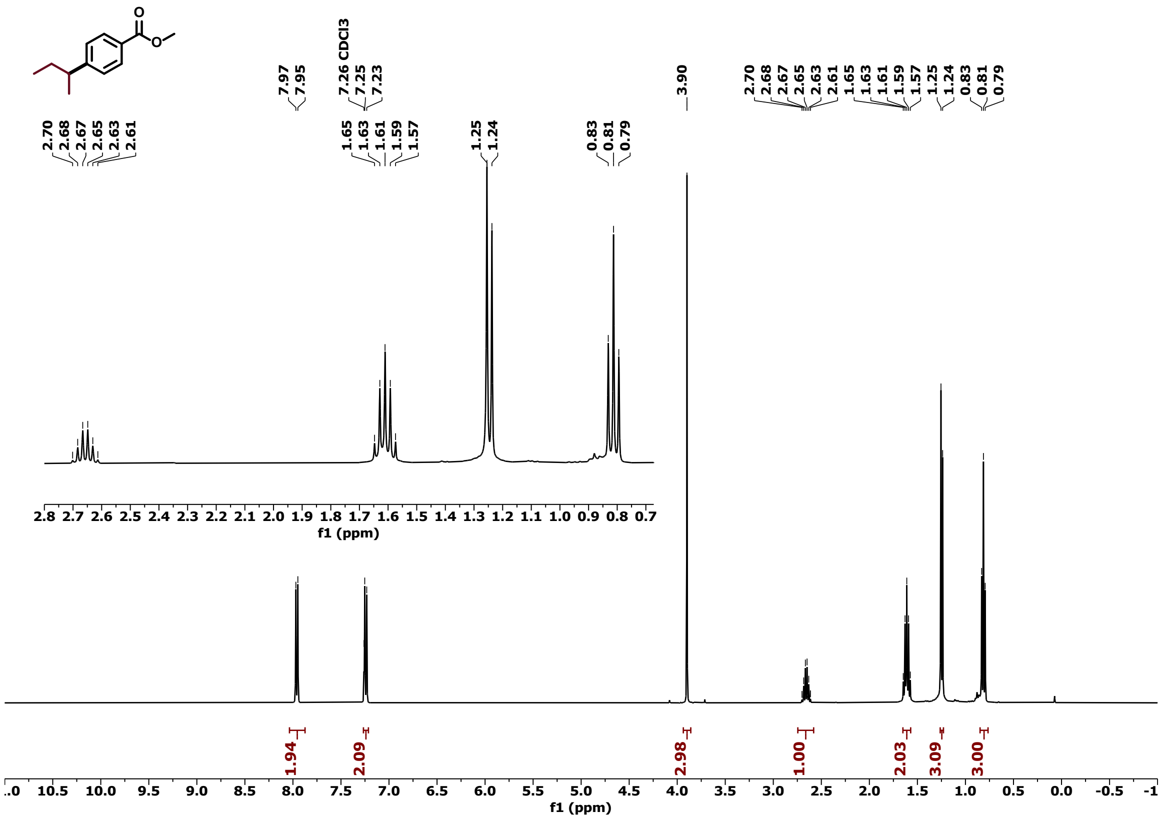

25, ^{13}C NMR (75 MHz, $CDCl_3$)

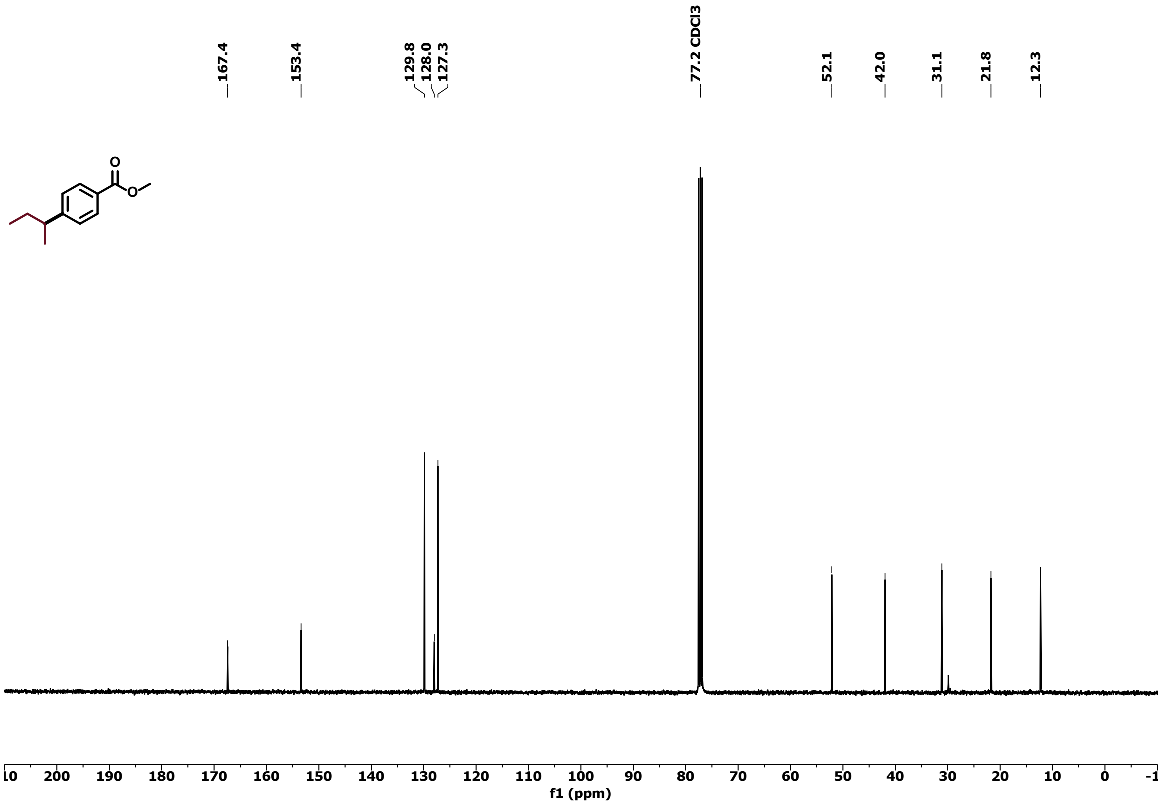

26, ^{1}H NMR (400 MHz, $CDCl_3$)

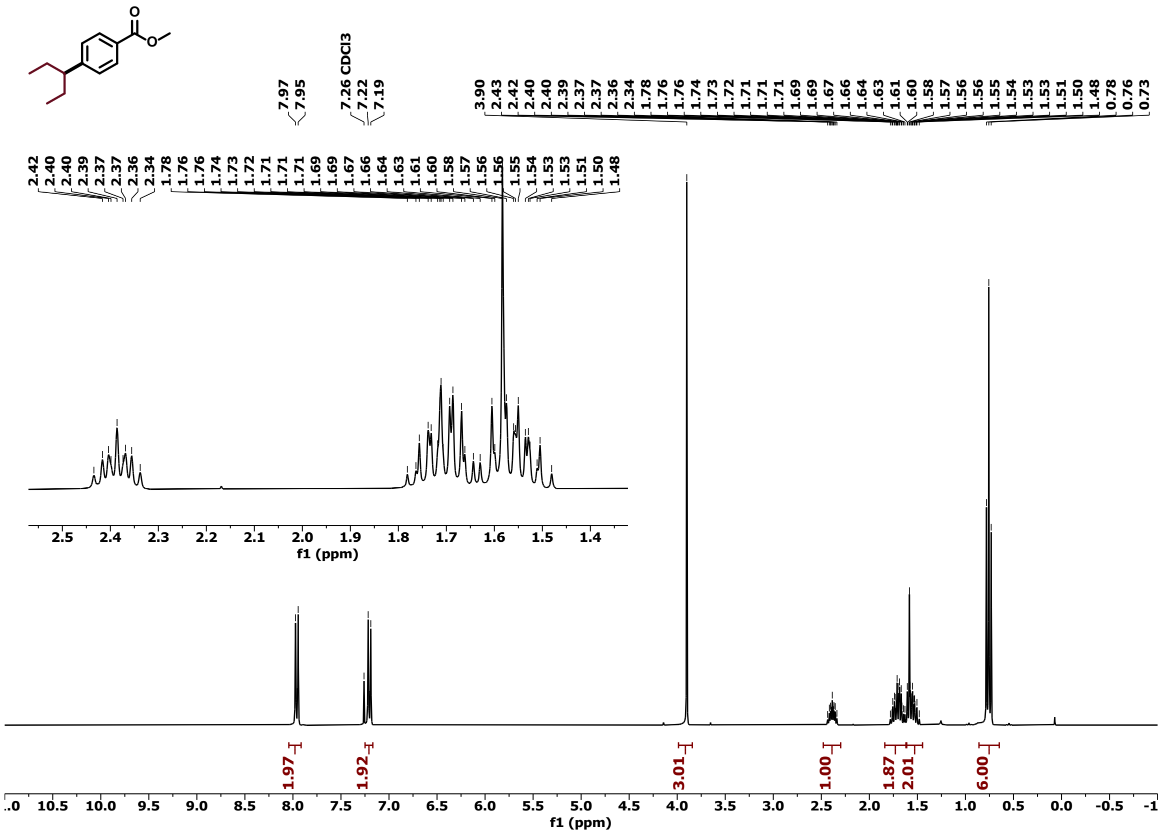

26, ^{13}C NMR (75 MHz, $CDCl_3$)

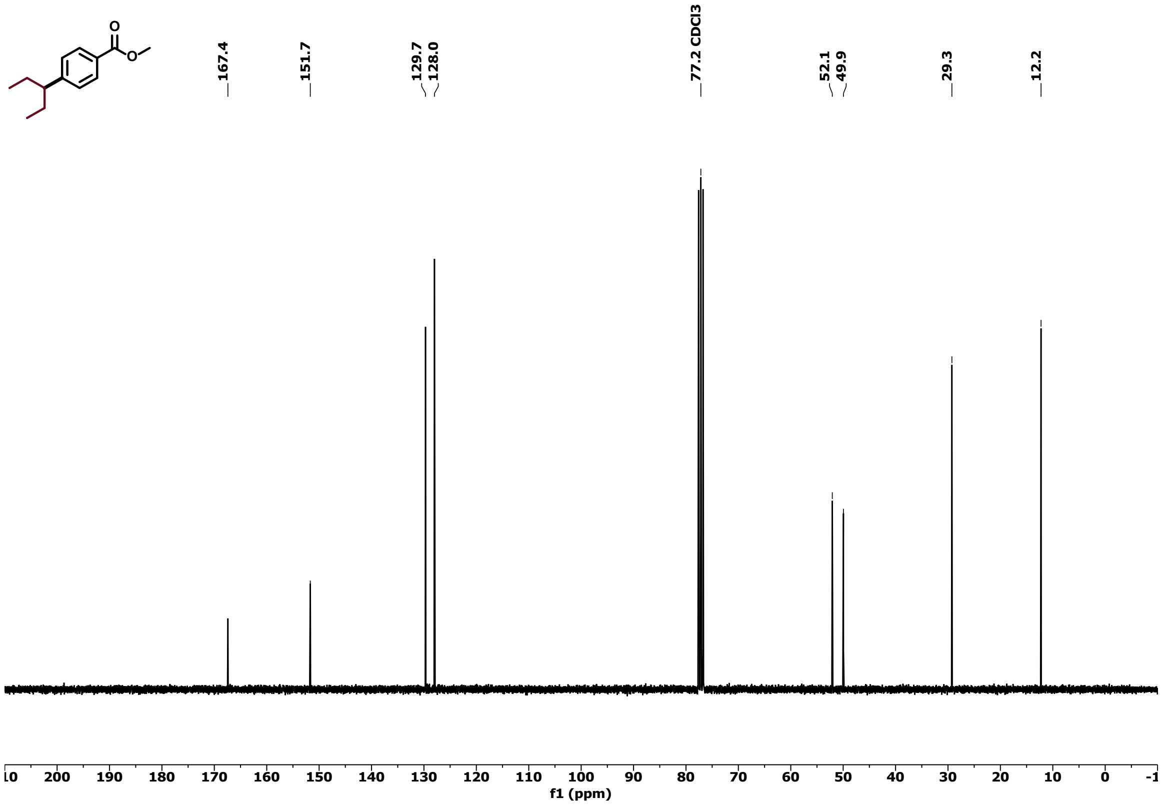

27, ^{1}H NMR (400 MHz, $CDCl_3$)

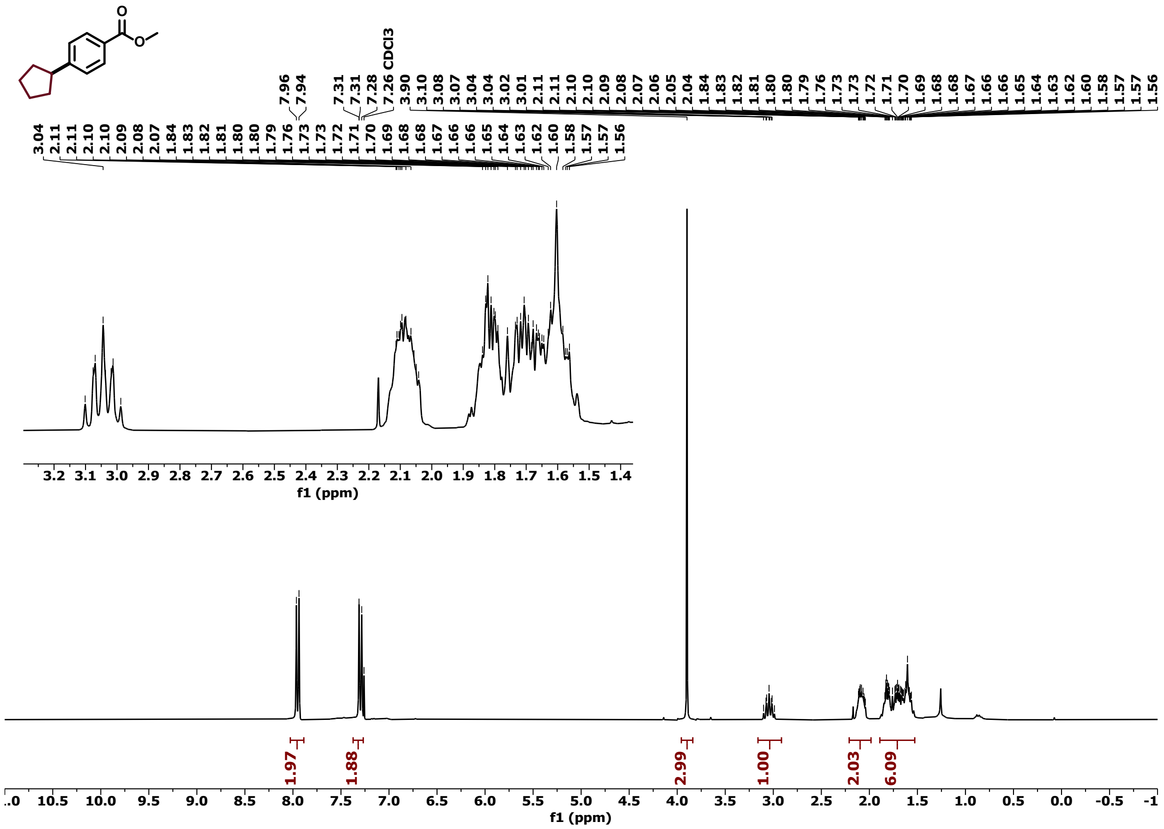

27, ^{13}C NMR (75 MHz, $CDCl_3$)

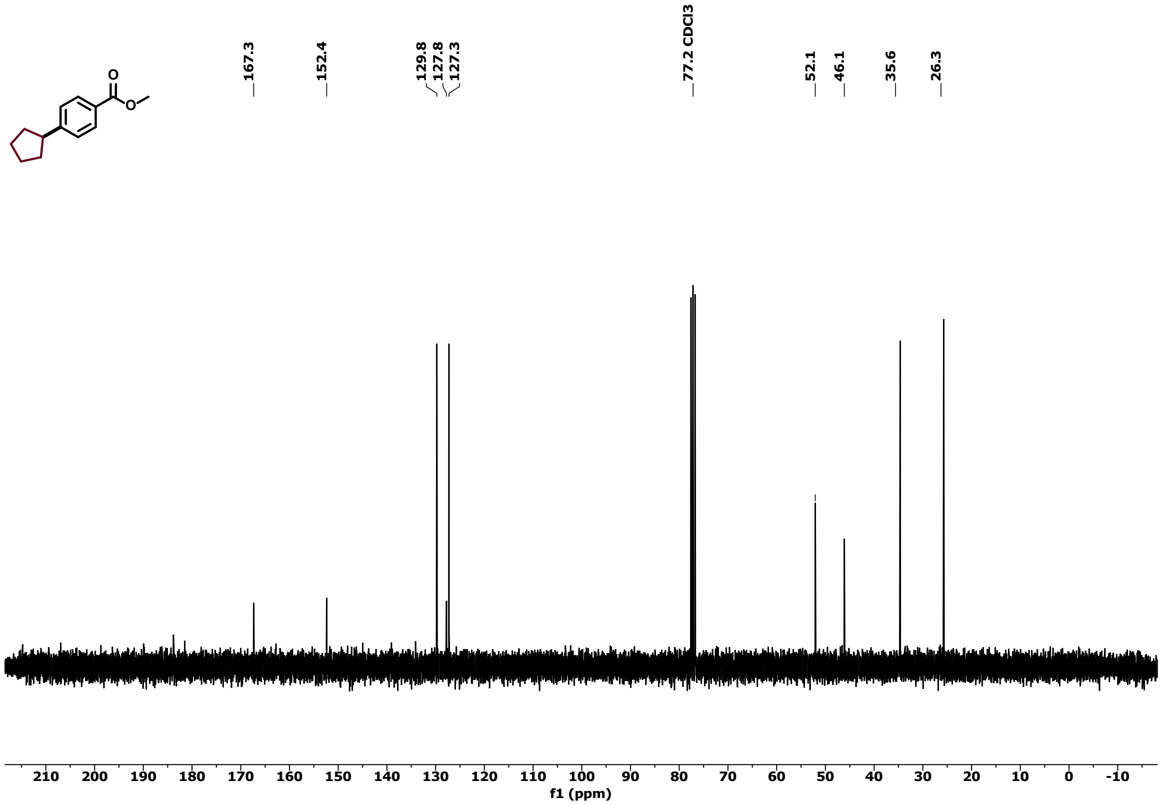

28, ^{1}H NMR (400 MHz, $CDCl_3$)

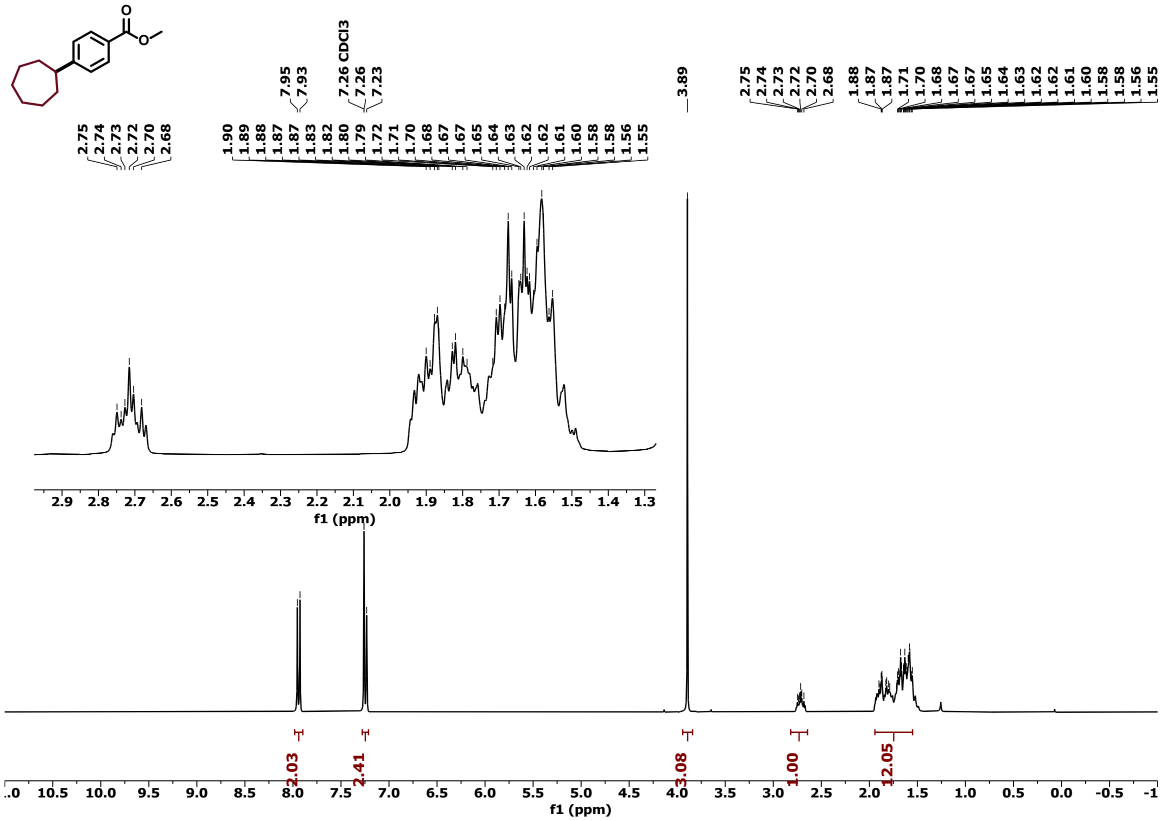

28, ^{13}C NMR (75 MHz, $CDCl_3$)

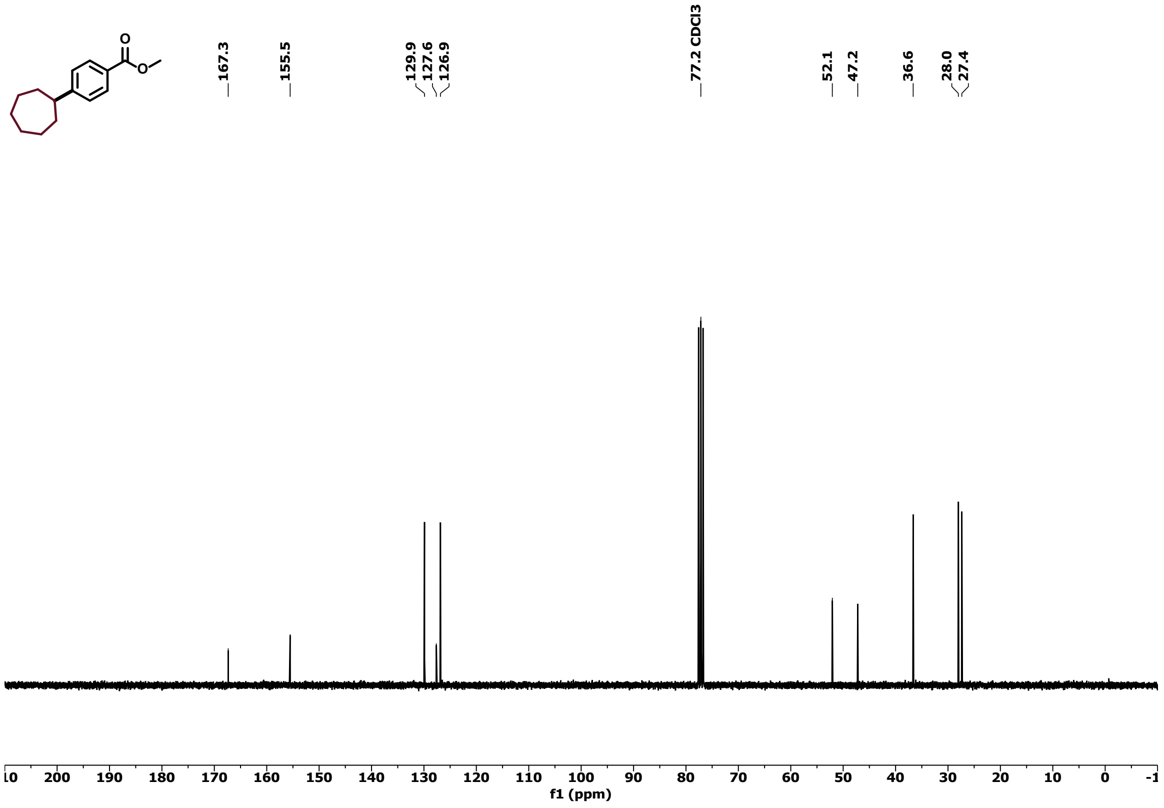

29, ^{1}H NMR (400 MHz, $CDCl_3$)

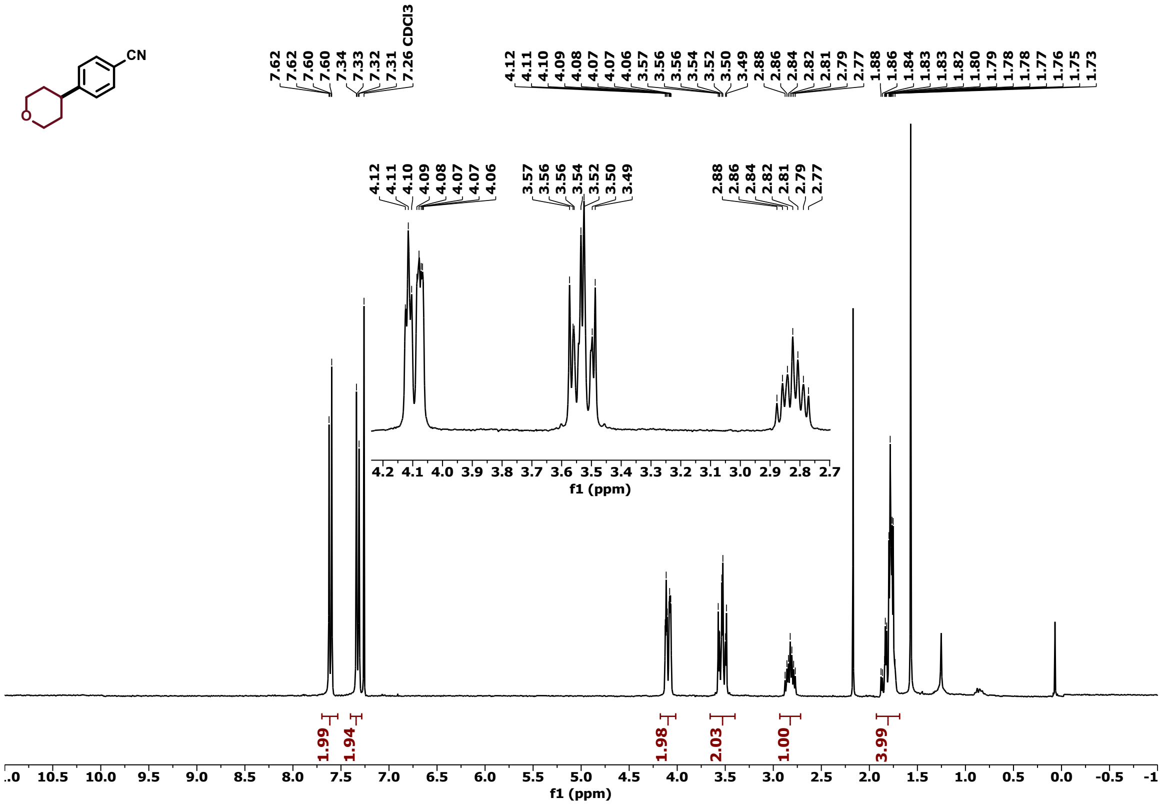

29, ^{13}C NMR (75 MHz, $CDCl_3$)

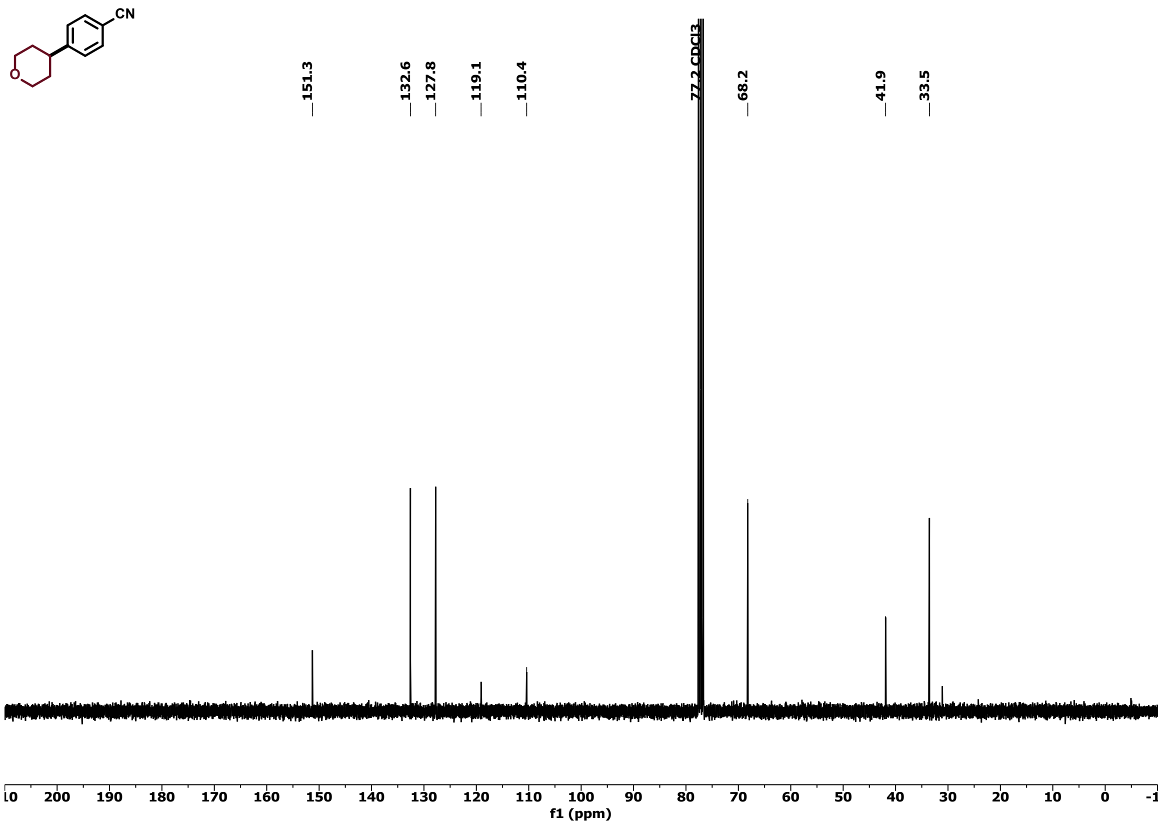

30, ^{1}H NMR (400 MHz, $CDCl_3$)

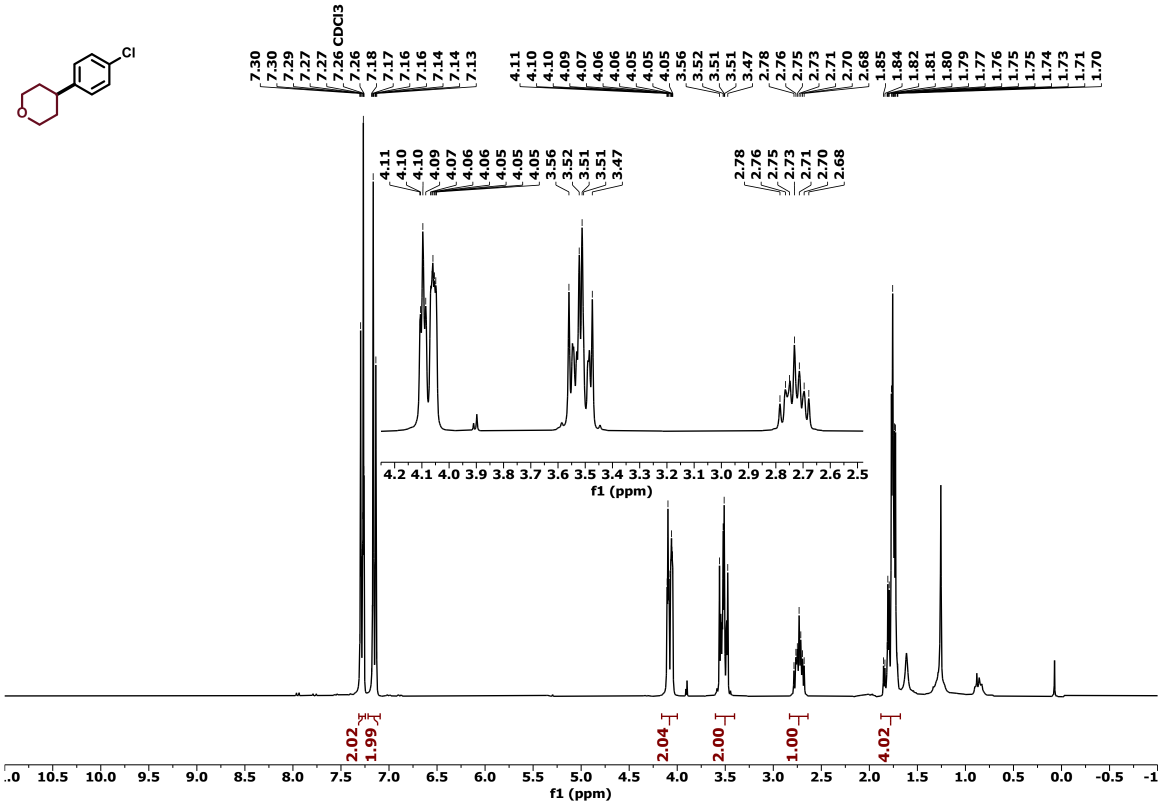

30, ^{13}C NMR (75 MHz, $CDCl_3$)

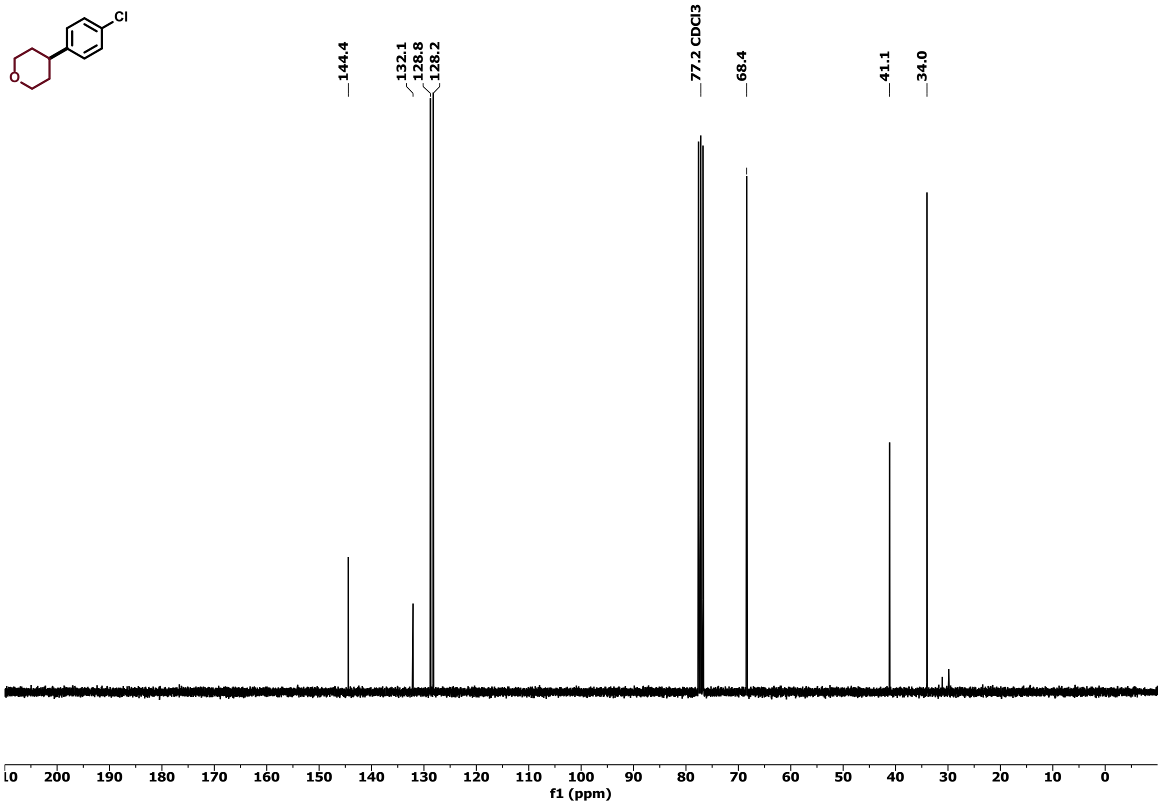

31, ^{1}H NMR (400 MHz, $CDCl_3$)

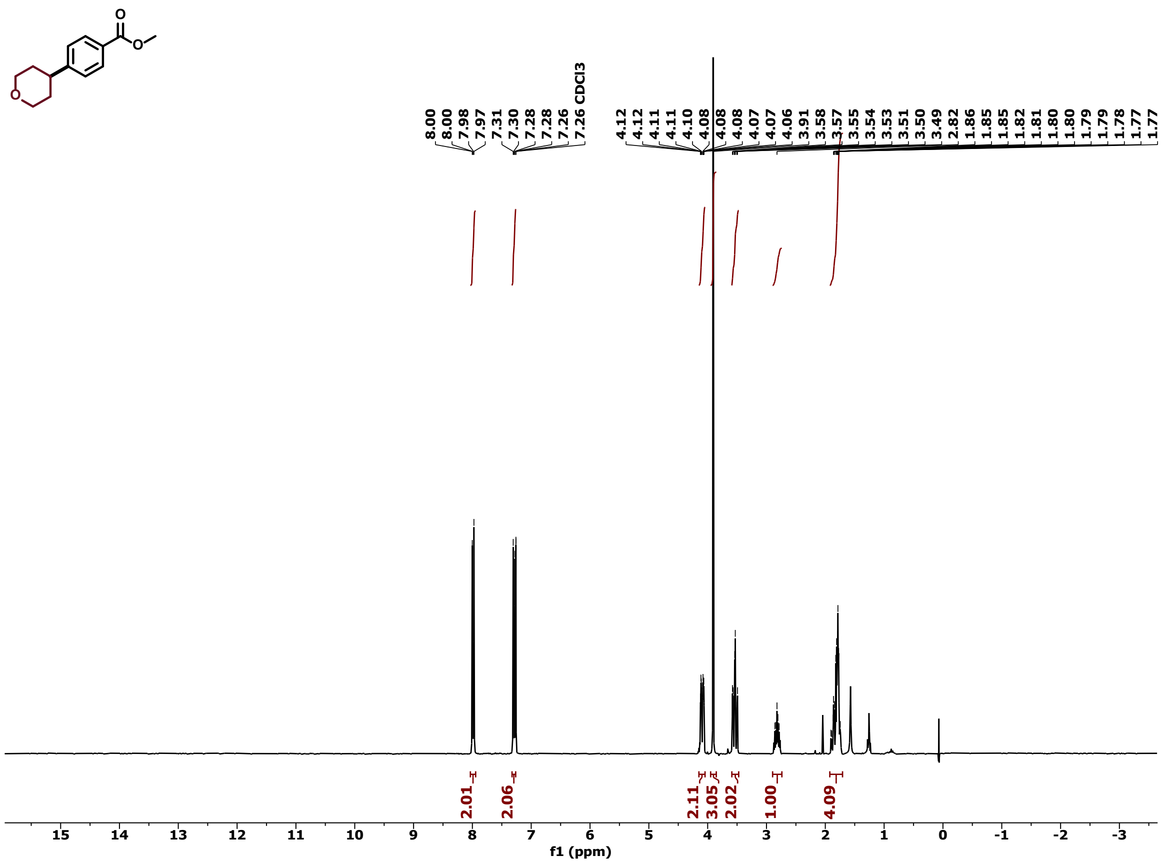

31, ^{13}C NMR (75 MHz, $CDCl_3$)

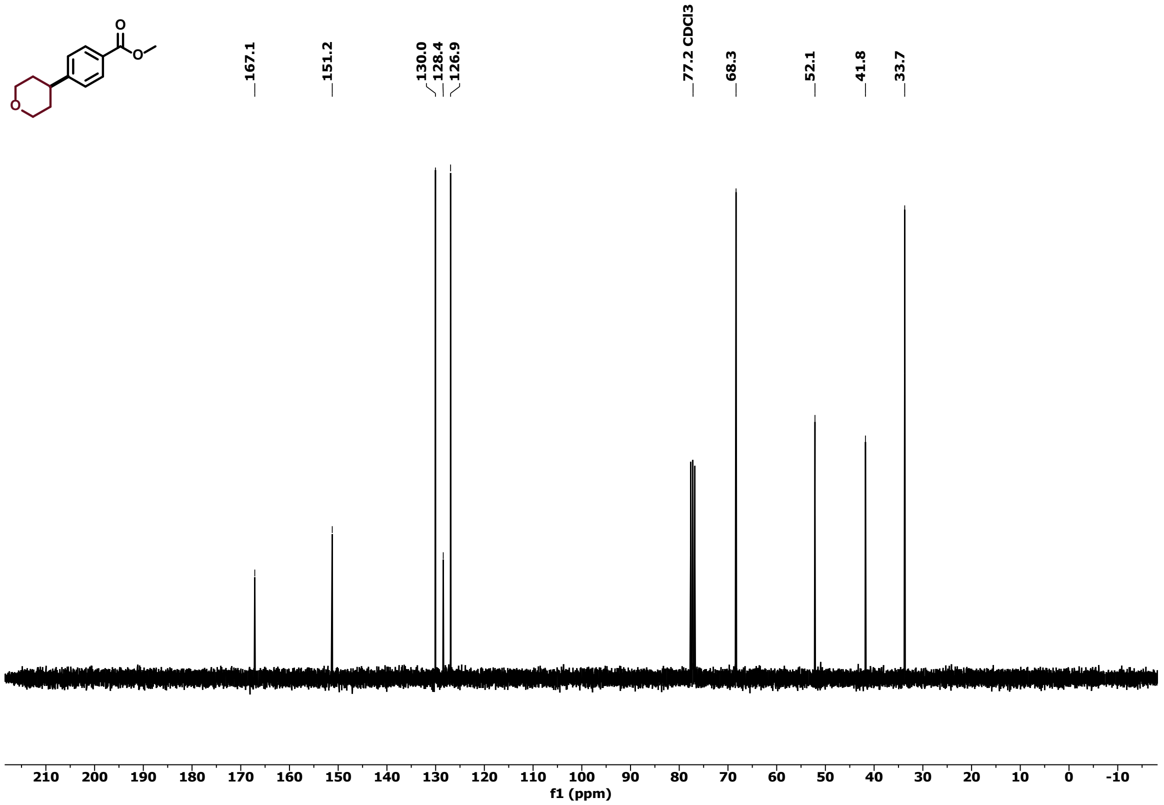

32, ^{1}H NMR (400 MHz, $CDCl_3$)

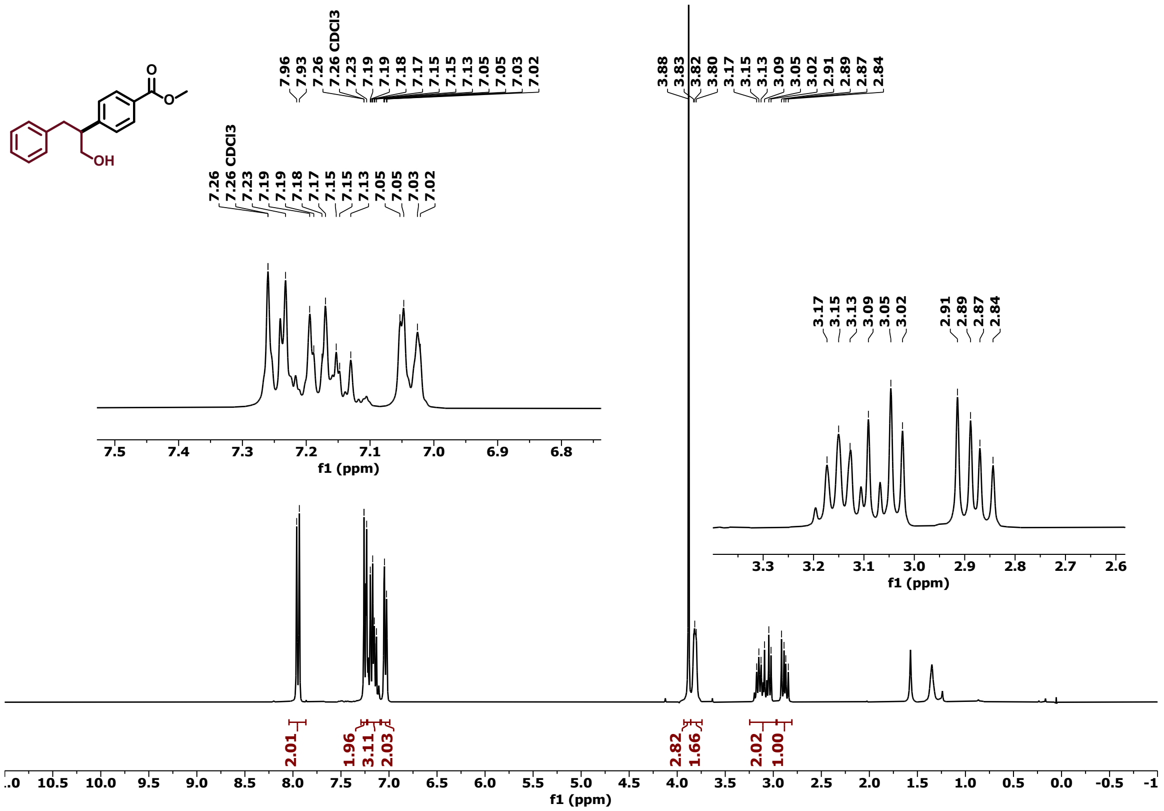

32, ^{13}C NMR (75 MHz, $CDCl_3$)

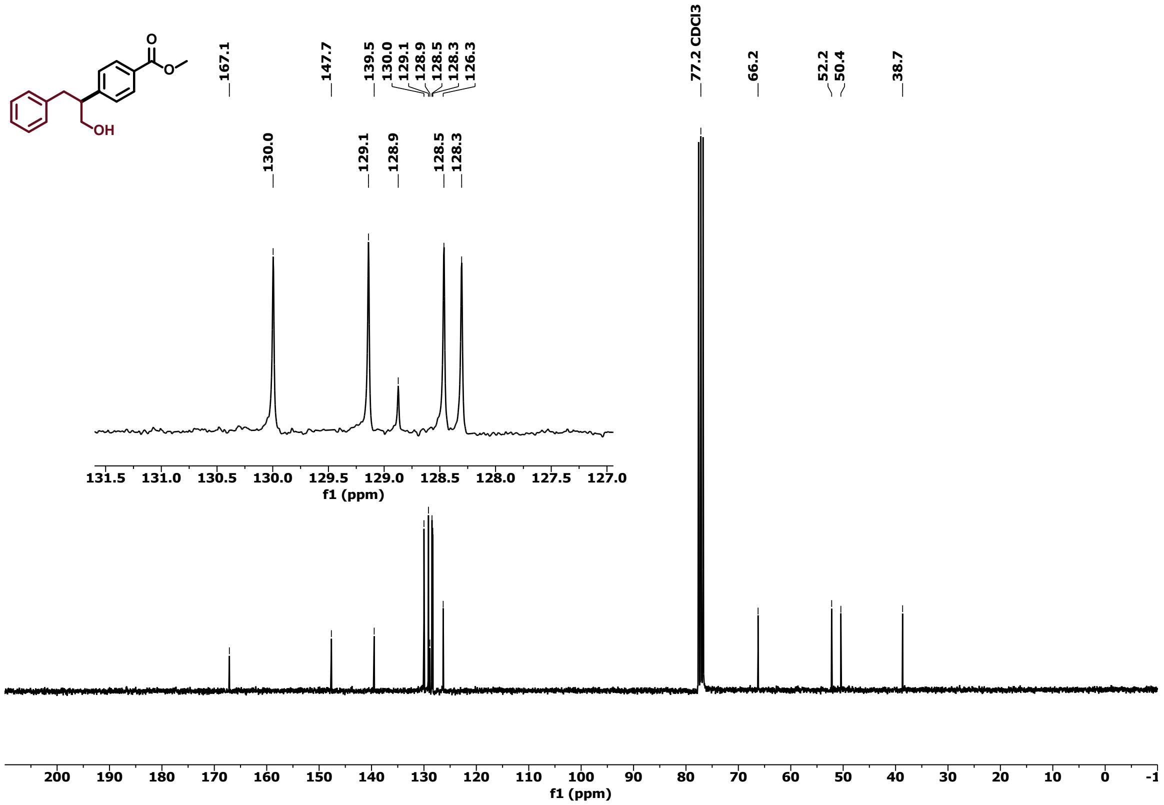

33, ^{1}H NMR (400 MHz, $CDCl_3$)

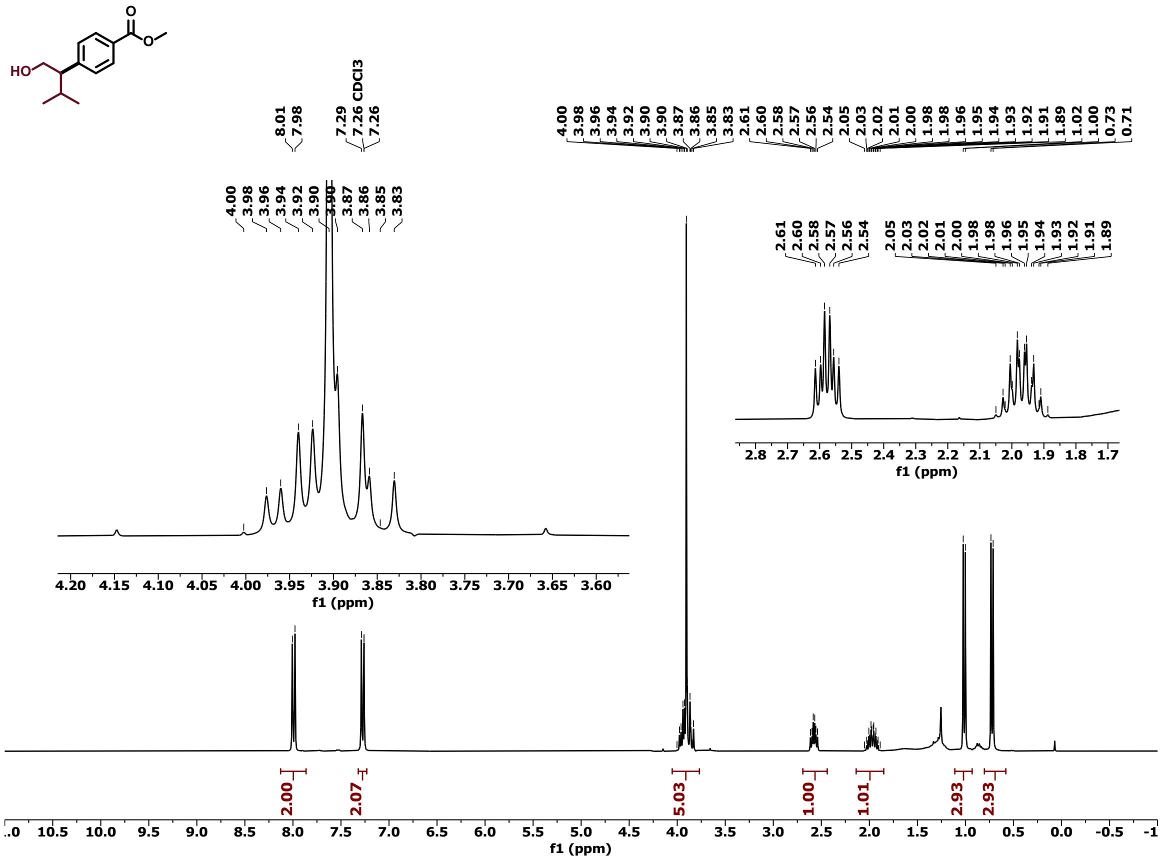

33, ^{13}C NMR (75 MHz, $CDCl_3$)

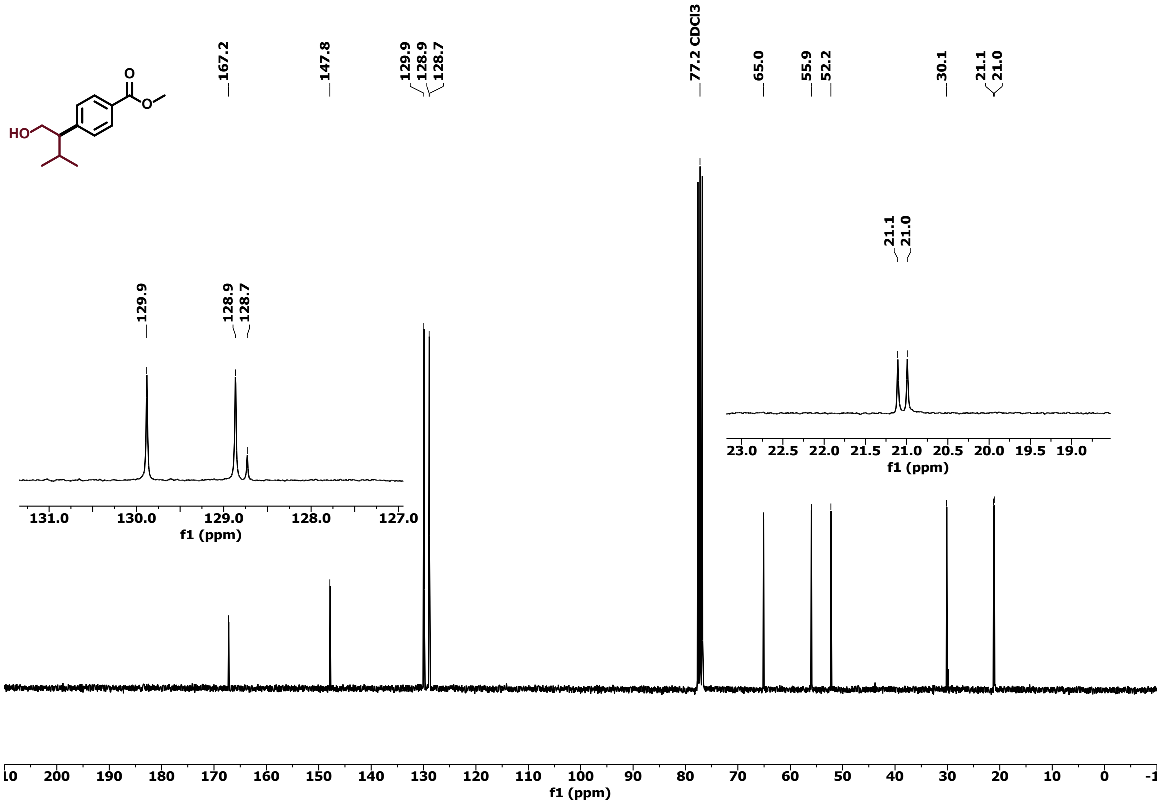

34a, iso-1 ^{1}H NMR (400 MHz, $CDCl_3$)

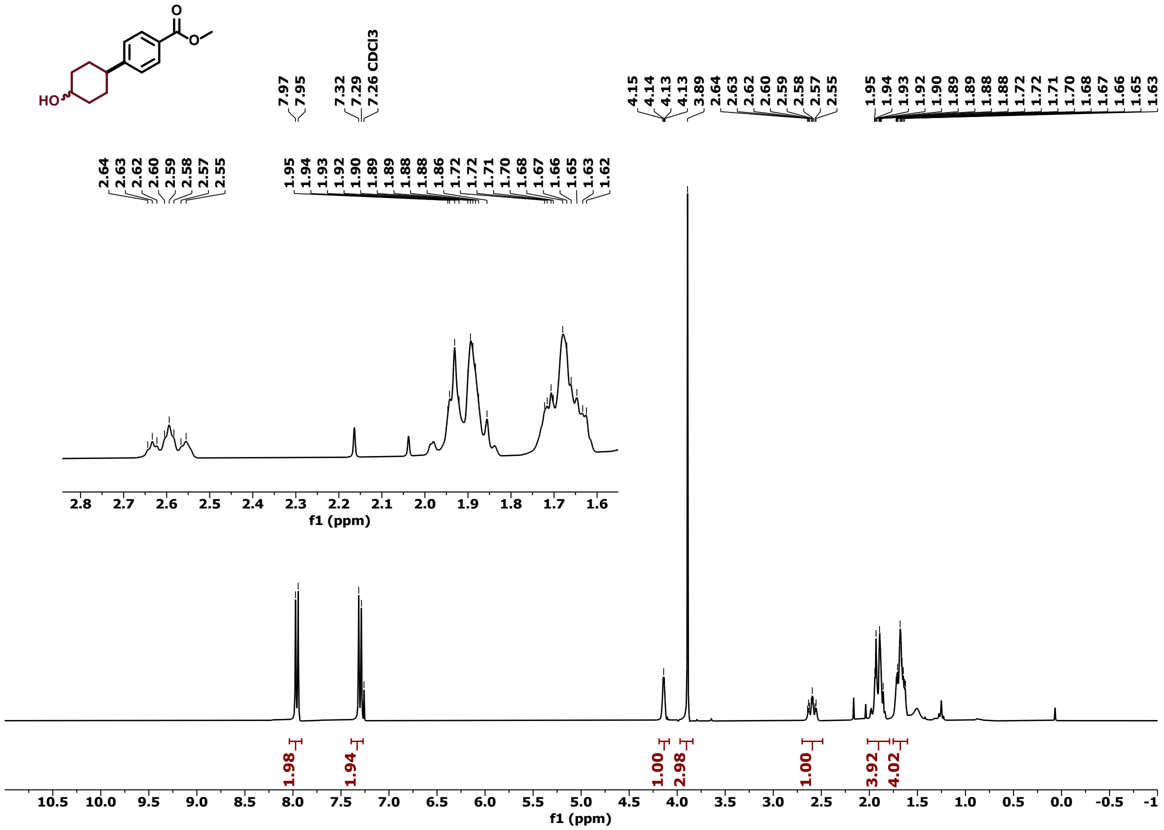

34a, iso-1 ^{13}C NMR (75 MHz, $CDCl_3$)

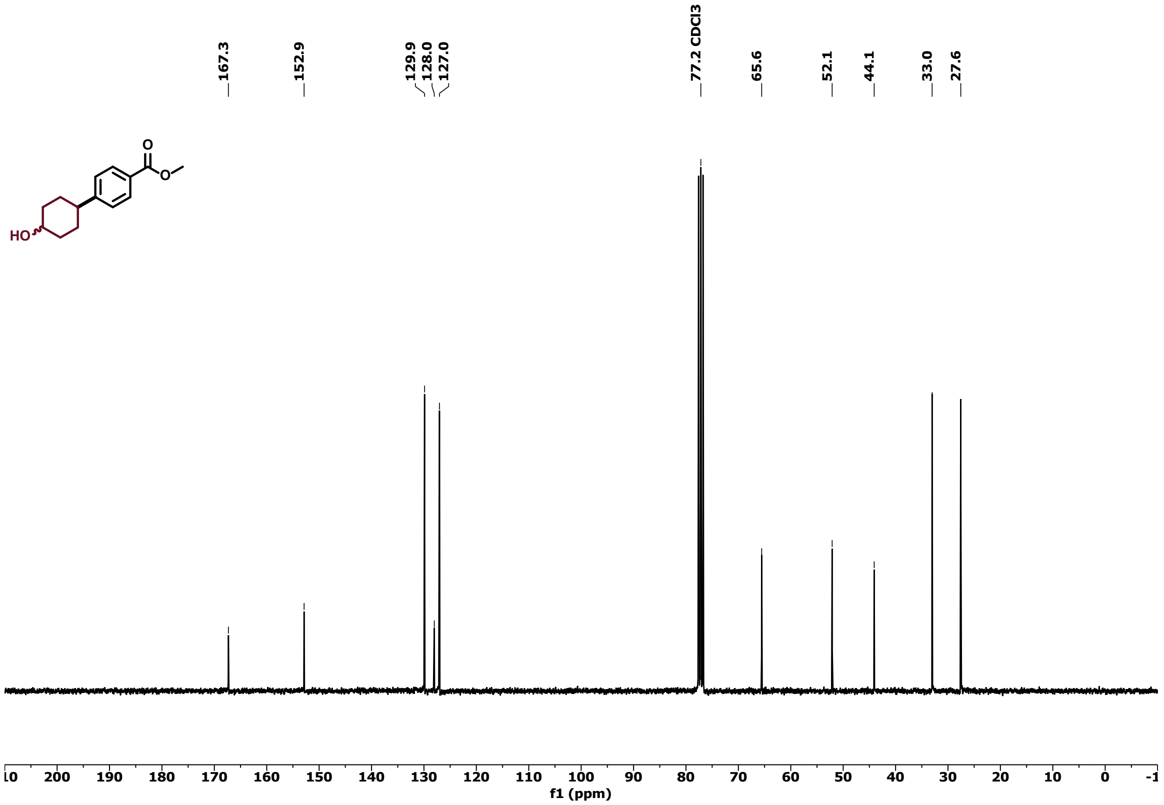

34b, iso-2 ^{1}H NMR (400 MHz, $CDCl_3$)

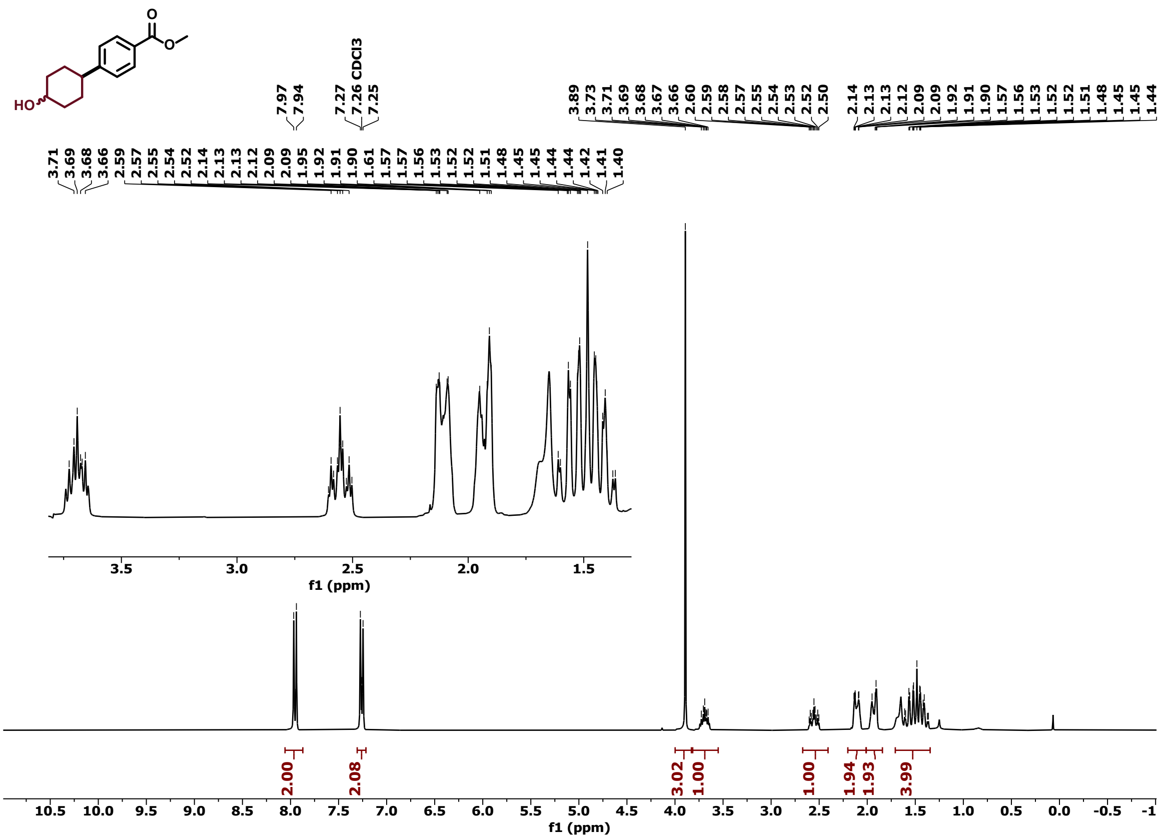

34b, iso-2 ^{13}C NMR (75 MHz, $CDCl_3$)

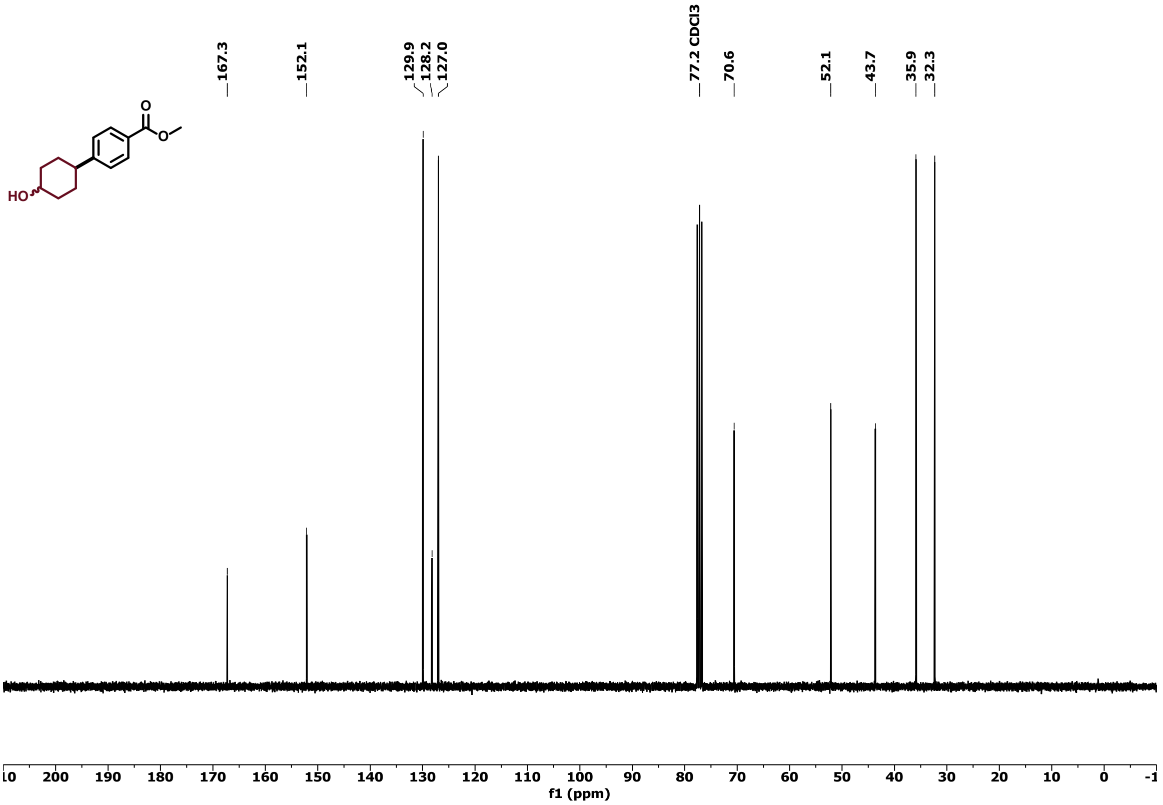

35, ^{1}H NMR (400 MHz, $CDCl_3$)

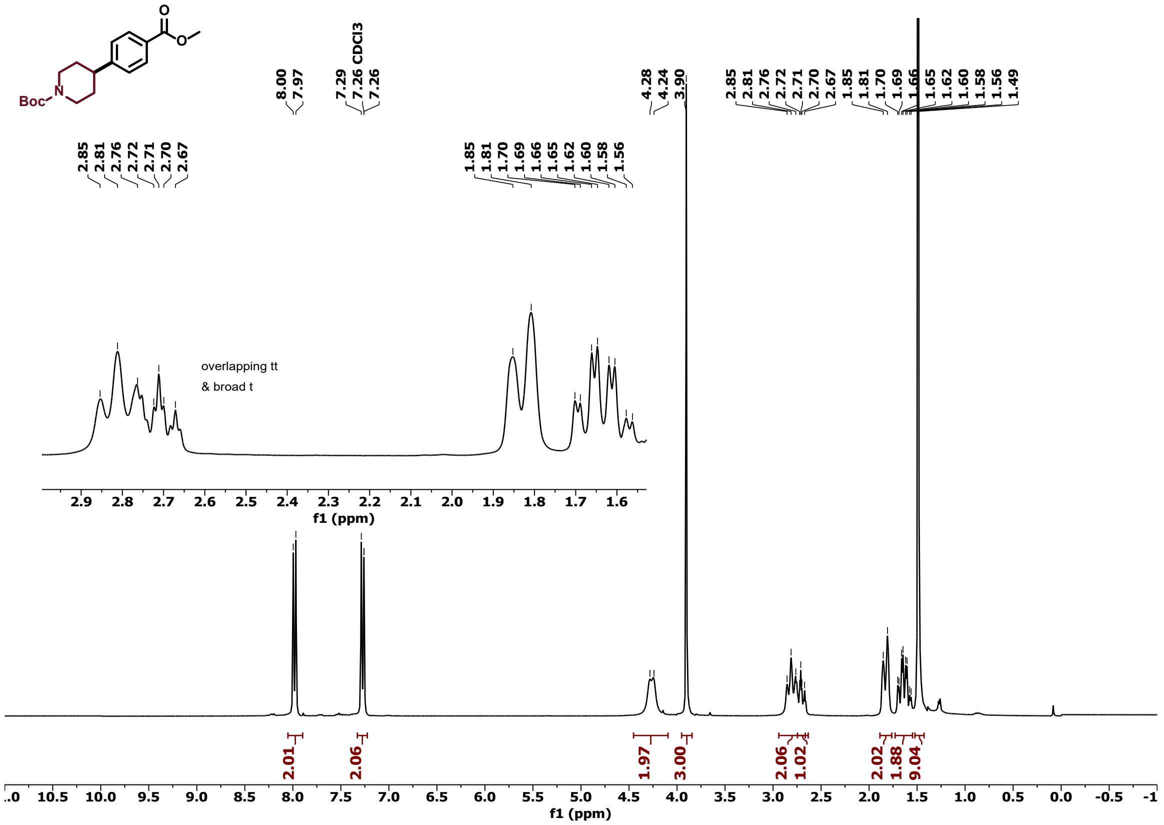

35, ^{13}C NMR (75 MHz, $CDCl_3$)

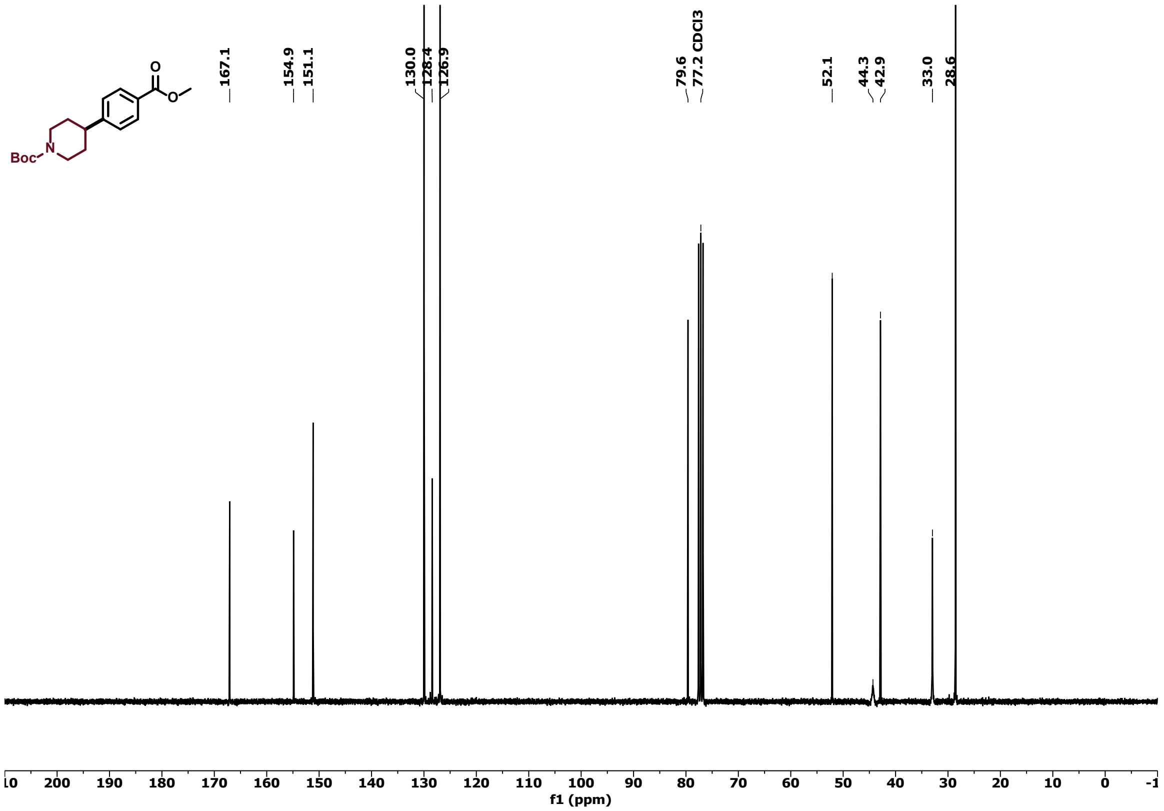

36, ^{1}H NMR (400 MHz, $CDCl_3$)

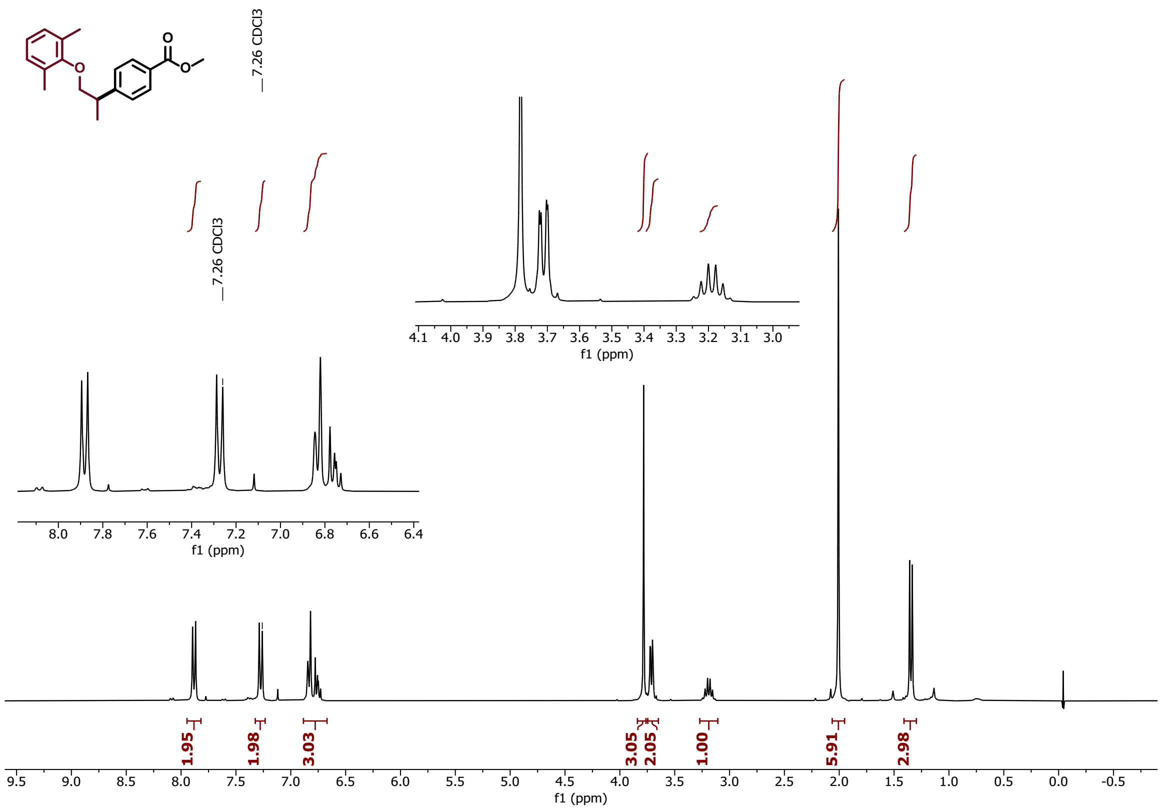

36, ^{13}C NMR (75 MHz, $CDCl_3$)

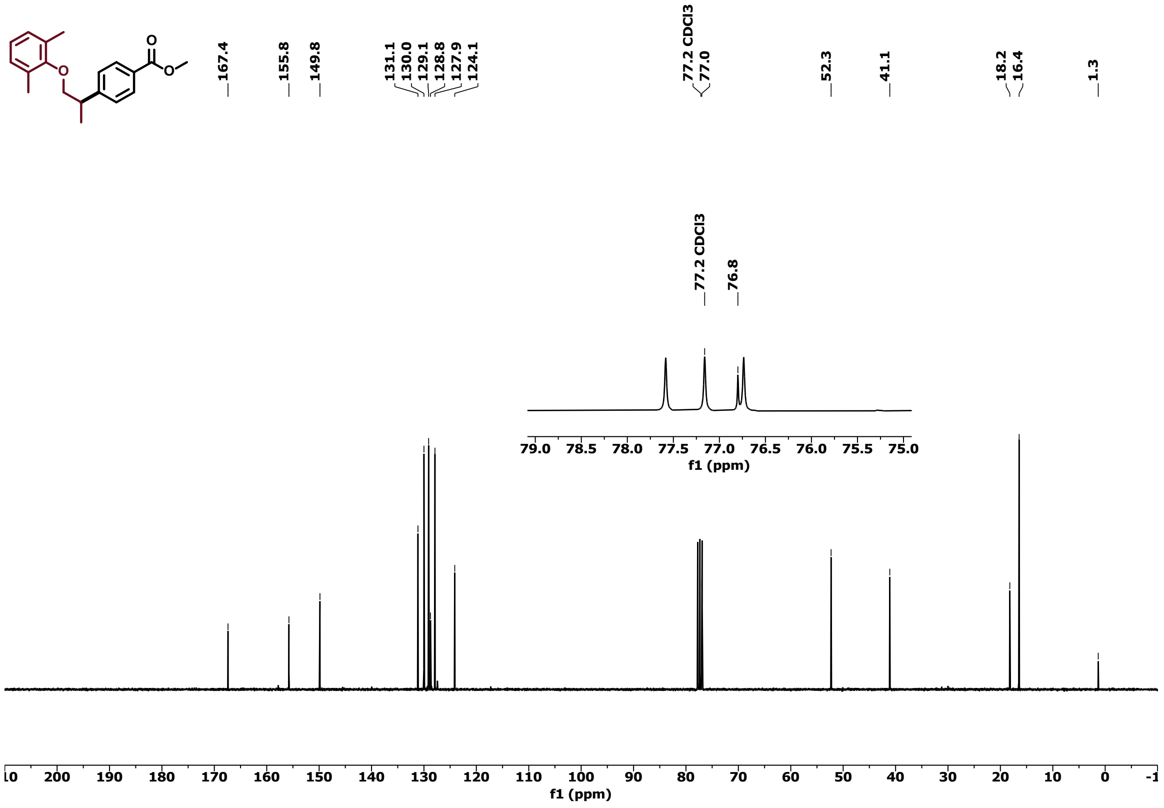

Id, ^{1}H NMR (300 MHz, $CDCl_3$)

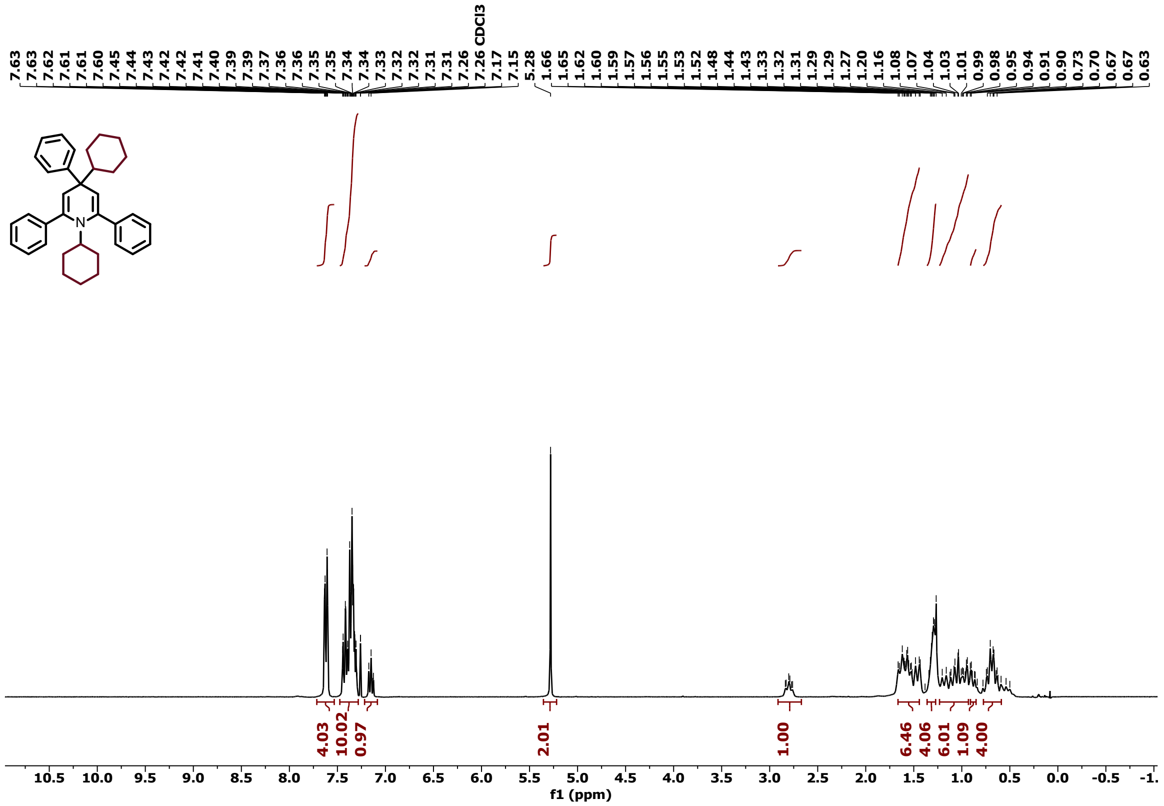

Supplement: Supplementary file 2 — jo3c00859_si_002.pdf [file jo3c00859_si_002.pdf]
